# Mining for Candidate Genes Related to Pancreatic Cancer Using Protein-Protein Interactions and a Shortest Path Approach

**DOI:** 10.1155/2015/623121

**Published:** 2015-11-03

**Authors:** Fei Yuan, Yu-Hang Zhang, Sibao Wan, ShaoPeng Wang, Xiang-Yin Kong

**Affiliations:** ^1^Institute of Health Sciences, Shanghai Institutes for Biological Sciences (SIBS), Chinese Academy of Sciences (CAS) & Shanghai Jiao Tong University School of Medicine (SJTUSM), Shanghai 200031, China; ^2^College of Life Sciences, Shanghai University, Shanghai 200444, China

## Abstract

Pancreatic cancer (PC) is a highly malignant tumor derived from pancreas tissue and is one of the leading causes of death from cancer. Its molecular mechanism has been partially revealed by validating its oncogenes and tumor suppressor genes; however, the available data remain insufficient for medical workers to design effective treatments. Large-scale identification of PC-related genes can promote studies on PC. In this study, we propose a computational method for mining new candidate PC-related genes. A large network was constructed using protein-protein interaction information, and a shortest path approach was applied to mine new candidate genes based on validated PC-related genes. In addition, a permutation test was adopted to further select key candidate genes. Finally, for all discovered candidate genes, the likelihood that the genes are novel PC-related genes is discussed based on their currently known functions.

## 1. Introduction

The pancreas is a significant versatile organ of the digestive system and endocrine system; it is significant because it assists in digestion and maintains hormonal balance via digestive enzymes and specific hormones, respectively. Pancreatic cancer (PC) originates from the pancreas, has a high rate of metastasis, and is a highly malignant tumor derived from pancreas tissue. Among various types of PC, pancreatic ductal adenocarcinoma (PDAC) accounts for more than 90% of all pancreatic tumors. PDAC is a malignancy with a poor prognosis, which is demonstrated through its one-year survival rate of approximately 18% for all stages of the disease [1]. In the Western world, pancreatic cancer is one of the top killers for human beings [2]. In 2012 alone, it resulted in 33000 deaths all over the world. In the Western world, pancreatic cancer is the fourth leading cause of death from cancer with a poor prognosis (5-year survival in less than 5% of cases according to most reports). Such a high fatality rate is attributed to the low rate of diagnosis at early age. Only a minority of patients can receive proper treatment with a 5-year survival rate up to 22%. Therefore, it is significant and crucial to study this severe disease. Similar to most types of tumors, PC is induced by both environmental and hereditary elements. Extrinsic factors such as age, gender, race, cigarette smoking, and obesity are all factors that may contribute to tumor initiation [3–5]. Further, certain chronic pancreas-associated diseases, such as diabetes mellitus and chronic pancreatitis, are also related to PC [6, 7].

Over the last decade, the genetic background for PC, especially pancreatic adenocarcinoma (PAC) that comprises most cases, has been revealed through validating a list of oncogenes and tumor suppressor genes for PC. Based on hereditary features, mutations in pancreatic adenocarcinoma can be divided into two clusters defined as common somatic mutations and germline mutations. A somatic mutation, KRAS, is regarded as the earliest and key mutation in nonfamilial PAC initiation, and it aids in maintaining invasion status and tumor progression [8]. In addition to tumor development, more mutated genes contribute to malignant phenotypes. The tumor suppressor genes p16/INK4A are significant somatic mutations and are downregulated in pancreatic adenocarcinoma [9, 10]. In many types of tumors, excessive activation of the TGF-*β* pathway is a mechanism of tumor progression and invasion [11]. Another pair of tumor suppressor genes, SMAD/DPC4, is involved in PAC through regulating the TGF-*β* pathway and is critical to advanced tumors [12].

Further, heredofamilial pancreatic adenocarcinoma is associated with certain other significant genes with more complex mechanisms. As indicated by the available literature, most such genes participate in the DNA repair process, such as MSH1/2, PMS1/2, and BRCA1/2, which may participate in nonspecific tumor induction [13–15]. However, in several inherited familial conditions, specific known mutations have not been identified, which may hint at the complexity of carcinogenic mechanisms and the potential oncogenes as well as tumor suppressor genes [16].

To predict more potential tumor-related genes, we proposed a new method that considers protein interactions from STRING (Search Tool for the Retrieval of Interacting Genes/Proteins) [17] and mines potential PC-related genes. STRING is a database with massive amounts of information on physical and functional associations between different proteins. With an established score system, STRING enables a user to search and browse the protein interactions data as well as simultaneously quantify the statistic cooccurrence in the background [17]. Here, depending on the method and using a comprehensive analysis of the protein interaction network, we fully utilized the database containing reported PC-related genes and predicted potential genes involved in PC.

## 2. Materials and Methods

### 2.1. Materials

PC-related genes were retrieved from KEGG PATHWAY, which is one of the main databases in KEGG (Kyoto Encyclopedia of Genes and Genomes) [18]. By examining the pathway hsa05212, pancreatic cancer (http://www.genome.jp/dbget-bin/www_bget?hsa05212, accessed in December 2014), we obtained 65 PC-related genes, which comprise the gene set *S*. Detailed information on these genes is listed in Supplementary Material I available online at http://dx.doi.org/10.1155/2015/623121. 

### 2.2. Method for Mining New Candidate Genes in a Protein-Protein Interaction Network

Protein-protein interaction (PPI) information is useful for investigating protein-related and gene-related problems [19–25]. Most methods for predicting protein attributes are based on the notion that two proteins that interact always share similar attributes [19–23]. Because PC-related genes must share common features related to PC, it is reasonable to use PPI information to identify whether a gene is related to PC. Here, we adopted the PPI information reported in STRING (version 9.1, http://www.string-db.org/) [17], which is a large online database that reports known and predicted protein interactions. The PPIs reported in STRING are derived from the following four sources: (1) genomic context, (2) high-throughput experiments, (3) (conserved) coexpression, and (4) previous knowledge, which imply that the physical and functional associations of the proteins were measured. To extract PPI information for humans, we downloaded the file “protein.links.v9.1.txt.gz” and selected protein interactions beginning with “9606.”, which produced 2,425,314 human PPIs. Each PPI includes two proteins and one score, which measures the strength of the interaction in the range between 150 and 999. For the formulation, let us denote the score of an interaction between proteins *p*_1_ and *p*_2_ as *Q*_*i*_(*p*_1_, *p*_2_).

The aforementioned PPIs were used to construct a large network by taking proteins as nodes. Two nodes are adjacent if and only if the corresponding proteins comprise an interaction that is a member of the 2,425,314 PPIs. Furthermore, the interaction score should also be added to the constructed network. To generate compatibility between our network and a shortest path approach, each edge *e* was assigned a weight *w*(*e*) as follows: *w*(*e*) = 1000 − *Q*_*i*_(*p*_1_, *p*_2_), where *p*_1_ and *p*_2_ are corresponding proteins of the endpoints of *e*.

Two proteins that can interact with each other always share similar attributes [19–23]. Further, considering the interaction score, this notion can be generalized as follows: two proteins in an interaction with a high score are more likely to share similar attributes than those with a low score. Moreover, if we consider a series of proteins *p*_1_, *p*_2_,…, *p*_*n*_ such that two consecutive proteins can comprise an interaction with a high score, then these proteins may all share some common attributes. By mapping these proteins onto the constructed network, they may comprise a shortest path connecting *p*_1_ and *p*_*n*_, which is a path connecting *p*_1_ and *p*_*n*_ such that the summation of the weights of edges on the path is minimum. Thus, we searched all shortest paths connecting any two PC-related genes. For two consecutive nodes in each of these paths, their corresponding proteins can comprise a PPI with a high score because they lie on a shortest path. As mentioned above, they can share similar functions. For a specific shortest path *p*_1_, *p*_2_,…, *p*_*n*_, where *p*_1_ and *p*_*n*_ are encoded by PC-related genes, *p*_2_ shares similar functions with *p*_1_; that is, *p*_2_ may be encoded by an invalidated or known PC-related gene; *p*_3_ shares similar functions with *p*_2_; thus *p*_3_ may also share similar functions with *p*_1_ and may be encoded by an invalidated or known PC-related gene. This can be induced to *p*_4_, *p*_5_,…, *p*_*n*−1_. Thus, we extracted genes in these shortest paths and excluded those that were members of 65 PC-related genes. These genes were referred to as shortest path genes and deemed to have special relationships with PC. The similar scheme has been applied to extract novel genes or chemicals related to other diseases or some biological processes [26–29]. In addition, to distinguish those genes, each shortest path gene was assigned a value, which is referred to as betweenness and defined as the number of shortest paths that contain the gene. The betweenness indicates the direct and indirect associations between shortest path genes and PC-related genes [30].

### 2.3. Further Selection

By executing the method mentioned in Section 2.2, certain shortest path genes can be extracted from the constructed network. However, certain such genes may be false positives. To exclude this type of gene, the following method was adopted.

Certain nodes in the constructed network are general hubs; their corresponding genes may always receive a high betweenness value even if we randomly selected certain genes as PC-related genes, but the genes exhibit few relationships with PC. To exclude this type of gene among shortest path genes, we randomly produced 1,000 gene sets with the same size as *S* and compared the betweenness of the shortest path genes in these sets to *S*. The detailed procedures are described as follows.

(I) Randomly produce 1,000 gene sets, such as *S*_1_, *S*_2_,…, *S*_1000_, each of which with the same size as* S*, the set consisting of PC-related genes.
(II) The method described in Section 2.2 was executed for 1,000 rounds. For the *i*th round, the PC-related genes in* S* were replaced with the genes in *S*_*i*_, and the shortest paths connecting any pair of genes in *S*_*i*_ were searched, thereby counting the betweenness of each shortest path gene based on these shortest paths.
(III) For each shortest path gene, there were 1,000 betweenness on randomly produced gene sets and one betweenness on* S*. After comparing them, we generated a measurement referred to as permutation FDR, which defines the proportion of randomly produced gene sets on which the betweenness was larger than that on* S*.
(IV) To exclude shortest path genes with general hubs in the network and few relationships with PC, we excluded shortest path genes with permutation FDR values equal to or larger than 0.05. The remaining shortest path genes are referred to as candidate genes.

## 3. Results and Discussion

### 3.1. Shortest Path Genes

**Figure 1:**
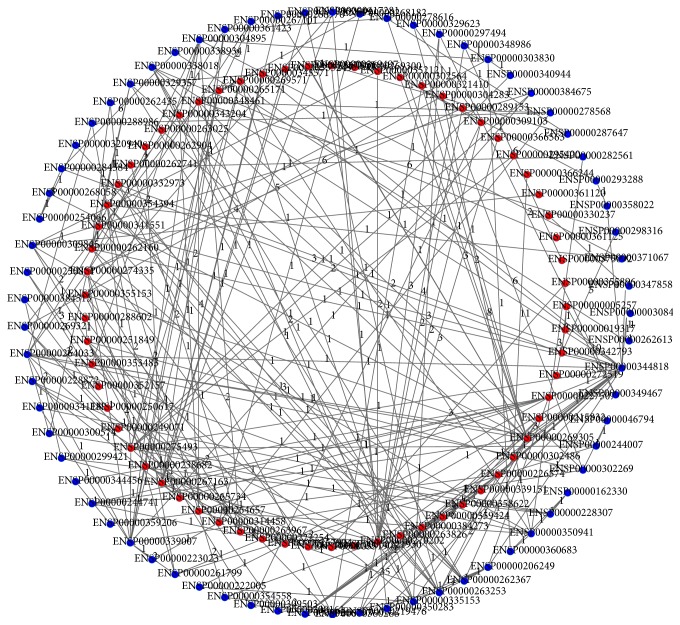
A graph consisting of 2,080 shortest paths. The nodes on the inner circle (red nodes) represent 65 PC-related genes, while the nodes on the outer circle (blue nodes) represent 69 shortest path genes. The numbers on the edges represent the weights of the edges.

Based on the method described in Section 2.2, we searched all shortest paths connecting any pair of PC-related genes and produced 2,080 shortest paths (each pair of PC-related genes can be connected by a shortest path), which are provided in Supplementary Material II. A graph with these 2,080 shortest paths is shown in Figure 1. The detailed information of edges in this graph is provided in Supplementary Material III. These 2,080 paths involved 134 Ensembl gene IDs. We excluded 65 Ensembl IDs for PC-related genes, resulting in 69 shortest path genes, which are listed in Supplementary Material IV. In addition, we counted the betweenness of each shortest path gene, which is also provided in Supplementary Material IV.

### 3.2. Candidate Genes

As mentioned in Section 3.1, several shortest path genes were retrieved. According to the principle underlying the method in Section 2.2, these genes may have special relationships with PC. However, certain such genes may be false positives and have few relationships with PC. Thus, we performed a permutation test to control for this type of genes. After calculating the permutation FDR for each shortest path gene, which are listed in Supplementary Material IV, we discarded the genes with a permutation FDR greater than or equal to 0.05, thereby generating thirteen candidate genes, which are listed in Table 1.

### 3.3. Analysis of Significant Candidate Genes

Using our method, we predicted thirteen genes that may participate in PC. Based on the principle underlying our method, the candidate genes are specifically connected with PC-related genes. Moreover, such genes have all been reported as genes that are relevant to PC and may exhibit diverse functions in tumor initiation and invasion, especially in PC.

Among the candidate genes, three are inhibitory genes that may participate in PC through their respective mechanisms. The first candidate gene is NFKBIA (see row 1 of Table 1, with betweenness 73 and permutation FDR 0.002), which is expressed in pancreatic tissues [31]. NFKBIA encodes a member of the NF-kappa-B inhibitor family, which has been confirmed to further participate in interactions with REL dimers to inhibit the NF-kappa-B pathway in processes of inflammation immune response and tumorigenesis [32, 33]. Targeted by a specific microRNA, miR196a, NFKBIA has been proved to be associated with PC, especially in the metastasis process [31]. As we have mentioned above, in most tumors including pancreatic cancer, NF-kappa-B pathway has been widely reported to be overactivated and has a close connection with the patients' prognosis, indicating the underlying relationship between NFKBIA and the pancreatic cancer [34–37]. Mutations have been widely reported to contribute to specific functional alteration of crucial proteins including NFKBIA in diseases especially in various cancer subtypes like pancreatic cancer [38–41]. What is more, as one of the crucial inhibitory components of NF-kappa-B pathway, NFKBIA has been reported to be downregulated in cancer [42, 43]. The overexpression of NFKBIA has also been reported to be associated with a better prognosis of various treatment methods in different human tumor subtypes, especially for the prognosis of patients that have taken alpha 1-adrenoceptor antagonists and radiotherapy [44, 45]. Further, considering the inflammation associated function of such gene, such mutations and expression alteration may contribute to the process of tumorigenesis through two individual regulation mechanisms: proliferation associated pathways that involve NF-kappa-B and specific immune response associated pathways in tumor microenvironment [46]. In addition to the NF-kappa-B pathway, the JNK pathway is also a specific pathway in tumor initiation and progression, including PC [47, 48]. A specific regulator of Rho protein exchange reactions which is crucial for JNK pathway, ARHGDIA (see row 2 of Table 1, with betweenness 94 and permutation FDR 0.012), was also identified using our method and functions in several types of tumors [49, 50]. In addition, this apoptosis inhibitory protein has been confirmed to control Rho protein homeostasis and participate in the initiation and progression of PC through the apoptosis associated GDP/GTP exchange reaction via RhoA-Rho pathway [51]. Apoptosis inhibition widely exists in tumor tissues and is an effective way to induce malignant cells, implying the potential relationship between ARHGDIA and pancreatic cancer [52, 53]. As we all know, pathways that contribute to the proliferation and invasion of cancer cells (JNK-STAT, RhoA-Rho signaling pathway, etc.) have all been confirmed to involve phosphorylation and dephosphorylation process which may be regulated by the survival associated GDP/GTP exchange reaction, further validating the potential relationship between ARHGDIA and pancreatic cancer [54–58]. What is more, regarded as a therapeutic target of pancreatic cancer, ARHGDIA (also known as RhoGDI) has been actually proved to be associated with the proliferation and invasion process of the pancreatic tumorigenesis which may further affect the prognosis of various patients [51]. The expression quantity of such gene, ARHGDIA, as the specific negative regulator of Rho protein exchange reactions, has been widely confirmed to be downregulated in tumors, which further induce the activation of survival associated Rho GTPases and promote the progression of various tumors [55–57]. As for specific mutations that may affect the biological function of such gene, coincidentally, specific triple Y156F/S101A/S174A-RhoGDI mutation has been confirmed to be related to the progression of pancreatic cancer [58]. Furthermore, we also obtained the gene XIAP as one of the candidate genes in our list (see row 3 of Table 1, with betweenness 64 and permutation FDR 0.006) that encodes a functional apoptosis suppressor protein. As the most potent apoptosis suppressor, XIAP interacts with several caspases directly and prevents the apoptosis process [59–61]. In PC, XIAP has been proved to be critical for the progression and prognosis of this disease via several classical pathways (Erk, PTEN/PI3K/AKT, etc.) [62–64]. What is more, the overexpression of XIAP has been confirmed to be associated with poor prognosis of pancreatic cancer. Therefore, such protein has already been applied as a useful laboratory test parameter and a functional therapeutic target of pancreatic cancer [64, 65]. As an immune associated functional gene, the polymorphism of XIAP may contribute to the complex and personalized immune response to pancreatic cancers. Some of the functional variants have already been proved to be directly associated with the prognosis of various subtypes of pancreatic cancer, indicating the potential significant role of XIAP in pancreatic cancer [62, 66].

In addition to inhibitory genes, a group of scaffold genes was also among the thirteen candidate genes. MAPK8IP3 (see row 4 of Table 1, with betweenness 64 and permutation FDR <0.001), which is also known as JIP3, is a scaffold protein involved in the JNK pathway [67]. Associated with the MAP kinase cascade, it may be related to PC via potential downstream mechanisms [67–69]. With high tissue specificity, such gene has been reported to actually induce the excessive proliferation of pancreatic cells, which may further initiate the biological process of pancreatic cancer [70]. MAPK8IP3 is also one of the crucial components of JNK pathway which has been overactivated in tumors including pancreatic tumor [71, 72]. During the initiation and invasion stages of pancreatic cancer, MAPK8IP3 has been reported to be overexpressed which may further contribute to the excessive proliferation of pancreatic cells in clinical cases [70]. Mutations have also been identified in MAPK8IP3 which has been reported to partially interfere with its own functions under pathological conditions [73, 74]. Some of the specific mutations of MAPK8IP3 have been reported to influence the adhesion and invasion process of various cancer cells including the pancreatic cancer cells, validating the crucial role of MAPK8IP3 in pancreatic cancer [75, 76]. As another functional scaffold protein that participates in the interaction between the cell membrane and cytoskeleton, our candidate gene SLC9A3R1 (see row 5 of Table 1, with betweenness 128 and permutation FDR 0.031) regulates the subcellular location and function of SLC9A3 and plays a specific role in the Wnt pathway [77–79]. Known as NHERF1, SLC9A3R1 acts as a scaffold protein in many types of tumors depending on its specific role in Wnt pathway, especially in PC [78, 80, 81]. During the course of pancreatic cancer raising, SLC9A3R1 has been confirmed to be overexpressed and involve the poor prognosis of such disease [81, 82]. Just like other candidates encoding scaffold proteins, SLC9A3R1 has also been proved to contain specific nonsynonymous mutations in various cancer subtypes, especially in pancreatic cancer [83, 84]. Coding an adaptor protein, the candidate gene PARVB (see row 6 of Table 1, with betweenness 64 and permutation FDR <0.001) is critical to certain specific biological processes in cancer, such as ERK signaling pathway and focal adhesion which both have been identified in pancreatic cancer [85, 86]. Therefore, this gene is critical for tumorigenesis and may have a specific role in PC [87]. As a crucial adaptor protein, the overexpression of PARVB has been proved to be associated with high cell proliferation rate which indicates worsening clinical prognosis of tumors, including pancreatic cancer [86, 88]. The polymerase of such gene, PARVB, has also been identified and confirmed to be associated with a group of severe diseases including cancers [87]. Such abnormal expression level and high polymerase in tumor cells suggest that PARVB may play a crucial role in the specific pathogenic process of pancreatic cancer.

Six remaining genes may also be associated with PC in their respective way. RARA (see row 7 of Table 1, with betweenness 24 and permutation FDR 0.005) encodes a specific receptor for retinoic acid, which may further regulate germ cell development during spermatogenesis and the expression of specific genes through recruiting the chromatin complex (KMT2E, MLL5, etc.) [89–91]. As we all know, the stem-cell-like features of tumor cells may be associated with the potential invasion and proliferation ability of such tumor cells [92–94]. In pancreatic cancer, it has been confirmed that the more “stem-like” the cancer cell is, the more malignant the cancer is [95]. Similar to its specific function in germ cells, RARA is related to the regulation of stem- cell-like features in PC, which implies that it may further affect the prognosis of such a severe malignancy [96]. The overexpression of RARA has been reported to be related to specific biological behaviors of tumors such as EMT (epithelial-to-mesenchymal) and invasion, which indicates poor prognosis of pancreatic cancers in clinical cases [97]. Although few mutations have been identified in RARA, crucial fusion gene variants have been widely identified in cancers which may be analyzed later. As we have mentioned above, SLC9A3R1 has been reported to regulate cellular morphology as a scaffold protein [78]. Similarly, the candidate gene ILK1 (see row 8 of Table 1, with betweenness 64 and permutation FDR 0.005) also mediates cell architecture, signaling transduction and cell adhesion via integrin-mediated signaling transduction, especially in tumor tissues [98, 99]. Although studies have not explained clearly how ILK1 actually contributes to the process of pancreatic cancer, it has been shown to participate in several types of tumors and may be associated with several unique molecules (GSK, AKT, PTEN, etc.) that all have been confirmed to be critical to PC [98–103]. Recently, such gene has been reported to be associated with the expression of TGF-*α*, which may partially explain the underlying carcinogenic mechanism of such gene [104]. What is more, such gene has been confirmed to be overexpressed during the whole biological process of tumorigenesis including the proliferation, migration, and invasion of pancreatic cancers [104–106]. Further, specific mutations have also been identified in such gene. Integrin-linked kinase 1 (ILK1) and HSP90 (HSP90AA1, which is also in our list and will be analyzed later) have both been confirmed to contain quite a lot of tumor-associated variants. Such mutations of ILK1 have been reported to be functional pancreatic cancer biomarkers [107–109]. All in all, ILK1 has been proved to regulate the tumorigenesis process of PC through a specific TGF-*α*-associated mechanism. As a functional SLC9A3R1-associated protein, a specific protein ezrin is encoded by one of our candidate genes EZR (see row 9 of Table 1, with betweenness 128 and permutation FDR 0.026) [110]. Crucial for cytoplasmic peripheral membrane, EZR is associated with the actin cytoskeleton and regulates the surface expression of actin [111, 112]. It has been widely reported that ezrin is critical for the proliferation, metastasis, and invasion process of several tumor subtypes, especially of pancreatic cancer [113, 114]. The overexpression of EZR has been widely reported to be associated with the initiation, proliferation, and metastasis processes of tumor, which strongly affect the patients' prognosis especially in pancreatic cancer [115–117]. EZR, as the crucial scaffold protein which may interact with SLC9A3R1 that we have mentioned above, has also been reported to contain specific mutations that may contribute to the tumorigenesis process of various cancer subtypes including pancreatic cancer [118]. All in all, EZR may be a potential pancreatic-cancer-associated gene and may contribute to similar pathways with SL9A3R1 as we have mentioned above [110].

Further, we also predicted one of the most famous oncogenes HRAS (see row 10 of Table 1, with betweenness 113 and permutation FDR 0.033), which binds GDP/GTP and exhibits intrinsic GTPase activity [119]. Associated with several tumors and similar to its homologues (r-RAS, k-Ras, etc.), HRAS is a powerful oncogene that can initiate tumors through inducing excessive growth factor activation in cells and promoting malignant proliferation of tumor cells, especially in tumors in situ [120–122]. In PC, such gene has also been regarded as a main oncogene and is widely reported to be overexpressed during the entire clinical course of pancreatic cancer, validating our prediction of the potential pancreatic cancer-associated genes [123]. Several genetic alterations in HRAS (mutations, CNVs, etc.) have been identified and reported to be associated with such a severe type of cancer [124–126]. Recently, some of the crucial mutations have already been confirmed to directly contribute to the tumorigenesis of pancreatic cancer, indicating the underlying relationship between mutations of candidate genes and the process of tumorigenesis [127]. Heat shock proteins compose a unique group of proteins produced by cells to resist harmful and stressful conditions, including the tumor microenvironment, which contains less oxygen and a lower pH [128–130]. This cluster of proteins has been confirmed to be functional in tumor microenvironment and promote the progression of various tumor subtypes [131, 132]. One of the candidate genes, HSP90AA1 (see row 11 of Table 1, with betweenness 230 and permutation FDR 0.007), also encodes a specific heat shock protein, heat shock protein 90 kDa alpha, which has been reported to be expressed in the cytoplasm. Several types of heat shock proteins (such as HSP20, HSP70, and HSP90), including our predicted protein, are not only related to tumor genesis but specifically contribute to PC [133–135]. Heat shock proteins have also been confirmed to be overexpressed in pancreatic cancer, including HSP90 which is encoded by our predicted gene [136–140]. The overexpression of heat shock proteins has been confirmed to actually contribute to the tumorigenesis process of pancreatic cancer which validates our predication of HSP90AA1 as a functional candidate gene [133]. As a hormone-associated gene, our candidate gene INS-IGF2 (see row 12 of Table 1, with betweenness 64 and permutation FDR 0.031) is a readthrough gene of INS and IGF2. This gene may act as a posttranscriptional regulatory factor for the two genes INS and IGF2, which has been shown to be associated with several pancreatic-associated diseases, such as diabetes [141–143]. As a specific fusion gene, INS-IGF2 participates in several cancer types, especially in prostate cancer [144–146]. The fusion of a tumor-associated gene and a pancreas-related gene, INS-IGF2, probably participates in PC progression and invasion specifically. Recently, such fusion gene INS-IGF2 is reported to be specifically expressed in pancreatic islets especially in patients with autoimmune diseases under pathological conditions [147, 148]. Since immune response is quite significant for tumor surveillance, INS-IGF2 is definitely associated with the unique tumor-associated immune response and may further contribute to the prognosis of pancreatic cancer [148]. Although genes like RARA (mostly identified as FIP1L1/PML-RARA fusion gene) (we have mentioned above) and INS-IGF2 have not been reported to contain specific mutations that may be associated with pancreatic cancer, such fusion genes have both been confirmed to have abnormal gene functions which may directly contribute to the tumorigenesis of various cancer subtypes, including pancreatic cancer [149, 150]. The last candidate gene PRKCA (see row 13 of Table 1, with betweenness 175 and permutation FDR <0.001) encodes a specific kinase that has been reported in several types of tumors [151, 152]. As a widely reported oncogene, PRKCA contributes to the phosphorylation process of crucial tumor-associated proteins [153]. The overexpression of PRKCA has been proved to enhance the transformed proliferation and invasion process of pancreatic cancer which further definitely influences the prognosis of such disease [154, 155]. While at the same time, genes like PRKCA which encodes functional kinase may be strongly affected by genetic alterations especially by some specific nonsynonymous mutations [75, 156]. Therefore, the polymerase of gene PRKCA may definitely affect the function of protein kinase C which has also been identified in pancreatic cancer [156, 157]. However, recent studies show that, at least in vitro, PRKCA has a specific function in tumor suppression [156]. The complex role of PKC in cancer may reflect the complicated interactive relationship between PRKCA and PC [156, 158]. All in all, our predicted gene PRKCA has been confirmed to be definitely associated with pancreatic cancer, while, at the same time, the various functions of PRKCA imply the complex role of such gene in pancreatic cancer.

Consequently, all candidate genes obtained using our method play a critical role in tumor initiation and progression. Because the detailed regulation mechanisms of only few genes remain unclear, those candidate genes may be genes that are critical to PC. PC features genetic heterogeneity, and many familial hereditary related genes remain ambiguous [16]. Therefore, in addition to the confirmed genes, such as KRAS and p16/INK41 [8, 9], more genes must be screened and validated to clearly demonstrate the mechanisms of pancreatic adenocarcinoma. In conclusion, based on reported PC-related genes, the proposed method was effective at predicting candidate tumor-associated genes in PC.

## 4. Conclusions

In this study, we proposed a computational method to mine new candidate genes related to pancreatic cancer, which utilized protein-protein interaction information. The analyses of the obtained candidate genes indicate that they may be novel PC-related genes. Hopefully, this contribution will promote studies on pancreatic cancer and provide new hope for designing effective treatments.

## Supplementary Material

Supplementary Material I: lists 65 PC-related genes and their ensembl IDs.
Supplementary Material II: lists the detailed information of 2,080 shortest paths.
Supplementary Material III: lists edges in a graph consisting of shortest paths connecting any two PC-related genes.
Supplementary Material IV: lists 69 shortest path genes and their betweenness and permutation FDRs.

## Figures and Tables

**Table 1:** Detailed information on the thirteen candidate genes.

Row number	Ensembl ID	Gene symbol	Full name	Betweenness	Permutation FDR
1	ENSP00000216797	NFKBIA	Nuclear Factor of Kappa Light Polypeptide Gene Enhancer in B-Cells Inhibitor, Alpha	73	0.002
2	ENSP00000269321	ARHGDIA	Rho GDP Dissociation Inhibitor (GDI) Alpha	94	0.012
3	ENSP00000347858	XIAP	X-Linked Inhibitor of Apoptosis, E3 Ubiquitin Protein Ligase	64	0.006
4	ENSP00000250894	MAPK8IP3	Mitogen-Activated Protein Kinase 8 Interacting Protein 3	64	<0.001
5	ENSP00000262613	SLC9A3R1	Solute Carrier Family 9, Subfamily A (NHE3, Cation Proton Antiporter 3), Member 3 Regulator 1	128	0.031
6	ENSP00000384515	PARVB	Parvin, Beta	64	<0.001
7	ENSP00000254066	RARA	Retinoic Acid Receptor, Alpha	24	0.005
8	ENSP00000299421	ILK1	Integrin-linked kinase 1	64	0.03
9	ENSP00000338934	EZR	Ezrin	128	0.026
10	ENSP00000309845	HRAS	Harvey Rat Sarcoma Viral Oncogene Homolog	113	0.033
11	ENSP00000335153	HSP90AA1	Heat Shock Protein 90 kDa Alpha (Cytosolic), Class A Member 1	230	0.007
12	ENSP00000348986	INS-IGF2	INS-IGF2 Readthrough	64	0.031
13	ENSP00000284384	PRKCA	Protein Kinase C, Alpha	175	<0.001
