# Supplementary material for: Mining for Candidate Genes Related to Pancreatic Cancer Using Protein-Protein Interactions and a Shortest Path Approach
Source: Biomed Res Int. 2015 Nov 3;2015:623121. doi: 10.1155/2015/623121 (PMC4647023; doi:10.1155/2015/623121)
Supplement: Supplementary file 1 — Supplementary Material I: lists 65 PC-related genes and their ensembl IDs. Supplementary Material II: lists the detailed information of 2,080 shortest paths. Supplementary Material III: lists edges in a graph consisting of shortest paths connecting any two PC-related genes. Supplementary Material IV: lists 69 shortest path genes and their betweenness and permutation FDRs. [file 623121.f1.zip › Supplementary Material I.docx]

**Supplementary Material I.** 65 PC-related genes and their ensembl IDs

| **Gene symbol** | **Ensembl ID** |
| --- | --- |
| RALA | ENSP00000005257 |
| RALBP1 | ENSP00000019317 |
| MAPK1 | ENSP00000215832 |
| TGFB1 | ENSP00000221930 |
| PIK3R2 | ENSP00000222254 |
| NFKB1 | ENSP00000226574 |
| CCND1 | ENSP00000227507 |
| TGFB3 | ENSP00000238682 |
| RAC2 | ENSP00000249071 |
| ARHGEF6 | ENSP00000250617 |
| RAF1 | ENSP00000251849 |
| CDK4 | ENSP00000257904 |
| SMAD2 | ENSP00000262160 |
| PIK3R3 | ENSP00000262741 |
| E2F3 | ENSP00000262904 |
| MAPK3 | ENSP00000263025 |
| AKT3 | ENSP00000263826 |
| PIK3CA | ENSP00000263967 |
| STAT3 | ENSP00000264657 |
| EGF | ENSP00000265171 |
| CDK6 | ENSP00000265734 |
| RB1 | ENSP00000267163 |
| RAD51 | ENSP00000267868 |
| PIK3R5 | ENSP00000269300 |
| TP53 | ENSP00000269305 |
| ERBB2 | ENSP00000269571 |
| AKT1 | ENSP00000270202 |
| RALB | ENSP00000272519 |
| PIK3R1 | ENSP00000274335 |
| EGFR | ENSP00000275493 |
| BRAF | ENSP00000288602 |
| PIK3CB | ENSP00000289153 |
| TGFA | ENSP00000295400 |
| MAP2K1 | ENSP00000302486 |
| BCL2L1 | ENSP00000302564 |
| RAC3 | ENSP00000304283 |
| BAD | ENSP00000309103 |
| CDC42 | ENSP00000314458 |
| MAPK9 | ENSP00000321410 |
| CASP9 | ENSP00000330237 |
| SMAD3 | ENSP00000332973 |
| IKBKB | ENSP00000339151 |
| SMAD4 | ENSP00000341551 |
| PLD1 | ENSP00000342793 |
| JAK1 | ENSP00000343204 |
| E2F1 | ENSP00000345571 |
| RAC1 | ENSP00000348461 |
| TGFBR2 | ENSP00000351905 |
| PIK3CG | ENSP00000352121 |
| MAPK10 | ENSP00000352157 |
| MAPK8 | ENSP00000353483 |
| STAT1 | ENSP00000354394 |
| CDKN2A | ENSP00000355153 |
| E2F2 | ENSP00000355249 |
| TGFB2 | ENSP00000355896 |
| IKBKG | ENSP00000358622 |
| CHUK | ENSP00000359424 |
| RALGDS | ENSP00000361120 |
| VEGFA | ENSP00000361125 |
| TGFBR1 | ENSP00000364133 |
| ARAF | ENSP00000366244 |
| PIK3CD | ENSP00000366563 |
| BRCA2 | ENSP00000369497 |
| AKT2 | ENSP00000375892 |
| RELA | ENSP00000384273 |
