# Supplementary material for: Mining for Candidate Genes Related to Pancreatic Cancer Using Protein-Protein Interactions and a Shortest Path Approach
Source: Biomed Res Int. 2015 Nov 3;2015:623121. doi: 10.1155/2015/623121 (PMC4647023; doi:10.1155/2015/623121)
Supplement: Supplementary file 1 — Supplementary Material I: lists 65 PC-related genes and their ensembl IDs. Supplementary Material II: lists the detailed information of 2,080 shortest paths. Supplementary Material III: lists edges in a graph consisting of shortest paths connecting any two PC-related genes. Supplementary Material IV: lists 69 shortest path genes and their betweenness and permutation FDRs. [file 623121.f1.zip › Supplementary Material II.docx]

**Supplementary Material II.** Detailed information of 2,080 shortest paths

| **Weight of path** | **Path** |
| --- | --- |
| 1 | ENSP00000005257 ENSP00000019317 |
| 16 | ENSP00000005257 ENSP00000342793 ENSP00000284384 ENSP00000338934 ENSP00000262613 ENSP00000003084 ENSP00000344818 ENSP00000360266 ENSP00000215832 |
| 16 | ENSP00000005257 ENSP00000342793 ENSP00000284384 ENSP00000338934 ENSP00000262613 ENSP00000003084 ENSP00000344818 ENSP00000364133 ENSP00000221930 |
| 18 | ENSP00000005257 ENSP00000342793 ENSP00000298316 ENSP00000348461 ENSP00000278568 ENSP00000288986 ENSP00000261799 ENSP00000222254 |
| 16 | ENSP00000005257 ENSP00000342793 ENSP00000284384 ENSP00000338934 ENSP00000262613 ENSP00000003084 ENSP00000344818 ENSP00000216797 ENSP00000226574 |
| 15 | ENSP00000005257 ENSP00000342793 ENSP00000284384 ENSP00000338934 ENSP00000262613 ENSP00000003084 ENSP00000344818 ENSP00000227507 |
| 16 | ENSP00000005257 ENSP00000342793 ENSP00000284384 ENSP00000338934 ENSP00000262613 ENSP00000003084 ENSP00000344818 ENSP00000364133 ENSP00000238682 |
| 15 | ENSP00000005257 ENSP00000342793 ENSP00000298316 ENSP00000348461 ENSP00000269321 ENSP00000249071 |
| 22 | ENSP00000005257 ENSP00000349467 ENSP00000297494 ENSP00000270202 ENSP00000299421 ENSP00000384515 ENSP00000250617 |
| 16 | ENSP00000005257 ENSP00000349467 ENSP00000297494 ENSP00000335153 ENSP00000251849 |
| 16 | ENSP00000005257 ENSP00000342793 ENSP00000284384 ENSP00000338934 ENSP00000262613 ENSP00000003084 ENSP00000344818 ENSP00000227507 ENSP00000257904 |
| 15 | ENSP00000005257 ENSP00000342793 ENSP00000284384 ENSP00000338934 ENSP00000262613 ENSP00000003084 ENSP00000344818 ENSP00000262160 |
| 18 | ENSP00000005257 ENSP00000349467 ENSP00000297494 ENSP00000270202 ENSP00000263967 ENSP00000262741 |
| 17 | ENSP00000005257 ENSP00000342793 ENSP00000284384 ENSP00000338934 ENSP00000262613 ENSP00000003084 ENSP00000344818 ENSP00000227507 ENSP00000267163 ENSP00000262904 |
| 18 | ENSP00000005257 ENSP00000342793 ENSP00000284384 ENSP00000338934 ENSP00000262613 ENSP00000003084 ENSP00000344818 ENSP00000360266 ENSP00000215832 ENSP00000302486 ENSP00000263025 |
| 51 | ENSP00000005257 ENSP00000349467 ENSP00000297494 ENSP00000270202 ENSP00000219476 ENSP00000263826 |
| 17 | ENSP00000005257 ENSP00000349467 ENSP00000297494 ENSP00000270202 ENSP00000263967 |
| 15 | ENSP00000005257 ENSP00000342793 ENSP00000284384 ENSP00000282561 ENSP00000350941 ENSP00000264657 |
| 16 | ENSP00000005257 ENSP00000342793 ENSP00000284384 ENSP00000338934 ENSP00000262613 ENSP00000003084 ENSP00000344818 ENSP00000275493 ENSP00000265171 |
| 16 | ENSP00000005257 ENSP00000342793 ENSP00000284384 ENSP00000338934 ENSP00000262613 ENSP00000003084 ENSP00000344818 ENSP00000227507 ENSP00000265734 |
| 16 | ENSP00000005257 ENSP00000342793 ENSP00000284384 ENSP00000338934 ENSP00000262613 ENSP00000003084 ENSP00000344818 ENSP00000227507 ENSP00000267163 |
| 16 | ENSP00000005257 ENSP00000342793 ENSP00000284384 ENSP00000254066 ENSP00000268058 ENSP00000269305 ENSP00000267868 |
| 28 | ENSP00000005257 ENSP00000349467 ENSP00000297494 ENSP00000270202 ENSP00000352121 ENSP00000269300 |
| 15 | ENSP00000005257 ENSP00000342793 ENSP00000284384 ENSP00000254066 ENSP00000268058 ENSP00000269305 |
| 15 | ENSP00000005257 ENSP00000342793 ENSP00000284384 ENSP00000338934 ENSP00000262613 ENSP00000003084 ENSP00000344818 ENSP00000269571 |
| 15 | ENSP00000005257 ENSP00000349467 ENSP00000297494 ENSP00000270202 |
| 2 | ENSP00000005257 ENSP00000019317 ENSP00000272519 |
| 16 | ENSP00000005257 ENSP00000342793 ENSP00000284384 ENSP00000338934 ENSP00000262613 ENSP00000003084 ENSP00000344818 ENSP00000264033 ENSP00000274335 |
| 15 | ENSP00000005257 ENSP00000342793 ENSP00000284384 ENSP00000338934 ENSP00000262613 ENSP00000003084 ENSP00000344818 ENSP00000275493 |
| 17 | ENSP00000005257 ENSP00000349467 ENSP00000297494 ENSP00000335153 ENSP00000251849 ENSP00000288602 |
| 17 | ENSP00000005257 ENSP00000349467 ENSP00000297494 ENSP00000270202 ENSP00000289153 |
| 16 | ENSP00000005257 ENSP00000342793 ENSP00000284384 ENSP00000338934 ENSP00000262613 ENSP00000003084 ENSP00000344818 ENSP00000275493 ENSP00000295400 |
| 17 | ENSP00000005257 ENSP00000342793 ENSP00000284384 ENSP00000338934 ENSP00000262613 ENSP00000003084 ENSP00000344818 ENSP00000360266 ENSP00000215832 ENSP00000302486 |
| 17 | ENSP00000005257 ENSP00000342793 ENSP00000284384 ENSP00000338934 ENSP00000262613 ENSP00000003084 ENSP00000344818 ENSP00000358022 ENSP00000293288 ENSP00000302564 |
| 18 | ENSP00000005257 ENSP00000342793 ENSP00000298316 ENSP00000348461 ENSP00000269321 ENSP00000304283 |
| 17 | ENSP00000005257 ENSP00000349467 ENSP00000297494 ENSP00000270202 ENSP00000309103 |
| 15 | ENSP00000005257 ENSP00000342793 ENSP00000298316 ENSP00000348461 ENSP00000268182 ENSP00000314458 |
| 16 | ENSP00000005257 ENSP00000342793 ENSP00000284384 ENSP00000338934 ENSP00000262613 ENSP00000003084 ENSP00000344818 ENSP00000360266 ENSP00000321410 |
| 16 | ENSP00000005257 ENSP00000342793 ENSP00000284384 ENSP00000338934 ENSP00000262613 ENSP00000003084 ENSP00000344818 ENSP00000347858 ENSP00000330237 |
| 15 | ENSP00000005257 ENSP00000342793 ENSP00000284384 ENSP00000338934 ENSP00000262613 ENSP00000003084 ENSP00000344818 ENSP00000332973 |
| 15 | ENSP00000005257 ENSP00000342793 ENSP00000284384 ENSP00000338934 ENSP00000262613 ENSP00000003084 ENSP00000344818 ENSP00000339151 |
| 15 | ENSP00000005257 ENSP00000342793 ENSP00000284384 ENSP00000338934 ENSP00000262613 ENSP00000003084 ENSP00000344818 ENSP00000341551 |
| 2 | ENSP00000005257 ENSP00000342793 |
| 16 | ENSP00000005257 ENSP00000342793 ENSP00000284384 ENSP00000282561 ENSP00000350941 ENSP00000264657 ENSP00000343204 |
| 15 | ENSP00000005257 ENSP00000342793 ENSP00000284384 ENSP00000338934 ENSP00000262613 ENSP00000003084 ENSP00000344818 ENSP00000345571 |
| 13 | ENSP00000005257 ENSP00000342793 ENSP00000298316 ENSP00000348461 |
| 16 | ENSP00000005257 ENSP00000342793 ENSP00000284384 ENSP00000338934 ENSP00000262613 ENSP00000003084 ENSP00000344818 ENSP00000364133 ENSP00000351905 |
| 17 | ENSP00000005257 ENSP00000349467 ENSP00000297494 ENSP00000270202 ENSP00000352121 |
| 22 | ENSP00000005257 ENSP00000342793 ENSP00000284384 ENSP00000254066 ENSP00000268058 ENSP00000269305 ENSP00000353483 ENSP00000250894 ENSP00000352157 |
| 16 | ENSP00000005257 ENSP00000342793 ENSP00000284384 ENSP00000254066 ENSP00000268058 ENSP00000269305 ENSP00000353483 |
| 16 | ENSP00000005257 ENSP00000342793 ENSP00000284384 ENSP00000254066 ENSP00000320940 ENSP00000262367 ENSP00000354394 |
| 16 | ENSP00000005257 ENSP00000342793 ENSP00000284384 ENSP00000254066 ENSP00000268058 ENSP00000269305 ENSP00000355153 |
| 17 | ENSP00000005257 ENSP00000342793 ENSP00000284384 ENSP00000338934 ENSP00000262613 ENSP00000003084 ENSP00000344818 ENSP00000227507 ENSP00000267163 ENSP00000355249 |
| 19 | ENSP00000005257 ENSP00000342793 ENSP00000284384 ENSP00000338934 ENSP00000262613 ENSP00000003084 ENSP00000344818 ENSP00000364133 ENSP00000351905 ENSP00000355896 |
| 15 | ENSP00000005257 ENSP00000342793 ENSP00000284384 ENSP00000338934 ENSP00000262613 ENSP00000003084 ENSP00000344818 ENSP00000358622 |
| 16 | ENSP00000005257 ENSP00000342793 ENSP00000284384 ENSP00000338934 ENSP00000262613 ENSP00000003084 ENSP00000344818 ENSP00000216797 ENSP00000359424 |
| 18 | ENSP00000005257 ENSP00000342793 ENSP00000284384 ENSP00000282561 ENSP00000350941 ENSP00000309845 ENSP00000361120 |
| 16 | ENSP00000005257 ENSP00000342793 ENSP00000284384 ENSP00000338934 ENSP00000262613 ENSP00000003084 ENSP00000344818 ENSP00000338018 ENSP00000361125 |
| 15 | ENSP00000005257 ENSP00000342793 ENSP00000284384 ENSP00000338934 ENSP00000262613 ENSP00000003084 ENSP00000344818 ENSP00000364133 |
| 28 | ENSP00000005257 ENSP00000342793 ENSP00000284384 ENSP00000338934 ENSP00000262613 ENSP00000003084 ENSP00000344818 ENSP00000360266 ENSP00000215832 ENSP00000302486 ENSP00000366244 |
| 18 | ENSP00000005257 ENSP00000349467 ENSP00000297494 ENSP00000270202 ENSP00000366563 |
| 16 | ENSP00000005257 ENSP00000342793 ENSP00000284384 ENSP00000338934 ENSP00000262613 ENSP00000003084 ENSP00000344818 ENSP00000350283 ENSP00000369497 |
| 23 | ENSP00000005257 ENSP00000342793 ENSP00000284384 ENSP00000282561 ENSP00000350941 ENSP00000360683 ENSP00000303830 ENSP00000348986 ENSP00000375892 |
| 15 | ENSP00000005257 ENSP00000342793 ENSP00000284384 ENSP00000338934 ENSP00000262613 ENSP00000003084 ENSP00000344818 ENSP00000384273 |
| 17 | ENSP00000019317 ENSP00000005257 ENSP00000342793 ENSP00000284384 ENSP00000338934 ENSP00000262613 ENSP00000003084 ENSP00000344818 ENSP00000360266 ENSP00000215832 |
| 17 | ENSP00000019317 ENSP00000005257 ENSP00000342793 ENSP00000284384 ENSP00000338934 ENSP00000262613 ENSP00000003084 ENSP00000344818 ENSP00000364133 ENSP00000221930 |
| 19 | ENSP00000019317 ENSP00000005257 ENSP00000342793 ENSP00000298316 ENSP00000348461 ENSP00000278568 ENSP00000288986 ENSP00000261799 ENSP00000222254 |
| 17 | ENSP00000019317 ENSP00000005257 ENSP00000342793 ENSP00000284384 ENSP00000338934 ENSP00000262613 ENSP00000003084 ENSP00000344818 ENSP00000216797 ENSP00000226574 |
| 16 | ENSP00000019317 ENSP00000005257 ENSP00000342793 ENSP00000284384 ENSP00000338934 ENSP00000262613 ENSP00000003084 ENSP00000344818 ENSP00000227507 |
| 17 | ENSP00000019317 ENSP00000005257 ENSP00000342793 ENSP00000284384 ENSP00000338934 ENSP00000262613 ENSP00000003084 ENSP00000344818 ENSP00000364133 ENSP00000238682 |
| 16 | ENSP00000019317 ENSP00000005257 ENSP00000342793 ENSP00000298316 ENSP00000348461 ENSP00000269321 ENSP00000249071 |
| 23 | ENSP00000019317 ENSP00000005257 ENSP00000349467 ENSP00000297494 ENSP00000270202 ENSP00000299421 ENSP00000384515 ENSP00000250617 |
| 17 | ENSP00000019317 ENSP00000005257 ENSP00000349467 ENSP00000297494 ENSP00000335153 ENSP00000251849 |
| 17 | ENSP00000019317 ENSP00000005257 ENSP00000342793 ENSP00000284384 ENSP00000338934 ENSP00000262613 ENSP00000003084 ENSP00000344818 ENSP00000227507 ENSP00000257904 |
| 16 | ENSP00000019317 ENSP00000005257 ENSP00000342793 ENSP00000284384 ENSP00000338934 ENSP00000262613 ENSP00000003084 ENSP00000344818 ENSP00000262160 |
| 19 | ENSP00000019317 ENSP00000005257 ENSP00000349467 ENSP00000297494 ENSP00000270202 ENSP00000263967 ENSP00000262741 |
| 18 | ENSP00000019317 ENSP00000005257 ENSP00000342793 ENSP00000284384 ENSP00000338934 ENSP00000262613 ENSP00000003084 ENSP00000344818 ENSP00000227507 ENSP00000267163 ENSP00000262904 |
| 19 | ENSP00000019317 ENSP00000005257 ENSP00000342793 ENSP00000284384 ENSP00000338934 ENSP00000262613 ENSP00000003084 ENSP00000344818 ENSP00000360266 ENSP00000215832 ENSP00000302486 ENSP00000263025 |
| 52 | ENSP00000019317 ENSP00000005257 ENSP00000349467 ENSP00000297494 ENSP00000270202 ENSP00000219476 ENSP00000263826 |
| 18 | ENSP00000019317 ENSP00000005257 ENSP00000349467 ENSP00000297494 ENSP00000270202 ENSP00000263967 |
| 16 | ENSP00000019317 ENSP00000005257 ENSP00000342793 ENSP00000284384 ENSP00000282561 ENSP00000350941 ENSP00000264657 |
| 17 | ENSP00000019317 ENSP00000005257 ENSP00000342793 ENSP00000284384 ENSP00000338934 ENSP00000262613 ENSP00000003084 ENSP00000344818 ENSP00000275493 ENSP00000265171 |
| 17 | ENSP00000019317 ENSP00000005257 ENSP00000342793 ENSP00000284384 ENSP00000338934 ENSP00000262613 ENSP00000003084 ENSP00000344818 ENSP00000227507 ENSP00000265734 |
| 17 | ENSP00000019317 ENSP00000005257 ENSP00000342793 ENSP00000284384 ENSP00000338934 ENSP00000262613 ENSP00000003084 ENSP00000344818 ENSP00000227507 ENSP00000267163 |
| 17 | ENSP00000019317 ENSP00000005257 ENSP00000342793 ENSP00000284384 ENSP00000254066 ENSP00000268058 ENSP00000269305 ENSP00000267868 |
| 29 | ENSP00000019317 ENSP00000005257 ENSP00000349467 ENSP00000297494 ENSP00000270202 ENSP00000352121 ENSP00000269300 |
| 16 | ENSP00000019317 ENSP00000005257 ENSP00000342793 ENSP00000284384 ENSP00000254066 ENSP00000268058 ENSP00000269305 |
| 16 | ENSP00000019317 ENSP00000005257 ENSP00000342793 ENSP00000284384 ENSP00000338934 ENSP00000262613 ENSP00000003084 ENSP00000344818 ENSP00000269571 |
| 16 | ENSP00000019317 ENSP00000005257 ENSP00000349467 ENSP00000297494 ENSP00000270202 |
| 1 | ENSP00000019317 ENSP00000272519 |
| 17 | ENSP00000019317 ENSP00000005257 ENSP00000342793 ENSP00000284384 ENSP00000338934 ENSP00000262613 ENSP00000003084 ENSP00000344818 ENSP00000264033 ENSP00000274335 |
| 16 | ENSP00000019317 ENSP00000005257 ENSP00000342793 ENSP00000284384 ENSP00000338934 ENSP00000262613 ENSP00000003084 ENSP00000344818 ENSP00000275493 |
| 18 | ENSP00000019317 ENSP00000005257 ENSP00000349467 ENSP00000297494 ENSP00000335153 ENSP00000251849 ENSP00000288602 |
| 18 | ENSP00000019317 ENSP00000005257 ENSP00000349467 ENSP00000297494 ENSP00000270202 ENSP00000289153 |
| 17 | ENSP00000019317 ENSP00000005257 ENSP00000342793 ENSP00000284384 ENSP00000338934 ENSP00000262613 ENSP00000003084 ENSP00000344818 ENSP00000275493 ENSP00000295400 |
| 18 | ENSP00000019317 ENSP00000005257 ENSP00000342793 ENSP00000284384 ENSP00000338934 ENSP00000262613 ENSP00000003084 ENSP00000344818 ENSP00000360266 ENSP00000215832 ENSP00000302486 |
| 18 | ENSP00000019317 ENSP00000005257 ENSP00000342793 ENSP00000284384 ENSP00000338934 ENSP00000262613 ENSP00000003084 ENSP00000344818 ENSP00000358022 ENSP00000293288 ENSP00000302564 |
| 19 | ENSP00000019317 ENSP00000005257 ENSP00000342793 ENSP00000298316 ENSP00000348461 ENSP00000269321 ENSP00000304283 |
| 18 | ENSP00000019317 ENSP00000005257 ENSP00000349467 ENSP00000297494 ENSP00000270202 ENSP00000309103 |
| 16 | ENSP00000019317 ENSP00000005257 ENSP00000342793 ENSP00000298316 ENSP00000348461 ENSP00000268182 ENSP00000314458 |
| 17 | ENSP00000019317 ENSP00000005257 ENSP00000342793 ENSP00000284384 ENSP00000338934 ENSP00000262613 ENSP00000003084 ENSP00000344818 ENSP00000360266 ENSP00000321410 |
| 17 | ENSP00000019317 ENSP00000005257 ENSP00000342793 ENSP00000284384 ENSP00000338934 ENSP00000262613 ENSP00000003084 ENSP00000344818 ENSP00000347858 ENSP00000330237 |
| 16 | ENSP00000019317 ENSP00000005257 ENSP00000342793 ENSP00000284384 ENSP00000338934 ENSP00000262613 ENSP00000003084 ENSP00000344818 ENSP00000332973 |
| 16 | ENSP00000019317 ENSP00000005257 ENSP00000342793 ENSP00000284384 ENSP00000338934 ENSP00000262613 ENSP00000003084 ENSP00000344818 ENSP00000339151 |
| 16 | ENSP00000019317 ENSP00000005257 ENSP00000342793 ENSP00000284384 ENSP00000338934 ENSP00000262613 ENSP00000003084 ENSP00000344818 ENSP00000341551 |
| 3 | ENSP00000019317 ENSP00000005257 ENSP00000342793 |
| 17 | ENSP00000019317 ENSP00000005257 ENSP00000342793 ENSP00000284384 ENSP00000282561 ENSP00000350941 ENSP00000264657 ENSP00000343204 |
| 16 | ENSP00000019317 ENSP00000005257 ENSP00000342793 ENSP00000284384 ENSP00000338934 ENSP00000262613 ENSP00000003084 ENSP00000344818 ENSP00000345571 |
| 14 | ENSP00000019317 ENSP00000005257 ENSP00000342793 ENSP00000298316 ENSP00000348461 |
| 17 | ENSP00000019317 ENSP00000005257 ENSP00000342793 ENSP00000284384 ENSP00000338934 ENSP00000262613 ENSP00000003084 ENSP00000344818 ENSP00000364133 ENSP00000351905 |
| 18 | ENSP00000019317 ENSP00000005257 ENSP00000349467 ENSP00000297494 ENSP00000270202 ENSP00000352121 |
| 23 | ENSP00000019317 ENSP00000005257 ENSP00000342793 ENSP00000284384 ENSP00000254066 ENSP00000268058 ENSP00000269305 ENSP00000353483 ENSP00000250894 ENSP00000352157 |
| 17 | ENSP00000019317 ENSP00000005257 ENSP00000342793 ENSP00000284384 ENSP00000254066 ENSP00000268058 ENSP00000269305 ENSP00000353483 |
| 17 | ENSP00000019317 ENSP00000005257 ENSP00000342793 ENSP00000284384 ENSP00000254066 ENSP00000320940 ENSP00000262367 ENSP00000354394 |
| 17 | ENSP00000019317 ENSP00000005257 ENSP00000342793 ENSP00000284384 ENSP00000254066 ENSP00000268058 ENSP00000269305 ENSP00000355153 |
| 18 | ENSP00000019317 ENSP00000005257 ENSP00000342793 ENSP00000284384 ENSP00000338934 ENSP00000262613 ENSP00000003084 ENSP00000344818 ENSP00000227507 ENSP00000267163 ENSP00000355249 |
| 20 | ENSP00000019317 ENSP00000005257 ENSP00000342793 ENSP00000284384 ENSP00000338934 ENSP00000262613 ENSP00000003084 ENSP00000344818 ENSP00000364133 ENSP00000351905 ENSP00000355896 |
| 16 | ENSP00000019317 ENSP00000005257 ENSP00000342793 ENSP00000284384 ENSP00000338934 ENSP00000262613 ENSP00000003084 ENSP00000344818 ENSP00000358622 |
| 17 | ENSP00000019317 ENSP00000005257 ENSP00000342793 ENSP00000284384 ENSP00000338934 ENSP00000262613 ENSP00000003084 ENSP00000344818 ENSP00000216797 ENSP00000359424 |
| 19 | ENSP00000019317 ENSP00000005257 ENSP00000342793 ENSP00000284384 ENSP00000282561 ENSP00000350941 ENSP00000309845 ENSP00000361120 |
| 17 | ENSP00000019317 ENSP00000005257 ENSP00000342793 ENSP00000284384 ENSP00000338934 ENSP00000262613 ENSP00000003084 ENSP00000344818 ENSP00000338018 ENSP00000361125 |
| 16 | ENSP00000019317 ENSP00000005257 ENSP00000342793 ENSP00000284384 ENSP00000338934 ENSP00000262613 ENSP00000003084 ENSP00000344818 ENSP00000364133 |
| 29 | ENSP00000019317 ENSP00000005257 ENSP00000342793 ENSP00000284384 ENSP00000338934 ENSP00000262613 ENSP00000003084 ENSP00000344818 ENSP00000360266 ENSP00000215832 ENSP00000302486 ENSP00000366244 |
| 19 | ENSP00000019317 ENSP00000005257 ENSP00000349467 ENSP00000297494 ENSP00000270202 ENSP00000366563 |
| 17 | ENSP00000019317 ENSP00000005257 ENSP00000342793 ENSP00000284384 ENSP00000338934 ENSP00000262613 ENSP00000003084 ENSP00000344818 ENSP00000350283 ENSP00000369497 |
| 24 | ENSP00000019317 ENSP00000005257 ENSP00000342793 ENSP00000284384 ENSP00000282561 ENSP00000350941 ENSP00000360683 ENSP00000303830 ENSP00000348986 ENSP00000375892 |
| 16 | ENSP00000019317 ENSP00000005257 ENSP00000342793 ENSP00000284384 ENSP00000338934 ENSP00000262613 ENSP00000003084 ENSP00000344818 ENSP00000384273 |
| 4 | ENSP00000215832 ENSP00000360266 ENSP00000344818 ENSP00000364133 ENSP00000221930 |
| 5 | ENSP00000215832 ENSP00000302486 ENSP00000251849 ENSP00000309845 ENSP00000263967 ENSP00000222254 |
| 4 | ENSP00000215832 ENSP00000360266 ENSP00000263253 ENSP00000226574 |
| 3 | ENSP00000215832 ENSP00000360266 ENSP00000344818 ENSP00000227507 |
| 4 | ENSP00000215832 ENSP00000360266 ENSP00000344818 ENSP00000364133 ENSP00000238682 |
| 7 | ENSP00000215832 ENSP00000360266 ENSP00000344818 ENSP00000270202 ENSP00000348461 ENSP00000269321 ENSP00000249071 |
| 10 | ENSP00000215832 ENSP00000360266 ENSP00000344818 ENSP00000270202 ENSP00000299421 ENSP00000384515 ENSP00000250617 |
| 2 | ENSP00000215832 ENSP00000302486 ENSP00000251849 |
| 4 | ENSP00000215832 ENSP00000360266 ENSP00000344818 ENSP00000227507 ENSP00000257904 |
| 3 | ENSP00000215832 ENSP00000360266 ENSP00000344818 ENSP00000262160 |
| 5 | ENSP00000215832 ENSP00000302486 ENSP00000251849 ENSP00000309845 ENSP00000263967 ENSP00000262741 |
| 5 | ENSP00000215832 ENSP00000302486 ENSP00000251849 ENSP00000267163 ENSP00000262904 |
| 2 | ENSP00000215832 ENSP00000302486 ENSP00000263025 |
| 38 | ENSP00000215832 ENSP00000219476 ENSP00000263826 |
| 4 | ENSP00000215832 ENSP00000302486 ENSP00000251849 ENSP00000309845 ENSP00000263967 |
| 3 | ENSP00000215832 ENSP00000360266 ENSP00000263253 ENSP00000264657 |
| 4 | ENSP00000215832 ENSP00000360266 ENSP00000344818 ENSP00000275493 ENSP00000265171 |
| 4 | ENSP00000215832 ENSP00000360266 ENSP00000344818 ENSP00000227507 ENSP00000265734 |
| 4 | ENSP00000215832 ENSP00000302486 ENSP00000251849 ENSP00000267163 |
| 4 | ENSP00000215832 ENSP00000269305 ENSP00000267868 |
| 16 | ENSP00000215832 ENSP00000360266 ENSP00000344818 ENSP00000270202 ENSP00000352121 ENSP00000269300 |
| 3 | ENSP00000215832 ENSP00000269305 |
| 3 | ENSP00000215832 ENSP00000360266 ENSP00000344818 ENSP00000269571 |
| 3 | ENSP00000215832 ENSP00000360266 ENSP00000344818 ENSP00000270202 |
| 18 | ENSP00000215832 ENSP00000360266 ENSP00000344818 ENSP00000003084 ENSP00000262613 ENSP00000338934 ENSP00000284384 ENSP00000342793 ENSP00000005257 ENSP00000019317 ENSP00000272519 |
| 4 | ENSP00000215832 ENSP00000360266 ENSP00000344818 ENSP00000264033 ENSP00000274335 |
| 3 | ENSP00000215832 ENSP00000360266 ENSP00000344818 ENSP00000275493 |
| 3 | ENSP00000215832 ENSP00000302486 ENSP00000251849 ENSP00000288602 |
| 5 | ENSP00000215832 ENSP00000360266 ENSP00000344818 ENSP00000270202 ENSP00000289153 |
| 4 | ENSP00000215832 ENSP00000360266 ENSP00000344818 ENSP00000275493 ENSP00000295400 |
| 1 | ENSP00000215832 ENSP00000302486 |
| 5 | ENSP00000215832 ENSP00000360266 ENSP00000344818 ENSP00000358022 ENSP00000293288 ENSP00000302564 |
| 10 | ENSP00000215832 ENSP00000360266 ENSP00000344818 ENSP00000264033 ENSP00000302269 ENSP00000304283 |
| 4 | ENSP00000215832 ENSP00000302486 ENSP00000251849 ENSP00000309503 ENSP00000309103 |
| 6 | ENSP00000215832 ENSP00000360266 ENSP00000344818 ENSP00000264033 ENSP00000339007 ENSP00000223023 ENSP00000314458 |
| 2 | ENSP00000215832 ENSP00000360266 ENSP00000321410 |
| 4 | ENSP00000215832 ENSP00000360266 ENSP00000344818 ENSP00000347858 ENSP00000330237 |
| 3 | ENSP00000215832 ENSP00000360266 ENSP00000332973 |
| 3 | ENSP00000215832 ENSP00000360266 ENSP00000344818 ENSP00000339151 |
| 3 | ENSP00000215832 ENSP00000360266 ENSP00000263253 ENSP00000341551 |
| 14 | ENSP00000215832 ENSP00000360266 ENSP00000344818 ENSP00000003084 ENSP00000262613 ENSP00000338934 ENSP00000284384 ENSP00000342793 |
| 4 | ENSP00000215832 ENSP00000360266 ENSP00000263253 ENSP00000264657 ENSP00000343204 |
| 3 | ENSP00000215832 ENSP00000360266 ENSP00000344818 ENSP00000345571 |
| 5 | ENSP00000215832 ENSP00000360266 ENSP00000344818 ENSP00000270202 ENSP00000348461 |
| 4 | ENSP00000215832 ENSP00000360266 ENSP00000344818 ENSP00000364133 ENSP00000351905 |
| 5 | ENSP00000215832 ENSP00000360266 ENSP00000344818 ENSP00000270202 ENSP00000352121 |
| 8 | ENSP00000215832 ENSP00000360266 ENSP00000353483 ENSP00000250894 ENSP00000352157 |
| 2 | ENSP00000215832 ENSP00000360266 ENSP00000353483 |
| 3 | ENSP00000215832 ENSP00000360266 ENSP00000263253 ENSP00000354394 |
| 4 | ENSP00000215832 ENSP00000269305 ENSP00000355153 |
| 5 | ENSP00000215832 ENSP00000302486 ENSP00000251849 ENSP00000267163 ENSP00000355249 |
| 7 | ENSP00000215832 ENSP00000360266 ENSP00000344818 ENSP00000364133 ENSP00000351905 ENSP00000355896 |
| 3 | ENSP00000215832 ENSP00000360266 ENSP00000344818 ENSP00000358622 |
| 4 | ENSP00000215832 ENSP00000360266 ENSP00000344818 ENSP00000216797 ENSP00000359424 |
| 4 | ENSP00000215832 ENSP00000302486 ENSP00000251849 ENSP00000309845 ENSP00000361120 |
| 4 | ENSP00000215832 ENSP00000360266 ENSP00000263253 ENSP00000338018 ENSP00000361125 |
| 3 | ENSP00000215832 ENSP00000360266 ENSP00000344818 ENSP00000364133 |
| 12 | ENSP00000215832 ENSP00000302486 ENSP00000366244 |
| 6 | ENSP00000215832 ENSP00000360266 ENSP00000344818 ENSP00000270202 ENSP00000366563 |
| 4 | ENSP00000215832 ENSP00000360266 ENSP00000344818 ENSP00000350283 ENSP00000369497 |
| 12 | ENSP00000215832 ENSP00000360266 ENSP00000344818 ENSP00000264033 ENSP00000274335 ENSP00000303830 ENSP00000348986 ENSP00000375892 |
| 3 | ENSP00000215832 ENSP00000360266 ENSP00000263253 ENSP00000384273 |
| 6 | ENSP00000221930 ENSP00000364133 ENSP00000344818 ENSP00000264033 ENSP00000244007 ENSP00000261799 ENSP00000222254 |
| 4 | ENSP00000221930 ENSP00000364133 ENSP00000344818 ENSP00000216797 ENSP00000226574 |
| 3 | ENSP00000221930 ENSP00000364133 ENSP00000344818 ENSP00000227507 |
| 2 | ENSP00000221930 ENSP00000351905 ENSP00000238682 |
| 7 | ENSP00000221930 ENSP00000364133 ENSP00000344818 ENSP00000270202 ENSP00000348461 ENSP00000269321 ENSP00000249071 |
| 10 | ENSP00000221930 ENSP00000364133 ENSP00000344818 ENSP00000270202 ENSP00000299421 ENSP00000384515 ENSP00000250617 |
| 5 | ENSP00000221930 ENSP00000364133 ENSP00000344818 ENSP00000206249 ENSP00000335153 ENSP00000251849 |
| 4 | ENSP00000221930 ENSP00000364133 ENSP00000344818 ENSP00000227507 ENSP00000257904 |
| 2 | ENSP00000221930 ENSP00000364133 ENSP00000262160 |
| 6 | ENSP00000221930 ENSP00000364133 ENSP00000344818 ENSP00000270202 ENSP00000263967 ENSP00000262741 |
| 5 | ENSP00000221930 ENSP00000364133 ENSP00000344818 ENSP00000227507 ENSP00000267163 ENSP00000262904 |
| 6 | ENSP00000221930 ENSP00000364133 ENSP00000344818 ENSP00000360266 ENSP00000215832 ENSP00000302486 ENSP00000263025 |
| 39 | ENSP00000221930 ENSP00000364133 ENSP00000344818 ENSP00000270202 ENSP00000219476 ENSP00000263826 |
| 5 | ENSP00000221930 ENSP00000364133 ENSP00000344818 ENSP00000270202 ENSP00000263967 |
| 4 | ENSP00000221930 ENSP00000364133 ENSP00000344818 ENSP00000227507 ENSP00000264657 |
| 4 | ENSP00000221930 ENSP00000364133 ENSP00000344818 ENSP00000275493 ENSP00000265171 |
| 4 | ENSP00000221930 ENSP00000364133 ENSP00000344818 ENSP00000227507 ENSP00000265734 |
| 4 | ENSP00000221930 ENSP00000364133 ENSP00000344818 ENSP00000227507 ENSP00000267163 |
| 4 | ENSP00000221930 ENSP00000364133 ENSP00000344818 ENSP00000269305 ENSP00000267868 |
| 16 | ENSP00000221930 ENSP00000364133 ENSP00000344818 ENSP00000270202 ENSP00000352121 ENSP00000269300 |
| 3 | ENSP00000221930 ENSP00000364133 ENSP00000344818 ENSP00000269305 |
| 3 | ENSP00000221930 ENSP00000364133 ENSP00000344818 ENSP00000269571 |
| 3 | ENSP00000221930 ENSP00000364133 ENSP00000344818 ENSP00000270202 |
| 18 | ENSP00000221930 ENSP00000364133 ENSP00000344818 ENSP00000003084 ENSP00000262613 ENSP00000338934 ENSP00000284384 ENSP00000342793 ENSP00000005257 ENSP00000019317 ENSP00000272519 |
| 4 | ENSP00000221930 ENSP00000364133 ENSP00000344818 ENSP00000264033 ENSP00000274335 |
| 3 | ENSP00000221930 ENSP00000364133 ENSP00000344818 ENSP00000275493 |
| 6 | ENSP00000221930 ENSP00000364133 ENSP00000344818 ENSP00000206249 ENSP00000335153 ENSP00000251849 ENSP00000288602 |
| 5 | ENSP00000221930 ENSP00000364133 ENSP00000344818 ENSP00000270202 ENSP00000289153 |
| 4 | ENSP00000221930 ENSP00000364133 ENSP00000344818 ENSP00000275493 ENSP00000295400 |
| 5 | ENSP00000221930 ENSP00000364133 ENSP00000344818 ENSP00000360266 ENSP00000215832 ENSP00000302486 |
| 5 | ENSP00000221930 ENSP00000364133 ENSP00000344818 ENSP00000358022 ENSP00000293288 ENSP00000302564 |
| 10 | ENSP00000221930 ENSP00000364133 ENSP00000344818 ENSP00000264033 ENSP00000302269 ENSP00000304283 |
| 5 | ENSP00000221930 ENSP00000364133 ENSP00000344818 ENSP00000270202 ENSP00000309103 |
| 6 | ENSP00000221930 ENSP00000364133 ENSP00000344818 ENSP00000264033 ENSP00000339007 ENSP00000223023 ENSP00000314458 |
| 4 | ENSP00000221930 ENSP00000364133 ENSP00000344818 ENSP00000360266 ENSP00000321410 |
| 4 | ENSP00000221930 ENSP00000364133 ENSP00000344818 ENSP00000347858 ENSP00000330237 |
| 3 | ENSP00000221930 ENSP00000364133 ENSP00000344818 ENSP00000332973 |
| 3 | ENSP00000221930 ENSP00000364133 ENSP00000344818 ENSP00000339151 |
| 3 | ENSP00000221930 ENSP00000364133 ENSP00000262160 ENSP00000341551 |
| 14 | ENSP00000221930 ENSP00000364133 ENSP00000344818 ENSP00000003084 ENSP00000262613 ENSP00000338934 ENSP00000284384 ENSP00000342793 |
| 5 | ENSP00000221930 ENSP00000364133 ENSP00000344818 ENSP00000227507 ENSP00000264657 ENSP00000343204 |
| 3 | ENSP00000221930 ENSP00000364133 ENSP00000344818 ENSP00000345571 |
| 5 | ENSP00000221930 ENSP00000364133 ENSP00000344818 ENSP00000270202 ENSP00000348461 |
| 1 | ENSP00000221930 ENSP00000351905 |
| 5 | ENSP00000221930 ENSP00000364133 ENSP00000344818 ENSP00000270202 ENSP00000352121 |
| 10 | ENSP00000221930 ENSP00000364133 ENSP00000344818 ENSP00000269305 ENSP00000353483 ENSP00000250894 ENSP00000352157 |
| 4 | ENSP00000221930 ENSP00000364133 ENSP00000344818 ENSP00000269305 ENSP00000353483 |
| 4 | ENSP00000221930 ENSP00000364133 ENSP00000262160 ENSP00000262367 ENSP00000354394 |
| 4 | ENSP00000221930 ENSP00000364133 ENSP00000344818 ENSP00000269305 ENSP00000355153 |
| 5 | ENSP00000221930 ENSP00000364133 ENSP00000344818 ENSP00000227507 ENSP00000267163 ENSP00000355249 |
| 4 | ENSP00000221930 ENSP00000351905 ENSP00000355896 |
| 3 | ENSP00000221930 ENSP00000364133 ENSP00000344818 ENSP00000358622 |
| 4 | ENSP00000221930 ENSP00000364133 ENSP00000344818 ENSP00000216797 ENSP00000359424 |
| 7 | ENSP00000221930 ENSP00000364133 ENSP00000344818 ENSP00000206249 ENSP00000335153 ENSP00000251849 ENSP00000309845 ENSP00000361120 |
| 4 | ENSP00000221930 ENSP00000364133 ENSP00000344818 ENSP00000338018 ENSP00000361125 |
| 1 | ENSP00000221930 ENSP00000364133 |
| 16 | ENSP00000221930 ENSP00000364133 ENSP00000344818 ENSP00000360266 ENSP00000215832 ENSP00000302486 ENSP00000366244 |
| 6 | ENSP00000221930 ENSP00000364133 ENSP00000344818 ENSP00000270202 ENSP00000366563 |
| 4 | ENSP00000221930 ENSP00000364133 ENSP00000344818 ENSP00000350283 ENSP00000369497 |
| 12 | ENSP00000221930 ENSP00000364133 ENSP00000344818 ENSP00000264033 ENSP00000274335 ENSP00000303830 ENSP00000348986 ENSP00000375892 |
| 3 | ENSP00000221930 ENSP00000364133 ENSP00000344818 ENSP00000384273 |
| 6 | ENSP00000222254 ENSP00000261799 ENSP00000244007 ENSP00000264033 ENSP00000344818 ENSP00000216797 ENSP00000226574 |
| 5 | ENSP00000222254 ENSP00000261799 ENSP00000244007 ENSP00000275493 ENSP00000264657 ENSP00000227507 |
| 6 | ENSP00000222254 ENSP00000261799 ENSP00000244007 ENSP00000264033 ENSP00000344818 ENSP00000364133 ENSP00000238682 |
| 7 | ENSP00000222254 ENSP00000261799 ENSP00000339007 ENSP00000223023 ENSP00000314458 ENSP00000269321 ENSP00000249071 |
| 10 | ENSP00000222254 ENSP00000263967 ENSP00000270202 ENSP00000299421 ENSP00000384515 ENSP00000250617 |
| 3 | ENSP00000222254 ENSP00000263967 ENSP00000309845 ENSP00000251849 |
| 6 | ENSP00000222254 ENSP00000263967 ENSP00000309845 ENSP00000251849 ENSP00000335153 ENSP00000222005 ENSP00000257904 |
| 5 | ENSP00000222254 ENSP00000261799 ENSP00000244007 ENSP00000264033 ENSP00000344818 ENSP00000262160 |
| 2 | ENSP00000222254 ENSP00000263967 ENSP00000262741 |
| 6 | ENSP00000222254 ENSP00000263967 ENSP00000309845 ENSP00000251849 ENSP00000267163 ENSP00000262904 |
| 5 | ENSP00000222254 ENSP00000263967 ENSP00000309845 ENSP00000251849 ENSP00000302486 ENSP00000263025 |
| 39 | ENSP00000222254 ENSP00000263967 ENSP00000270202 ENSP00000219476 ENSP00000263826 |
| 1 | ENSP00000222254 ENSP00000263967 |
| 4 | ENSP00000222254 ENSP00000261799 ENSP00000244007 ENSP00000275493 ENSP00000264657 |
| 4 | ENSP00000222254 ENSP00000261799 ENSP00000244007 ENSP00000275493 ENSP00000265171 |
| 6 | ENSP00000222254 ENSP00000261799 ENSP00000244007 ENSP00000275493 ENSP00000264657 ENSP00000227507 ENSP00000265734 |
| 5 | ENSP00000222254 ENSP00000263967 ENSP00000309845 ENSP00000251849 ENSP00000267163 |
| 6 | ENSP00000222254 ENSP00000263967 ENSP00000309845 ENSP00000251849 ENSP00000335153 ENSP00000269305 ENSP00000267868 |
| 16 | ENSP00000222254 ENSP00000263967 ENSP00000270202 ENSP00000352121 ENSP00000269300 |
| 5 | ENSP00000222254 ENSP00000263967 ENSP00000309845 ENSP00000251849 ENSP00000335153 ENSP00000269305 |
| 4 | ENSP00000222254 ENSP00000261799 ENSP00000274335 ENSP00000267101 ENSP00000269571 |
| 3 | ENSP00000222254 ENSP00000263967 ENSP00000270202 |
| 20 | ENSP00000222254 ENSP00000263967 ENSP00000270202 ENSP00000297494 ENSP00000349467 ENSP00000005257 ENSP00000019317 ENSP00000272519 |
| 2 | ENSP00000222254 ENSP00000261799 ENSP00000274335 |
| 3 | ENSP00000222254 ENSP00000261799 ENSP00000244007 ENSP00000275493 |
| 4 | ENSP00000222254 ENSP00000263967 ENSP00000309845 ENSP00000288602 |
| 5 | ENSP00000222254 ENSP00000263967 ENSP00000270202 ENSP00000289153 |
| 4 | ENSP00000222254 ENSP00000261799 ENSP00000244007 ENSP00000275493 ENSP00000295400 |
| 4 | ENSP00000222254 ENSP00000263967 ENSP00000309845 ENSP00000251849 ENSP00000302486 |
| 6 | ENSP00000222254 ENSP00000263967 ENSP00000270202 ENSP00000309103 ENSP00000302564 |
| 10 | ENSP00000222254 ENSP00000261799 ENSP00000244007 ENSP00000046794 ENSP00000302269 ENSP00000304283 |
| 5 | ENSP00000222254 ENSP00000263967 ENSP00000270202 ENSP00000309103 |
| 5 | ENSP00000222254 ENSP00000261799 ENSP00000339007 ENSP00000223023 ENSP00000314458 |
| 6 | ENSP00000222254 ENSP00000261799 ENSP00000244007 ENSP00000264033 ENSP00000344818 ENSP00000360266 ENSP00000321410 |
| 6 | ENSP00000222254 ENSP00000261799 ENSP00000244007 ENSP00000264033 ENSP00000344818 ENSP00000347858 ENSP00000330237 |
| 5 | ENSP00000222254 ENSP00000261799 ENSP00000244007 ENSP00000264033 ENSP00000344818 ENSP00000332973 |
| 5 | ENSP00000222254 ENSP00000261799 ENSP00000244007 ENSP00000264033 ENSP00000344818 ENSP00000339151 |
| 5 | ENSP00000222254 ENSP00000261799 ENSP00000244007 ENSP00000264033 ENSP00000344818 ENSP00000341551 |
| 16 | ENSP00000222254 ENSP00000263967 ENSP00000270202 ENSP00000348461 ENSP00000298316 ENSP00000342793 |
| 3 | ENSP00000222254 ENSP00000263967 ENSP00000304895 ENSP00000343204 |
| 5 | ENSP00000222254 ENSP00000261799 ENSP00000244007 ENSP00000264033 ENSP00000344818 ENSP00000345571 |
| 5 | ENSP00000222254 ENSP00000263967 ENSP00000270202 ENSP00000348461 |
| 6 | ENSP00000222254 ENSP00000261799 ENSP00000244007 ENSP00000264033 ENSP00000344818 ENSP00000364133 ENSP00000351905 |
| 5 | ENSP00000222254 ENSP00000263967 ENSP00000270202 ENSP00000352121 |
| 10 | ENSP00000222254 ENSP00000263967 ENSP00000304895 ENSP00000353483 ENSP00000250894 ENSP00000352157 |
| 4 | ENSP00000222254 ENSP00000263967 ENSP00000304895 ENSP00000353483 |
| 4 | ENSP00000222254 ENSP00000261799 ENSP00000244007 ENSP00000275493 ENSP00000354394 |
| 5 | ENSP00000222254 ENSP00000263967 ENSP00000270202 ENSP00000417281 ENSP00000355153 |
| 6 | ENSP00000222254 ENSP00000263967 ENSP00000309845 ENSP00000251849 ENSP00000267163 ENSP00000355249 |
| 9 | ENSP00000222254 ENSP00000261799 ENSP00000244007 ENSP00000264033 ENSP00000344818 ENSP00000364133 ENSP00000351905 ENSP00000355896 |
| 5 | ENSP00000222254 ENSP00000261799 ENSP00000244007 ENSP00000264033 ENSP00000344818 ENSP00000358622 |
| 6 | ENSP00000222254 ENSP00000263967 ENSP00000270202 ENSP00000359424 |
| 3 | ENSP00000222254 ENSP00000263967 ENSP00000309845 ENSP00000361120 |
| 6 | ENSP00000222254 ENSP00000263967 ENSP00000309845 ENSP00000251849 ENSP00000335153 ENSP00000338018 ENSP00000361125 |
| 5 | ENSP00000222254 ENSP00000261799 ENSP00000244007 ENSP00000264033 ENSP00000344818 ENSP00000364133 |
| 15 | ENSP00000222254 ENSP00000263967 ENSP00000309845 ENSP00000251849 ENSP00000302486 ENSP00000366244 |
| 6 | ENSP00000222254 ENSP00000263967 ENSP00000270202 ENSP00000366563 |
| 6 | ENSP00000222254 ENSP00000261799 ENSP00000244007 ENSP00000264033 ENSP00000344818 ENSP00000350283 ENSP00000369497 |
| 10 | ENSP00000222254 ENSP00000261799 ENSP00000274335 ENSP00000303830 ENSP00000348986 ENSP00000375892 |
| 5 | ENSP00000222254 ENSP00000261799 ENSP00000244007 ENSP00000264033 ENSP00000344818 ENSP00000384273 |
| 3 | ENSP00000226574 ENSP00000359206 ENSP00000344456 ENSP00000227507 |
| 4 | ENSP00000226574 ENSP00000216797 ENSP00000344818 ENSP00000364133 ENSP00000238682 |
| 7 | ENSP00000226574 ENSP00000216797 ENSP00000344818 ENSP00000270202 ENSP00000348461 ENSP00000269321 ENSP00000249071 |
| 10 | ENSP00000226574 ENSP00000216797 ENSP00000344818 ENSP00000270202 ENSP00000299421 ENSP00000384515 ENSP00000250617 |
| 5 | ENSP00000226574 ENSP00000384273 ENSP00000362649 ENSP00000267163 ENSP00000251849 |
| 4 | ENSP00000226574 ENSP00000359206 ENSP00000344456 ENSP00000227507 ENSP00000257904 |
| 3 | ENSP00000226574 ENSP00000384273 ENSP00000262367 ENSP00000262160 |
| 6 | ENSP00000226574 ENSP00000216797 ENSP00000344818 ENSP00000270202 ENSP00000263967 ENSP00000262741 |
| 4 | ENSP00000226574 ENSP00000384273 ENSP00000362649 ENSP00000267163 ENSP00000262904 |
| 6 | ENSP00000226574 ENSP00000263253 ENSP00000360266 ENSP00000215832 ENSP00000302486 ENSP00000263025 |
| 39 | ENSP00000226574 ENSP00000216797 ENSP00000344818 ENSP00000270202 ENSP00000219476 ENSP00000263826 |
| 5 | ENSP00000226574 ENSP00000216797 ENSP00000344818 ENSP00000270202 ENSP00000263967 |
| 3 | ENSP00000226574 ENSP00000263253 ENSP00000264657 |
| 4 | ENSP00000226574 ENSP00000216797 ENSP00000344818 ENSP00000275493 ENSP00000265171 |
| 4 | ENSP00000226574 ENSP00000359206 ENSP00000344456 ENSP00000227507 ENSP00000265734 |
| 3 | ENSP00000226574 ENSP00000384273 ENSP00000362649 ENSP00000267163 |
| 4 | ENSP00000226574 ENSP00000384273 ENSP00000262367 ENSP00000269305 ENSP00000267868 |
| 16 | ENSP00000226574 ENSP00000216797 ENSP00000344818 ENSP00000270202 ENSP00000352121 ENSP00000269300 |
| 3 | ENSP00000226574 ENSP00000384273 ENSP00000262367 ENSP00000269305 |
| 3 | ENSP00000226574 ENSP00000216797 ENSP00000344818 ENSP00000269571 |
| 3 | ENSP00000226574 ENSP00000216797 ENSP00000344818 ENSP00000270202 |
| 18 | ENSP00000226574 ENSP00000216797 ENSP00000344818 ENSP00000003084 ENSP00000262613 ENSP00000338934 ENSP00000284384 ENSP00000342793 ENSP00000005257 ENSP00000019317 ENSP00000272519 |
| 4 | ENSP00000226574 ENSP00000216797 ENSP00000344818 ENSP00000264033 ENSP00000274335 |
| 3 | ENSP00000226574 ENSP00000216797 ENSP00000344818 ENSP00000275493 |
| 6 | ENSP00000226574 ENSP00000384273 ENSP00000362649 ENSP00000267163 ENSP00000251849 ENSP00000288602 |
| 5 | ENSP00000226574 ENSP00000216797 ENSP00000344818 ENSP00000270202 ENSP00000289153 |
| 4 | ENSP00000226574 ENSP00000216797 ENSP00000344818 ENSP00000275493 ENSP00000295400 |
| 5 | ENSP00000226574 ENSP00000263253 ENSP00000360266 ENSP00000215832 ENSP00000302486 |
| 5 | ENSP00000226574 ENSP00000216797 ENSP00000344818 ENSP00000358022 ENSP00000293288 ENSP00000302564 |
| 10 | ENSP00000226574 ENSP00000216797 ENSP00000344818 ENSP00000264033 ENSP00000302269 ENSP00000304283 |
| 5 | ENSP00000226574 ENSP00000216797 ENSP00000344818 ENSP00000270202 ENSP00000309103 |
| 6 | ENSP00000226574 ENSP00000216797 ENSP00000344818 ENSP00000264033 ENSP00000339007 ENSP00000223023 ENSP00000314458 |
| 4 | ENSP00000226574 ENSP00000263253 ENSP00000360266 ENSP00000321410 |
| 4 | ENSP00000226574 ENSP00000216797 ENSP00000344818 ENSP00000347858 ENSP00000330237 |
| 3 | ENSP00000226574 ENSP00000263253 ENSP00000332973 |
| 2 | ENSP00000226574 ENSP00000216797 ENSP00000339151 |
| 3 | ENSP00000226574 ENSP00000263253 ENSP00000341551 |
| 14 | ENSP00000226574 ENSP00000216797 ENSP00000344818 ENSP00000003084 ENSP00000262613 ENSP00000338934 ENSP00000284384 ENSP00000342793 |
| 4 | ENSP00000226574 ENSP00000263253 ENSP00000264657 ENSP00000343204 |
| 3 | ENSP00000226574 ENSP00000216797 ENSP00000344818 ENSP00000345571 |
| 5 | ENSP00000226574 ENSP00000216797 ENSP00000344818 ENSP00000270202 ENSP00000348461 |
| 4 | ENSP00000226574 ENSP00000216797 ENSP00000344818 ENSP00000364133 ENSP00000351905 |
| 5 | ENSP00000226574 ENSP00000216797 ENSP00000344818 ENSP00000270202 ENSP00000352121 |
| 10 | ENSP00000226574 ENSP00000384273 ENSP00000262367 ENSP00000269305 ENSP00000353483 ENSP00000250894 ENSP00000352157 |
| 4 | ENSP00000226574 ENSP00000384273 ENSP00000262367 ENSP00000269305 ENSP00000353483 |
| 3 | ENSP00000226574 ENSP00000384273 ENSP00000262367 ENSP00000354394 |
| 4 | ENSP00000226574 ENSP00000384273 ENSP00000262367 ENSP00000269305 ENSP00000355153 |
| 4 | ENSP00000226574 ENSP00000384273 ENSP00000362649 ENSP00000267163 ENSP00000355249 |
| 7 | ENSP00000226574 ENSP00000216797 ENSP00000344818 ENSP00000364133 ENSP00000351905 ENSP00000355896 |
| 2 | ENSP00000226574 ENSP00000216797 ENSP00000358622 |
| 2 | ENSP00000226574 ENSP00000216797 ENSP00000359424 |
| 7 | ENSP00000226574 ENSP00000384273 ENSP00000362649 ENSP00000267163 ENSP00000251849 ENSP00000309845 ENSP00000361120 |
| 4 | ENSP00000226574 ENSP00000384273 ENSP00000262367 ENSP00000338018 ENSP00000361125 |
| 3 | ENSP00000226574 ENSP00000216797 ENSP00000344818 ENSP00000364133 |
| 16 | ENSP00000226574 ENSP00000263253 ENSP00000360266 ENSP00000215832 ENSP00000302486 ENSP00000366244 |
| 6 | ENSP00000226574 ENSP00000216797 ENSP00000344818 ENSP00000270202 ENSP00000366563 |
| 4 | ENSP00000226574 ENSP00000216797 ENSP00000344818 ENSP00000350283 ENSP00000369497 |
| 12 | ENSP00000226574 ENSP00000216797 ENSP00000344818 ENSP00000264033 ENSP00000274335 ENSP00000303830 ENSP00000348986 ENSP00000375892 |
| 1 | ENSP00000226574 ENSP00000384273 |
| 3 | ENSP00000227507 ENSP00000344818 ENSP00000364133 ENSP00000238682 |
| 6 | ENSP00000227507 ENSP00000344818 ENSP00000270202 ENSP00000348461 ENSP00000269321 ENSP00000249071 |
| 9 | ENSP00000227507 ENSP00000344818 ENSP00000270202 ENSP00000299421 ENSP00000384515 ENSP00000250617 |
| 3 | ENSP00000227507 ENSP00000267163 ENSP00000251849 |
| 1 | ENSP00000227507 ENSP00000257904 |
| 2 | ENSP00000227507 ENSP00000344818 ENSP00000262160 |
| 5 | ENSP00000227507 ENSP00000344818 ENSP00000270202 ENSP00000263967 ENSP00000262741 |
| 2 | ENSP00000227507 ENSP00000267163 ENSP00000262904 |
| 5 | ENSP00000227507 ENSP00000344818 ENSP00000360266 ENSP00000215832 ENSP00000302486 ENSP00000263025 |
| 38 | ENSP00000227507 ENSP00000344818 ENSP00000270202 ENSP00000219476 ENSP00000263826 |
| 4 | ENSP00000227507 ENSP00000344818 ENSP00000270202 ENSP00000263967 |
| 1 | ENSP00000227507 ENSP00000264657 |
| 3 | ENSP00000227507 ENSP00000264657 ENSP00000275493 ENSP00000265171 |
| 1 | ENSP00000227507 ENSP00000265734 |
| 1 | ENSP00000227507 ENSP00000267163 |
| 3 | ENSP00000227507 ENSP00000244741 ENSP00000269305 ENSP00000267868 |
| 15 | ENSP00000227507 ENSP00000344818 ENSP00000270202 ENSP00000352121 ENSP00000269300 |
| 2 | ENSP00000227507 ENSP00000244741 ENSP00000269305 |
| 2 | ENSP00000227507 ENSP00000344818 ENSP00000269571 |
| 2 | ENSP00000227507 ENSP00000344818 ENSP00000270202 |
| 17 | ENSP00000227507 ENSP00000344818 ENSP00000003084 ENSP00000262613 ENSP00000338934 ENSP00000284384 ENSP00000342793 ENSP00000005257 ENSP00000019317 ENSP00000272519 |
| 3 | ENSP00000227507 ENSP00000344818 ENSP00000264033 ENSP00000274335 |
| 2 | ENSP00000227507 ENSP00000264657 ENSP00000275493 |
| 4 | ENSP00000227507 ENSP00000267163 ENSP00000251849 ENSP00000288602 |
| 4 | ENSP00000227507 ENSP00000344818 ENSP00000270202 ENSP00000289153 |
| 3 | ENSP00000227507 ENSP00000264657 ENSP00000275493 ENSP00000295400 |
| 4 | ENSP00000227507 ENSP00000344818 ENSP00000360266 ENSP00000215832 ENSP00000302486 |
| 4 | ENSP00000227507 ENSP00000344818 ENSP00000358022 ENSP00000293288 ENSP00000302564 |
| 9 | ENSP00000227507 ENSP00000344818 ENSP00000264033 ENSP00000302269 ENSP00000304283 |
| 4 | ENSP00000227507 ENSP00000344818 ENSP00000270202 ENSP00000309103 |
| 5 | ENSP00000227507 ENSP00000344818 ENSP00000264033 ENSP00000339007 ENSP00000223023 ENSP00000314458 |
| 3 | ENSP00000227507 ENSP00000344818 ENSP00000360266 ENSP00000321410 |
| 3 | ENSP00000227507 ENSP00000344818 ENSP00000347858 ENSP00000330237 |
| 2 | ENSP00000227507 ENSP00000344818 ENSP00000332973 |
| 2 | ENSP00000227507 ENSP00000344818 ENSP00000339151 |
| 2 | ENSP00000227507 ENSP00000344818 ENSP00000341551 |
| 13 | ENSP00000227507 ENSP00000344818 ENSP00000003084 ENSP00000262613 ENSP00000338934 ENSP00000284384 ENSP00000342793 |
| 2 | ENSP00000227507 ENSP00000264657 ENSP00000343204 |
| 2 | ENSP00000227507 ENSP00000267163 ENSP00000345571 |
| 4 | ENSP00000227507 ENSP00000344818 ENSP00000270202 ENSP00000348461 |
| 3 | ENSP00000227507 ENSP00000344818 ENSP00000364133 ENSP00000351905 |
| 4 | ENSP00000227507 ENSP00000344818 ENSP00000270202 ENSP00000352121 |
| 9 | ENSP00000227507 ENSP00000244741 ENSP00000269305 ENSP00000353483 ENSP00000250894 ENSP00000352157 |
| 3 | ENSP00000227507 ENSP00000244741 ENSP00000269305 ENSP00000353483 |
| 3 | ENSP00000227507 ENSP00000264657 ENSP00000354394 |
| 2 | ENSP00000227507 ENSP00000257904 ENSP00000355153 |
| 2 | ENSP00000227507 ENSP00000267163 ENSP00000355249 |
| 6 | ENSP00000227507 ENSP00000344818 ENSP00000364133 ENSP00000351905 ENSP00000355896 |
| 2 | ENSP00000227507 ENSP00000344818 ENSP00000358622 |
| 3 | ENSP00000227507 ENSP00000344818 ENSP00000216797 ENSP00000359424 |
| 5 | ENSP00000227507 ENSP00000267163 ENSP00000251849 ENSP00000309845 ENSP00000361120 |
| 3 | ENSP00000227507 ENSP00000344818 ENSP00000338018 ENSP00000361125 |
| 2 | ENSP00000227507 ENSP00000344818 ENSP00000364133 |
| 15 | ENSP00000227507 ENSP00000344818 ENSP00000360266 ENSP00000215832 ENSP00000302486 ENSP00000366244 |
| 5 | ENSP00000227507 ENSP00000344818 ENSP00000270202 ENSP00000366563 |
| 3 | ENSP00000227507 ENSP00000206249 ENSP00000350283 ENSP00000369497 |
| 11 | ENSP00000227507 ENSP00000344818 ENSP00000264033 ENSP00000274335 ENSP00000303830 ENSP00000348986 ENSP00000375892 |
| 2 | ENSP00000227507 ENSP00000344818 ENSP00000384273 |
| 7 | ENSP00000238682 ENSP00000364133 ENSP00000344818 ENSP00000270202 ENSP00000348461 ENSP00000269321 ENSP00000249071 |
| 10 | ENSP00000238682 ENSP00000364133 ENSP00000344818 ENSP00000270202 ENSP00000299421 ENSP00000384515 ENSP00000250617 |
| 5 | ENSP00000238682 ENSP00000364133 ENSP00000344818 ENSP00000206249 ENSP00000335153 ENSP00000251849 |
| 4 | ENSP00000238682 ENSP00000364133 ENSP00000344818 ENSP00000227507 ENSP00000257904 |
| 2 | ENSP00000238682 ENSP00000364133 ENSP00000262160 |
| 6 | ENSP00000238682 ENSP00000364133 ENSP00000344818 ENSP00000270202 ENSP00000263967 ENSP00000262741 |
| 5 | ENSP00000238682 ENSP00000364133 ENSP00000344818 ENSP00000227507 ENSP00000267163 ENSP00000262904 |
| 6 | ENSP00000238682 ENSP00000364133 ENSP00000344818 ENSP00000360266 ENSP00000215832 ENSP00000302486 ENSP00000263025 |
| 39 | ENSP00000238682 ENSP00000364133 ENSP00000344818 ENSP00000270202 ENSP00000219476 ENSP00000263826 |
| 5 | ENSP00000238682 ENSP00000364133 ENSP00000344818 ENSP00000270202 ENSP00000263967 |
| 4 | ENSP00000238682 ENSP00000364133 ENSP00000344818 ENSP00000227507 ENSP00000264657 |
| 4 | ENSP00000238682 ENSP00000364133 ENSP00000344818 ENSP00000275493 ENSP00000265171 |
| 4 | ENSP00000238682 ENSP00000364133 ENSP00000344818 ENSP00000227507 ENSP00000265734 |
| 4 | ENSP00000238682 ENSP00000364133 ENSP00000344818 ENSP00000227507 ENSP00000267163 |
| 4 | ENSP00000238682 ENSP00000364133 ENSP00000344818 ENSP00000269305 ENSP00000267868 |
| 16 | ENSP00000238682 ENSP00000364133 ENSP00000344818 ENSP00000270202 ENSP00000352121 ENSP00000269300 |
| 3 | ENSP00000238682 ENSP00000364133 ENSP00000344818 ENSP00000269305 |
| 3 | ENSP00000238682 ENSP00000364133 ENSP00000344818 ENSP00000269571 |
| 3 | ENSP00000238682 ENSP00000364133 ENSP00000344818 ENSP00000270202 |
| 18 | ENSP00000238682 ENSP00000364133 ENSP00000344818 ENSP00000003084 ENSP00000262613 ENSP00000338934 ENSP00000284384 ENSP00000342793 ENSP00000005257 ENSP00000019317 ENSP00000272519 |
| 4 | ENSP00000238682 ENSP00000364133 ENSP00000344818 ENSP00000264033 ENSP00000274335 |
| 3 | ENSP00000238682 ENSP00000364133 ENSP00000344818 ENSP00000275493 |
| 6 | ENSP00000238682 ENSP00000364133 ENSP00000344818 ENSP00000206249 ENSP00000335153 ENSP00000251849 ENSP00000288602 |
| 5 | ENSP00000238682 ENSP00000364133 ENSP00000344818 ENSP00000270202 ENSP00000289153 |
| 4 | ENSP00000238682 ENSP00000364133 ENSP00000344818 ENSP00000275493 ENSP00000295400 |
| 5 | ENSP00000238682 ENSP00000364133 ENSP00000344818 ENSP00000360266 ENSP00000215832 ENSP00000302486 |
| 5 | ENSP00000238682 ENSP00000364133 ENSP00000344818 ENSP00000358022 ENSP00000293288 ENSP00000302564 |
| 10 | ENSP00000238682 ENSP00000364133 ENSP00000344818 ENSP00000264033 ENSP00000302269 ENSP00000304283 |
| 5 | ENSP00000238682 ENSP00000364133 ENSP00000344818 ENSP00000270202 ENSP00000309103 |
| 6 | ENSP00000238682 ENSP00000364133 ENSP00000344818 ENSP00000264033 ENSP00000339007 ENSP00000223023 ENSP00000314458 |
| 4 | ENSP00000238682 ENSP00000364133 ENSP00000344818 ENSP00000360266 ENSP00000321410 |
| 4 | ENSP00000238682 ENSP00000364133 ENSP00000344818 ENSP00000347858 ENSP00000330237 |
| 3 | ENSP00000238682 ENSP00000364133 ENSP00000344818 ENSP00000332973 |
| 3 | ENSP00000238682 ENSP00000364133 ENSP00000344818 ENSP00000339151 |
| 3 | ENSP00000238682 ENSP00000364133 ENSP00000262160 ENSP00000341551 |
| 14 | ENSP00000238682 ENSP00000364133 ENSP00000344818 ENSP00000003084 ENSP00000262613 ENSP00000338934 ENSP00000284384 ENSP00000342793 |
| 5 | ENSP00000238682 ENSP00000364133 ENSP00000344818 ENSP00000227507 ENSP00000264657 ENSP00000343204 |
| 3 | ENSP00000238682 ENSP00000364133 ENSP00000344818 ENSP00000345571 |
| 5 | ENSP00000238682 ENSP00000364133 ENSP00000344818 ENSP00000270202 ENSP00000348461 |
| 1 | ENSP00000238682 ENSP00000351905 |
| 5 | ENSP00000238682 ENSP00000364133 ENSP00000344818 ENSP00000270202 ENSP00000352121 |
| 10 | ENSP00000238682 ENSP00000364133 ENSP00000344818 ENSP00000269305 ENSP00000353483 ENSP00000250894 ENSP00000352157 |
| 4 | ENSP00000238682 ENSP00000364133 ENSP00000344818 ENSP00000269305 ENSP00000353483 |
| 4 | ENSP00000238682 ENSP00000364133 ENSP00000262160 ENSP00000262367 ENSP00000354394 |
| 4 | ENSP00000238682 ENSP00000364133 ENSP00000344818 ENSP00000269305 ENSP00000355153 |
| 5 | ENSP00000238682 ENSP00000364133 ENSP00000344818 ENSP00000227507 ENSP00000267163 ENSP00000355249 |
| 4 | ENSP00000238682 ENSP00000351905 ENSP00000355896 |
| 3 | ENSP00000238682 ENSP00000364133 ENSP00000344818 ENSP00000358622 |
| 4 | ENSP00000238682 ENSP00000364133 ENSP00000344818 ENSP00000216797 ENSP00000359424 |
| 7 | ENSP00000238682 ENSP00000364133 ENSP00000344818 ENSP00000206249 ENSP00000335153 ENSP00000251849 ENSP00000309845 ENSP00000361120 |
| 4 | ENSP00000238682 ENSP00000364133 ENSP00000344818 ENSP00000338018 ENSP00000361125 |
| 1 | ENSP00000238682 ENSP00000364133 |
| 16 | ENSP00000238682 ENSP00000364133 ENSP00000344818 ENSP00000360266 ENSP00000215832 ENSP00000302486 ENSP00000366244 |
| 6 | ENSP00000238682 ENSP00000364133 ENSP00000344818 ENSP00000270202 ENSP00000366563 |
| 4 | ENSP00000238682 ENSP00000364133 ENSP00000344818 ENSP00000350283 ENSP00000369497 |
| 12 | ENSP00000238682 ENSP00000364133 ENSP00000344818 ENSP00000264033 ENSP00000274335 ENSP00000303830 ENSP00000348986 ENSP00000375892 |
| 3 | ENSP00000238682 ENSP00000364133 ENSP00000344818 ENSP00000384273 |
| 11 | ENSP00000249071 ENSP00000269321 ENSP00000348461 ENSP00000270202 ENSP00000299421 ENSP00000384515 ENSP00000250617 |
| 6 | ENSP00000249071 ENSP00000269321 ENSP00000348461 ENSP00000270202 ENSP00000335153 ENSP00000251849 |
| 7 | ENSP00000249071 ENSP00000269321 ENSP00000348461 ENSP00000270202 ENSP00000335153 ENSP00000222005 ENSP00000257904 |
| 6 | ENSP00000249071 ENSP00000269321 ENSP00000348461 ENSP00000270202 ENSP00000344818 ENSP00000262160 |
| 7 | ENSP00000249071 ENSP00000269321 ENSP00000348461 ENSP00000270202 ENSP00000263967 ENSP00000262741 |
| 7 | ENSP00000249071 ENSP00000269321 ENSP00000348461 ENSP00000270202 ENSP00000417281 ENSP00000267163 ENSP00000262904 |
| 8 | ENSP00000249071 ENSP00000269321 ENSP00000348461 ENSP00000270202 ENSP00000335153 ENSP00000251849 ENSP00000302486 ENSP00000263025 |
| 40 | ENSP00000249071 ENSP00000269321 ENSP00000348461 ENSP00000270202 ENSP00000219476 ENSP00000263826 |
| 6 | ENSP00000249071 ENSP00000269321 ENSP00000348461 ENSP00000270202 ENSP00000263967 |
| 6 | ENSP00000249071 ENSP00000269321 ENSP00000348461 ENSP00000264657 |
| 6 | ENSP00000249071 ENSP00000269321 ENSP00000314458 ENSP00000223023 ENSP00000339007 ENSP00000265171 |
| 7 | ENSP00000249071 ENSP00000269321 ENSP00000348461 ENSP00000270202 ENSP00000344818 ENSP00000227507 ENSP00000265734 |
| 6 | ENSP00000249071 ENSP00000269321 ENSP00000348461 ENSP00000270202 ENSP00000417281 ENSP00000267163 |
| 7 | ENSP00000249071 ENSP00000269321 ENSP00000348461 ENSP00000270202 ENSP00000335153 ENSP00000269305 ENSP00000267868 |
| 17 | ENSP00000249071 ENSP00000269321 ENSP00000348461 ENSP00000270202 ENSP00000352121 ENSP00000269300 |
| 6 | ENSP00000249071 ENSP00000269321 ENSP00000348461 ENSP00000270202 ENSP00000335153 ENSP00000269305 |
| 5 | ENSP00000249071 ENSP00000269321 ENSP00000314458 ENSP00000223023 ENSP00000339007 ENSP00000269571 |
| 4 | ENSP00000249071 ENSP00000269321 ENSP00000348461 ENSP00000270202 |
| 17 | ENSP00000249071 ENSP00000269321 ENSP00000348461 ENSP00000298316 ENSP00000342793 ENSP00000005257 ENSP00000019317 ENSP00000272519 |
| 5 | ENSP00000249071 ENSP00000269321 ENSP00000314458 ENSP00000223023 ENSP00000339007 ENSP00000274335 |
| 5 | ENSP00000249071 ENSP00000269321 ENSP00000314458 ENSP00000223023 ENSP00000339007 ENSP00000275493 |
| 7 | ENSP00000249071 ENSP00000269321 ENSP00000348461 ENSP00000270202 ENSP00000335153 ENSP00000251849 ENSP00000288602 |
| 6 | ENSP00000249071 ENSP00000269321 ENSP00000348461 ENSP00000270202 ENSP00000289153 |
| 6 | ENSP00000249071 ENSP00000269321 ENSP00000314458 ENSP00000223023 ENSP00000339007 ENSP00000275493 ENSP00000295400 |
| 7 | ENSP00000249071 ENSP00000269321 ENSP00000348461 ENSP00000270202 ENSP00000335153 ENSP00000251849 ENSP00000302486 |
| 7 | ENSP00000249071 ENSP00000269321 ENSP00000348461 ENSP00000270202 ENSP00000309103 ENSP00000302564 |
| 5 | ENSP00000249071 ENSP00000269321 ENSP00000304283 |
| 6 | ENSP00000249071 ENSP00000269321 ENSP00000348461 ENSP00000270202 ENSP00000309103 |
| 2 | ENSP00000249071 ENSP00000269321 ENSP00000314458 |
| 7 | ENSP00000249071 ENSP00000269321 ENSP00000348461 ENSP00000270202 ENSP00000344818 ENSP00000360266 ENSP00000321410 |
| 7 | ENSP00000249071 ENSP00000269321 ENSP00000348461 ENSP00000270202 ENSP00000344818 ENSP00000347858 ENSP00000330237 |
| 6 | ENSP00000249071 ENSP00000269321 ENSP00000348461 ENSP00000270202 ENSP00000344818 ENSP00000332973 |
| 6 | ENSP00000249071 ENSP00000269321 ENSP00000348461 ENSP00000270202 ENSP00000344818 ENSP00000339151 |
| 6 | ENSP00000249071 ENSP00000269321 ENSP00000348461 ENSP00000270202 ENSP00000344818 ENSP00000341551 |
| 13 | ENSP00000249071 ENSP00000269321 ENSP00000348461 ENSP00000298316 ENSP00000342793 |
| 6 | ENSP00000249071 ENSP00000269321 ENSP00000314458 ENSP00000223023 ENSP00000339007 ENSP00000304895 ENSP00000343204 |
| 6 | ENSP00000249071 ENSP00000269321 ENSP00000348461 ENSP00000270202 ENSP00000344818 ENSP00000345571 |
| 2 | ENSP00000249071 ENSP00000269321 ENSP00000348461 |
| 7 | ENSP00000249071 ENSP00000269321 ENSP00000348461 ENSP00000270202 ENSP00000344818 ENSP00000364133 ENSP00000351905 |
| 6 | ENSP00000249071 ENSP00000269321 ENSP00000348461 ENSP00000270202 ENSP00000352121 |
| 13 | ENSP00000249071 ENSP00000269321 ENSP00000314458 ENSP00000223023 ENSP00000339007 ENSP00000304895 ENSP00000353483 ENSP00000250894 ENSP00000352157 |
| 7 | ENSP00000249071 ENSP00000269321 ENSP00000314458 ENSP00000223023 ENSP00000339007 ENSP00000304895 ENSP00000353483 |
| 6 | ENSP00000249071 ENSP00000269321 ENSP00000314458 ENSP00000223023 ENSP00000339007 ENSP00000275493 ENSP00000354394 |
| 6 | ENSP00000249071 ENSP00000269321 ENSP00000348461 ENSP00000270202 ENSP00000417281 ENSP00000355153 |
| 7 | ENSP00000249071 ENSP00000269321 ENSP00000348461 ENSP00000270202 ENSP00000417281 ENSP00000267163 ENSP00000355249 |
| 10 | ENSP00000249071 ENSP00000269321 ENSP00000348461 ENSP00000270202 ENSP00000344818 ENSP00000364133 ENSP00000351905 ENSP00000355896 |
| 6 | ENSP00000249071 ENSP00000269321 ENSP00000348461 ENSP00000270202 ENSP00000344818 ENSP00000358622 |
| 7 | ENSP00000249071 ENSP00000269321 ENSP00000348461 ENSP00000270202 ENSP00000359424 |
| 7 | ENSP00000249071 ENSP00000269321 ENSP00000314458 ENSP00000223023 ENSP00000339007 ENSP00000384675 ENSP00000309845 ENSP00000361120 |
| 7 | ENSP00000249071 ENSP00000269321 ENSP00000348461 ENSP00000270202 ENSP00000335153 ENSP00000338018 ENSP00000361125 |
| 6 | ENSP00000249071 ENSP00000269321 ENSP00000348461 ENSP00000270202 ENSP00000344818 ENSP00000364133 |
| 18 | ENSP00000249071 ENSP00000269321 ENSP00000348461 ENSP00000270202 ENSP00000335153 ENSP00000251849 ENSP00000302486 ENSP00000366244 |
| 7 | ENSP00000249071 ENSP00000269321 ENSP00000348461 ENSP00000270202 ENSP00000366563 |
| 7 | ENSP00000249071 ENSP00000269321 ENSP00000348461 ENSP00000270202 ENSP00000344818 ENSP00000350283 ENSP00000369497 |
| 13 | ENSP00000249071 ENSP00000269321 ENSP00000314458 ENSP00000223023 ENSP00000339007 ENSP00000274335 ENSP00000303830 ENSP00000348986 ENSP00000375892 |
| 6 | ENSP00000249071 ENSP00000269321 ENSP00000348461 ENSP00000270202 ENSP00000344818 ENSP00000384273 |
| 9 | ENSP00000250617 ENSP00000384515 ENSP00000299421 ENSP00000270202 ENSP00000335153 ENSP00000251849 |
| 10 | ENSP00000250617 ENSP00000384515 ENSP00000299421 ENSP00000270202 ENSP00000335153 ENSP00000222005 ENSP00000257904 |
| 9 | ENSP00000250617 ENSP00000384515 ENSP00000299421 ENSP00000270202 ENSP00000344818 ENSP00000262160 |
| 10 | ENSP00000250617 ENSP00000384515 ENSP00000299421 ENSP00000270202 ENSP00000263967 ENSP00000262741 |
| 10 | ENSP00000250617 ENSP00000384515 ENSP00000299421 ENSP00000270202 ENSP00000417281 ENSP00000267163 ENSP00000262904 |
| 11 | ENSP00000250617 ENSP00000384515 ENSP00000299421 ENSP00000270202 ENSP00000335153 ENSP00000251849 ENSP00000302486 ENSP00000263025 |
| 43 | ENSP00000250617 ENSP00000384515 ENSP00000299421 ENSP00000270202 ENSP00000219476 ENSP00000263826 |
| 9 | ENSP00000250617 ENSP00000384515 ENSP00000299421 ENSP00000270202 ENSP00000263967 |
| 9 | ENSP00000250617 ENSP00000384515 ENSP00000299421 ENSP00000228307 ENSP00000162330 ENSP00000350941 ENSP00000264657 |
| 10 | ENSP00000250617 ENSP00000384515 ENSP00000299421 ENSP00000228307 ENSP00000341189 ENSP00000339007 ENSP00000265171 |
| 10 | ENSP00000250617 ENSP00000384515 ENSP00000299421 ENSP00000270202 ENSP00000344818 ENSP00000227507 ENSP00000265734 |
| 9 | ENSP00000250617 ENSP00000384515 ENSP00000299421 ENSP00000270202 ENSP00000417281 ENSP00000267163 |
| 10 | ENSP00000250617 ENSP00000384515 ENSP00000299421 ENSP00000270202 ENSP00000335153 ENSP00000269305 ENSP00000267868 |
| 20 | ENSP00000250617 ENSP00000384515 ENSP00000299421 ENSP00000270202 ENSP00000352121 ENSP00000269300 |
| 9 | ENSP00000250617 ENSP00000384515 ENSP00000299421 ENSP00000270202 ENSP00000335153 ENSP00000269305 |
| 9 | ENSP00000250617 ENSP00000384515 ENSP00000299421 ENSP00000270202 ENSP00000335153 ENSP00000269571 |
| 7 | ENSP00000250617 ENSP00000384515 ENSP00000299421 ENSP00000270202 |
| 24 | ENSP00000250617 ENSP00000384515 ENSP00000299421 ENSP00000270202 ENSP00000297494 ENSP00000349467 ENSP00000005257 ENSP00000019317 ENSP00000272519 |
| 9 | ENSP00000250617 ENSP00000384515 ENSP00000299421 ENSP00000228307 ENSP00000300574 ENSP00000274335 |
| 9 | ENSP00000250617 ENSP00000384515 ENSP00000299421 ENSP00000228307 ENSP00000300574 ENSP00000264033 ENSP00000275493 |
| 10 | ENSP00000250617 ENSP00000384515 ENSP00000299421 ENSP00000270202 ENSP00000335153 ENSP00000251849 ENSP00000288602 |
| 9 | ENSP00000250617 ENSP00000384515 ENSP00000299421 ENSP00000270202 ENSP00000289153 |
| 10 | ENSP00000250617 ENSP00000384515 ENSP00000299421 ENSP00000228307 ENSP00000300574 ENSP00000264033 ENSP00000275493 ENSP00000295400 |
| 10 | ENSP00000250617 ENSP00000384515 ENSP00000299421 ENSP00000270202 ENSP00000335153 ENSP00000251849 ENSP00000302486 |
| 10 | ENSP00000250617 ENSP00000384515 ENSP00000299421 ENSP00000270202 ENSP00000309103 ENSP00000302564 |
| 14 | ENSP00000250617 ENSP00000384515 ENSP00000299421 ENSP00000270202 ENSP00000348461 ENSP00000269321 ENSP00000304283 |
| 9 | ENSP00000250617 ENSP00000384515 ENSP00000299421 ENSP00000270202 ENSP00000309103 |
| 10 | ENSP00000250617 ENSP00000384515 ENSP00000299421 ENSP00000228307 ENSP00000341189 ENSP00000339007 ENSP00000223023 ENSP00000314458 |
| 10 | ENSP00000250617 ENSP00000384515 ENSP00000299421 ENSP00000270202 ENSP00000344818 ENSP00000360266 ENSP00000321410 |
| 10 | ENSP00000250617 ENSP00000384515 ENSP00000299421 ENSP00000270202 ENSP00000344818 ENSP00000347858 ENSP00000330237 |
| 9 | ENSP00000250617 ENSP00000384515 ENSP00000299421 ENSP00000270202 ENSP00000344818 ENSP00000332973 |
| 9 | ENSP00000250617 ENSP00000384515 ENSP00000299421 ENSP00000270202 ENSP00000344818 ENSP00000339151 |
| 9 | ENSP00000250617 ENSP00000384515 ENSP00000299421 ENSP00000270202 ENSP00000344818 ENSP00000341551 |
| 20 | ENSP00000250617 ENSP00000384515 ENSP00000299421 ENSP00000270202 ENSP00000348461 ENSP00000298316 ENSP00000342793 |
| 10 | ENSP00000250617 ENSP00000384515 ENSP00000299421 ENSP00000228307 ENSP00000162330 ENSP00000350941 ENSP00000264657 ENSP00000343204 |
| 9 | ENSP00000250617 ENSP00000384515 ENSP00000299421 ENSP00000270202 ENSP00000344818 ENSP00000345571 |
| 9 | ENSP00000250617 ENSP00000384515 ENSP00000299421 ENSP00000270202 ENSP00000348461 |
| 10 | ENSP00000250617 ENSP00000384515 ENSP00000299421 ENSP00000270202 ENSP00000344818 ENSP00000364133 ENSP00000351905 |
| 9 | ENSP00000250617 ENSP00000384515 ENSP00000299421 ENSP00000270202 ENSP00000352121 |
| 16 | ENSP00000250617 ENSP00000384515 ENSP00000299421 ENSP00000270202 ENSP00000335153 ENSP00000269305 ENSP00000353483 ENSP00000250894 ENSP00000352157 |
| 10 | ENSP00000250617 ENSP00000384515 ENSP00000299421 ENSP00000270202 ENSP00000335153 ENSP00000269305 ENSP00000353483 |
| 10 | ENSP00000250617 ENSP00000384515 ENSP00000299421 ENSP00000228307 ENSP00000300574 ENSP00000264033 ENSP00000275493 ENSP00000354394 |
| 9 | ENSP00000250617 ENSP00000384515 ENSP00000299421 ENSP00000270202 ENSP00000417281 ENSP00000355153 |
| 10 | ENSP00000250617 ENSP00000384515 ENSP00000299421 ENSP00000270202 ENSP00000417281 ENSP00000267163 ENSP00000355249 |
| 13 | ENSP00000250617 ENSP00000384515 ENSP00000299421 ENSP00000270202 ENSP00000344818 ENSP00000364133 ENSP00000351905 ENSP00000355896 |
| 9 | ENSP00000250617 ENSP00000384515 ENSP00000299421 ENSP00000270202 ENSP00000344818 ENSP00000358622 |
| 10 | ENSP00000250617 ENSP00000384515 ENSP00000299421 ENSP00000270202 ENSP00000359424 |
| 11 | ENSP00000250617 ENSP00000384515 ENSP00000299421 ENSP00000270202 ENSP00000335153 ENSP00000251849 ENSP00000309845 ENSP00000361120 |
| 10 | ENSP00000250617 ENSP00000384515 ENSP00000299421 ENSP00000270202 ENSP00000335153 ENSP00000338018 ENSP00000361125 |
| 9 | ENSP00000250617 ENSP00000384515 ENSP00000299421 ENSP00000270202 ENSP00000344818 ENSP00000364133 |
| 21 | ENSP00000250617 ENSP00000384515 ENSP00000299421 ENSP00000270202 ENSP00000335153 ENSP00000251849 ENSP00000302486 ENSP00000366244 |
| 10 | ENSP00000250617 ENSP00000384515 ENSP00000299421 ENSP00000270202 ENSP00000366563 |
| 10 | ENSP00000250617 ENSP00000384515 ENSP00000299421 ENSP00000270202 ENSP00000344818 ENSP00000350283 ENSP00000369497 |
| 16 | ENSP00000250617 ENSP00000384515 ENSP00000299421 ENSP00000228307 ENSP00000162330 ENSP00000360683 ENSP00000303830 ENSP00000348986 ENSP00000375892 |
| 9 | ENSP00000250617 ENSP00000384515 ENSP00000299421 ENSP00000270202 ENSP00000344818 ENSP00000384273 |
| 3 | ENSP00000251849 ENSP00000335153 ENSP00000222005 ENSP00000257904 |
| 4 | ENSP00000251849 ENSP00000335153 ENSP00000206249 ENSP00000262367 ENSP00000262160 |
| 3 | ENSP00000251849 ENSP00000309845 ENSP00000263967 ENSP00000262741 |
| 3 | ENSP00000251849 ENSP00000267163 ENSP00000262904 |
| 2 | ENSP00000251849 ENSP00000302486 ENSP00000263025 |
| 37 | ENSP00000251849 ENSP00000300161 ENSP00000219476 ENSP00000263826 |
| 2 | ENSP00000251849 ENSP00000309845 ENSP00000263967 |
| 4 | ENSP00000251849 ENSP00000335153 ENSP00000206249 ENSP00000227507 ENSP00000264657 |
| 4 | ENSP00000251849 ENSP00000335153 ENSP00000269571 ENSP00000265171 |
| 3 | ENSP00000251849 ENSP00000267163 ENSP00000265734 |
| 2 | ENSP00000251849 ENSP00000267163 |
| 3 | ENSP00000251849 ENSP00000335153 ENSP00000269305 ENSP00000267868 |
| 15 | ENSP00000251849 ENSP00000335153 ENSP00000270202 ENSP00000352121 ENSP00000269300 |
| 2 | ENSP00000251849 ENSP00000335153 ENSP00000269305 |
| 2 | ENSP00000251849 ENSP00000335153 ENSP00000269571 |
| 2 | ENSP00000251849 ENSP00000335153 ENSP00000270202 |
| 18 | ENSP00000251849 ENSP00000335153 ENSP00000297494 ENSP00000349467 ENSP00000005257 ENSP00000019317 ENSP00000272519 |
| 3 | ENSP00000251849 ENSP00000309845 ENSP00000263967 ENSP00000274335 |
| 3 | ENSP00000251849 ENSP00000335153 ENSP00000269571 ENSP00000275493 |
| 1 | ENSP00000251849 ENSP00000288602 |
| 4 | ENSP00000251849 ENSP00000335153 ENSP00000270202 ENSP00000289153 |
| 4 | ENSP00000251849 ENSP00000335153 ENSP00000269571 ENSP00000275493 ENSP00000295400 |
| 1 | ENSP00000251849 ENSP00000302486 |
| 3 | ENSP00000251849 ENSP00000309503 ENSP00000309103 ENSP00000302564 |
| 9 | ENSP00000251849 ENSP00000335153 ENSP00000270202 ENSP00000348461 ENSP00000269321 ENSP00000304283 |
| 2 | ENSP00000251849 ENSP00000309503 ENSP00000309103 |
| 5 | ENSP00000251849 ENSP00000335153 ENSP00000269571 ENSP00000339007 ENSP00000223023 ENSP00000314458 |
| 4 | ENSP00000251849 ENSP00000335153 ENSP00000269305 ENSP00000321410 |
| 5 | ENSP00000251849 ENSP00000335153 ENSP00000206249 ENSP00000344818 ENSP00000347858 ENSP00000330237 |
| 4 | ENSP00000251849 ENSP00000335153 ENSP00000206249 ENSP00000263253 ENSP00000332973 |
| 4 | ENSP00000251849 ENSP00000335153 ENSP00000206249 ENSP00000344818 ENSP00000339151 |
| 4 | ENSP00000251849 ENSP00000335153 ENSP00000206249 ENSP00000263253 ENSP00000341551 |
| 15 | ENSP00000251849 ENSP00000335153 ENSP00000270202 ENSP00000348461 ENSP00000298316 ENSP00000342793 |
| 4 | ENSP00000251849 ENSP00000309845 ENSP00000263967 ENSP00000304895 ENSP00000343204 |
| 3 | ENSP00000251849 ENSP00000267163 ENSP00000345571 |
| 4 | ENSP00000251849 ENSP00000335153 ENSP00000270202 ENSP00000348461 |
| 5 | ENSP00000251849 ENSP00000335153 ENSP00000206249 ENSP00000344818 ENSP00000364133 ENSP00000351905 |
| 4 | ENSP00000251849 ENSP00000335153 ENSP00000270202 ENSP00000352121 |
| 9 | ENSP00000251849 ENSP00000335153 ENSP00000269305 ENSP00000353483 ENSP00000250894 ENSP00000352157 |
| 3 | ENSP00000251849 ENSP00000335153 ENSP00000269305 ENSP00000353483 |
| 4 | ENSP00000251849 ENSP00000335153 ENSP00000206249 ENSP00000262367 ENSP00000354394 |
| 3 | ENSP00000251849 ENSP00000335153 ENSP00000269305 ENSP00000355153 |
| 3 | ENSP00000251849 ENSP00000267163 ENSP00000355249 |
| 8 | ENSP00000251849 ENSP00000335153 ENSP00000206249 ENSP00000344818 ENSP00000364133 ENSP00000351905 ENSP00000355896 |
| 4 | ENSP00000251849 ENSP00000335153 ENSP00000206249 ENSP00000344818 ENSP00000358622 |
| 5 | ENSP00000251849 ENSP00000335153 ENSP00000270202 ENSP00000359424 |
| 2 | ENSP00000251849 ENSP00000309845 ENSP00000361120 |
| 3 | ENSP00000251849 ENSP00000335153 ENSP00000338018 ENSP00000361125 |
| 4 | ENSP00000251849 ENSP00000335153 ENSP00000206249 ENSP00000344818 ENSP00000364133 |
| 12 | ENSP00000251849 ENSP00000302486 ENSP00000366244 |
| 5 | ENSP00000251849 ENSP00000335153 ENSP00000270202 ENSP00000366563 |
| 4 | ENSP00000251849 ENSP00000335153 ENSP00000269305 ENSP00000267868 ENSP00000369497 |
| 11 | ENSP00000251849 ENSP00000309845 ENSP00000263967 ENSP00000274335 ENSP00000303830 ENSP00000348986 ENSP00000375892 |
| 4 | ENSP00000251849 ENSP00000335153 ENSP00000206249 ENSP00000262367 ENSP00000384273 |
| 3 | ENSP00000257904 ENSP00000227507 ENSP00000344818 ENSP00000262160 |
| 6 | ENSP00000257904 ENSP00000228872 ENSP00000270202 ENSP00000263967 ENSP00000262741 |
| 2 | ENSP00000257904 ENSP00000267163 ENSP00000262904 |
| 5 | ENSP00000257904 ENSP00000267163 ENSP00000251849 ENSP00000302486 ENSP00000263025 |
| 39 | ENSP00000257904 ENSP00000228872 ENSP00000270202 ENSP00000219476 ENSP00000263826 |
| 5 | ENSP00000257904 ENSP00000228872 ENSP00000270202 ENSP00000263967 |
| 2 | ENSP00000257904 ENSP00000227507 ENSP00000264657 |
| 4 | ENSP00000257904 ENSP00000227507 ENSP00000264657 ENSP00000275493 ENSP00000265171 |
| 2 | ENSP00000257904 ENSP00000227507 ENSP00000265734 |
| 1 | ENSP00000257904 ENSP00000267163 |
| 3 | ENSP00000257904 ENSP00000244741 ENSP00000269305 ENSP00000267868 |
| 16 | ENSP00000257904 ENSP00000228872 ENSP00000270202 ENSP00000352121 ENSP00000269300 |
| 2 | ENSP00000257904 ENSP00000244741 ENSP00000269305 |
| 3 | ENSP00000257904 ENSP00000222005 ENSP00000335153 ENSP00000269571 |
| 3 | ENSP00000257904 ENSP00000228872 ENSP00000270202 |
| 18 | ENSP00000257904 ENSP00000227507 ENSP00000344818 ENSP00000003084 ENSP00000262613 ENSP00000338934 ENSP00000284384 ENSP00000342793 ENSP00000005257 ENSP00000019317 ENSP00000272519 |
| 4 | ENSP00000257904 ENSP00000227507 ENSP00000344818 ENSP00000264033 ENSP00000274335 |
| 3 | ENSP00000257904 ENSP00000227507 ENSP00000264657 ENSP00000275493 |
| 4 | ENSP00000257904 ENSP00000267163 ENSP00000251849 ENSP00000288602 |
| 5 | ENSP00000257904 ENSP00000228872 ENSP00000270202 ENSP00000289153 |
| 4 | ENSP00000257904 ENSP00000227507 ENSP00000264657 ENSP00000275493 ENSP00000295400 |
| 4 | ENSP00000257904 ENSP00000267163 ENSP00000251849 ENSP00000302486 |
| 5 | ENSP00000257904 ENSP00000244741 ENSP00000269305 ENSP00000302564 |
| 10 | ENSP00000257904 ENSP00000227507 ENSP00000344818 ENSP00000264033 ENSP00000302269 ENSP00000304283 |
| 4 | ENSP00000257904 ENSP00000244741 ENSP00000269305 ENSP00000329623 ENSP00000309103 |
| 6 | ENSP00000257904 ENSP00000227507 ENSP00000344818 ENSP00000264033 ENSP00000339007 ENSP00000223023 ENSP00000314458 |
| 4 | ENSP00000257904 ENSP00000244741 ENSP00000269305 ENSP00000321410 |
| 4 | ENSP00000257904 ENSP00000227507 ENSP00000344818 ENSP00000347858 ENSP00000330237 |
| 3 | ENSP00000257904 ENSP00000227507 ENSP00000344818 ENSP00000332973 |
| 3 | ENSP00000257904 ENSP00000227507 ENSP00000344818 ENSP00000339151 |
| 3 | ENSP00000257904 ENSP00000227507 ENSP00000344818 ENSP00000341551 |
| 14 | ENSP00000257904 ENSP00000227507 ENSP00000344818 ENSP00000003084 ENSP00000262613 ENSP00000338934 ENSP00000284384 ENSP00000342793 |
| 3 | ENSP00000257904 ENSP00000227507 ENSP00000264657 ENSP00000343204 |
| 2 | ENSP00000257904 ENSP00000267163 ENSP00000345571 |
| 5 | ENSP00000257904 ENSP00000228872 ENSP00000270202 ENSP00000348461 |
| 4 | ENSP00000257904 ENSP00000227507 ENSP00000344818 ENSP00000364133 ENSP00000351905 |
| 5 | ENSP00000257904 ENSP00000228872 ENSP00000270202 ENSP00000352121 |
| 9 | ENSP00000257904 ENSP00000244741 ENSP00000269305 ENSP00000353483 ENSP00000250894 ENSP00000352157 |
| 3 | ENSP00000257904 ENSP00000244741 ENSP00000269305 ENSP00000353483 |
| 4 | ENSP00000257904 ENSP00000227507 ENSP00000264657 ENSP00000354394 |
| 1 | ENSP00000257904 ENSP00000355153 |
| 2 | ENSP00000257904 ENSP00000267163 ENSP00000355249 |
| 7 | ENSP00000257904 ENSP00000227507 ENSP00000344818 ENSP00000364133 ENSP00000351905 ENSP00000355896 |
| 3 | ENSP00000257904 ENSP00000227507 ENSP00000344818 ENSP00000358622 |
| 4 | ENSP00000257904 ENSP00000227507 ENSP00000344818 ENSP00000216797 ENSP00000359424 |
| 5 | ENSP00000257904 ENSP00000267163 ENSP00000251849 ENSP00000309845 ENSP00000361120 |
| 4 | ENSP00000257904 ENSP00000244741 ENSP00000269305 ENSP00000338018 ENSP00000361125 |
| 3 | ENSP00000257904 ENSP00000227507 ENSP00000344818 ENSP00000364133 |
| 15 | ENSP00000257904 ENSP00000267163 ENSP00000251849 ENSP00000302486 ENSP00000366244 |
| 6 | ENSP00000257904 ENSP00000228872 ENSP00000270202 ENSP00000366563 |
| 4 | ENSP00000257904 ENSP00000244741 ENSP00000269305 ENSP00000267868 ENSP00000369497 |
| 12 | ENSP00000257904 ENSP00000227507 ENSP00000344818 ENSP00000264033 ENSP00000274335 ENSP00000303830 ENSP00000348986 ENSP00000375892 |
| 3 | ENSP00000257904 ENSP00000227507 ENSP00000344818 ENSP00000384273 |
| 5 | ENSP00000262160 ENSP00000344818 ENSP00000270202 ENSP00000263967 ENSP00000262741 |
| 4 | ENSP00000262160 ENSP00000344818 ENSP00000227507 ENSP00000267163 ENSP00000262904 |
| 5 | ENSP00000262160 ENSP00000344818 ENSP00000360266 ENSP00000215832 ENSP00000302486 ENSP00000263025 |
| 38 | ENSP00000262160 ENSP00000344818 ENSP00000270202 ENSP00000219476 ENSP00000263826 |
| 4 | ENSP00000262160 ENSP00000344818 ENSP00000270202 ENSP00000263967 |
| 3 | ENSP00000262160 ENSP00000344818 ENSP00000227507 ENSP00000264657 |
| 3 | ENSP00000262160 ENSP00000344818 ENSP00000275493 ENSP00000265171 |
| 3 | ENSP00000262160 ENSP00000344818 ENSP00000227507 ENSP00000265734 |
| 3 | ENSP00000262160 ENSP00000344818 ENSP00000227507 ENSP00000267163 |
| 3 | ENSP00000262160 ENSP00000262367 ENSP00000269305 ENSP00000267868 |
| 15 | ENSP00000262160 ENSP00000344818 ENSP00000270202 ENSP00000352121 ENSP00000269300 |
| 2 | ENSP00000262160 ENSP00000262367 ENSP00000269305 |
| 2 | ENSP00000262160 ENSP00000344818 ENSP00000269571 |
| 2 | ENSP00000262160 ENSP00000344818 ENSP00000270202 |
| 17 | ENSP00000262160 ENSP00000344818 ENSP00000003084 ENSP00000262613 ENSP00000338934 ENSP00000284384 ENSP00000342793 ENSP00000005257 ENSP00000019317 ENSP00000272519 |
| 3 | ENSP00000262160 ENSP00000344818 ENSP00000264033 ENSP00000274335 |
| 2 | ENSP00000262160 ENSP00000344818 ENSP00000275493 |
| 5 | ENSP00000262160 ENSP00000262367 ENSP00000206249 ENSP00000335153 ENSP00000251849 ENSP00000288602 |
| 4 | ENSP00000262160 ENSP00000344818 ENSP00000270202 ENSP00000289153 |
| 3 | ENSP00000262160 ENSP00000344818 ENSP00000275493 ENSP00000295400 |
| 4 | ENSP00000262160 ENSP00000344818 ENSP00000360266 ENSP00000215832 ENSP00000302486 |
| 4 | ENSP00000262160 ENSP00000344818 ENSP00000358022 ENSP00000293288 ENSP00000302564 |
| 9 | ENSP00000262160 ENSP00000344818 ENSP00000264033 ENSP00000302269 ENSP00000304283 |
| 4 | ENSP00000262160 ENSP00000344818 ENSP00000270202 ENSP00000309103 |
| 5 | ENSP00000262160 ENSP00000344818 ENSP00000264033 ENSP00000339007 ENSP00000223023 ENSP00000314458 |
| 3 | ENSP00000262160 ENSP00000344818 ENSP00000360266 ENSP00000321410 |
| 3 | ENSP00000262160 ENSP00000344818 ENSP00000347858 ENSP00000330237 |
| 2 | ENSP00000262160 ENSP00000262435 ENSP00000332973 |
| 2 | ENSP00000262160 ENSP00000344818 ENSP00000339151 |
| 1 | ENSP00000262160 ENSP00000341551 |
| 13 | ENSP00000262160 ENSP00000344818 ENSP00000003084 ENSP00000262613 ENSP00000338934 ENSP00000284384 ENSP00000342793 |
| 4 | ENSP00000262160 ENSP00000262367 ENSP00000354394 ENSP00000343204 |
| 2 | ENSP00000262160 ENSP00000329357 ENSP00000345571 |
| 4 | ENSP00000262160 ENSP00000344818 ENSP00000270202 ENSP00000348461 |
| 2 | ENSP00000262160 ENSP00000364133 ENSP00000351905 |
| 4 | ENSP00000262160 ENSP00000344818 ENSP00000270202 ENSP00000352121 |
| 9 | ENSP00000262160 ENSP00000262367 ENSP00000269305 ENSP00000353483 ENSP00000250894 ENSP00000352157 |
| 3 | ENSP00000262160 ENSP00000262367 ENSP00000269305 ENSP00000353483 |
| 2 | ENSP00000262160 ENSP00000262367 ENSP00000354394 |
| 3 | ENSP00000262160 ENSP00000262367 ENSP00000269305 ENSP00000355153 |
| 4 | ENSP00000262160 ENSP00000344818 ENSP00000227507 ENSP00000267163 ENSP00000355249 |
| 5 | ENSP00000262160 ENSP00000364133 ENSP00000351905 ENSP00000355896 |
| 2 | ENSP00000262160 ENSP00000344818 ENSP00000358622 |
| 3 | ENSP00000262160 ENSP00000344818 ENSP00000216797 ENSP00000359424 |
| 6 | ENSP00000262160 ENSP00000262367 ENSP00000206249 ENSP00000335153 ENSP00000251849 ENSP00000309845 ENSP00000361120 |
| 3 | ENSP00000262160 ENSP00000262367 ENSP00000338018 ENSP00000361125 |
| 1 | ENSP00000262160 ENSP00000364133 |
| 15 | ENSP00000262160 ENSP00000344818 ENSP00000360266 ENSP00000215832 ENSP00000302486 ENSP00000366244 |
| 5 | ENSP00000262160 ENSP00000344818 ENSP00000270202 ENSP00000366563 |
| 3 | ENSP00000262160 ENSP00000344818 ENSP00000350283 ENSP00000369497 |
| 11 | ENSP00000262160 ENSP00000344818 ENSP00000264033 ENSP00000274335 ENSP00000303830 ENSP00000348986 ENSP00000375892 |
| 2 | ENSP00000262160 ENSP00000262367 ENSP00000384273 |
| 6 | ENSP00000262741 ENSP00000263967 ENSP00000309845 ENSP00000251849 ENSP00000267163 ENSP00000262904 |
| 5 | ENSP00000262741 ENSP00000263967 ENSP00000309845 ENSP00000251849 ENSP00000302486 ENSP00000263025 |
| 39 | ENSP00000262741 ENSP00000263967 ENSP00000270202 ENSP00000219476 ENSP00000263826 |
| 1 | ENSP00000262741 ENSP00000263967 |
| 4 | ENSP00000262741 ENSP00000263967 ENSP00000304895 ENSP00000343204 ENSP00000264657 |
| 5 | ENSP00000262741 ENSP00000263967 ENSP00000274335 ENSP00000267101 ENSP00000265171 |
| 6 | ENSP00000262741 ENSP00000263967 ENSP00000304895 ENSP00000343204 ENSP00000264657 ENSP00000227507 ENSP00000265734 |
| 5 | ENSP00000262741 ENSP00000263967 ENSP00000309845 ENSP00000251849 ENSP00000267163 |
| 6 | ENSP00000262741 ENSP00000263967 ENSP00000309845 ENSP00000251849 ENSP00000335153 ENSP00000269305 ENSP00000267868 |
| 16 | ENSP00000262741 ENSP00000263967 ENSP00000270202 ENSP00000352121 ENSP00000269300 |
| 5 | ENSP00000262741 ENSP00000263967 ENSP00000309845 ENSP00000251849 ENSP00000335153 ENSP00000269305 |
| 4 | ENSP00000262741 ENSP00000263967 ENSP00000274335 ENSP00000267101 ENSP00000269571 |
| 3 | ENSP00000262741 ENSP00000263967 ENSP00000270202 |
| 20 | ENSP00000262741 ENSP00000263967 ENSP00000270202 ENSP00000297494 ENSP00000349467 ENSP00000005257 ENSP00000019317 ENSP00000272519 |
| 2 | ENSP00000262741 ENSP00000263967 ENSP00000274335 |
| 4 | ENSP00000262741 ENSP00000263967 ENSP00000274335 ENSP00000264033 ENSP00000275493 |
| 4 | ENSP00000262741 ENSP00000263967 ENSP00000309845 ENSP00000288602 |
| 5 | ENSP00000262741 ENSP00000263967 ENSP00000270202 ENSP00000289153 |
| 5 | ENSP00000262741 ENSP00000263967 ENSP00000274335 ENSP00000264033 ENSP00000275493 ENSP00000295400 |
| 4 | ENSP00000262741 ENSP00000263967 ENSP00000309845 ENSP00000251849 ENSP00000302486 |
| 6 | ENSP00000262741 ENSP00000263967 ENSP00000270202 ENSP00000309103 ENSP00000302564 |
| 10 | ENSP00000262741 ENSP00000263967 ENSP00000274335 ENSP00000264033 ENSP00000302269 ENSP00000304283 |
| 5 | ENSP00000262741 ENSP00000263967 ENSP00000270202 ENSP00000309103 |
| 5 | ENSP00000262741 ENSP00000263967 ENSP00000274335 ENSP00000339007 ENSP00000223023 ENSP00000314458 |
| 6 | ENSP00000262741 ENSP00000263967 ENSP00000274335 ENSP00000264033 ENSP00000344818 ENSP00000360266 ENSP00000321410 |
| 6 | ENSP00000262741 ENSP00000263967 ENSP00000274335 ENSP00000264033 ENSP00000344818 ENSP00000347858 ENSP00000330237 |
| 5 | ENSP00000262741 ENSP00000263967 ENSP00000274335 ENSP00000264033 ENSP00000344818 ENSP00000332973 |
| 5 | ENSP00000262741 ENSP00000263967 ENSP00000274335 ENSP00000264033 ENSP00000344818 ENSP00000339151 |
| 5 | ENSP00000262741 ENSP00000263967 ENSP00000274335 ENSP00000264033 ENSP00000344818 ENSP00000341551 |
| 16 | ENSP00000262741 ENSP00000263967 ENSP00000270202 ENSP00000348461 ENSP00000298316 ENSP00000342793 |
| 3 | ENSP00000262741 ENSP00000263967 ENSP00000304895 ENSP00000343204 |
| 5 | ENSP00000262741 ENSP00000263967 ENSP00000274335 ENSP00000264033 ENSP00000344818 ENSP00000345571 |
| 5 | ENSP00000262741 ENSP00000263967 ENSP00000270202 ENSP00000348461 |
| 6 | ENSP00000262741 ENSP00000263967 ENSP00000274335 ENSP00000264033 ENSP00000344818 ENSP00000364133 ENSP00000351905 |
| 5 | ENSP00000262741 ENSP00000263967 ENSP00000270202 ENSP00000352121 |
| 10 | ENSP00000262741 ENSP00000263967 ENSP00000304895 ENSP00000353483 ENSP00000250894 ENSP00000352157 |
| 4 | ENSP00000262741 ENSP00000263967 ENSP00000304895 ENSP00000353483 |
| 4 | ENSP00000262741 ENSP00000263967 ENSP00000304895 ENSP00000371067 ENSP00000354394 |
| 5 | ENSP00000262741 ENSP00000263967 ENSP00000270202 ENSP00000417281 ENSP00000355153 |
| 6 | ENSP00000262741 ENSP00000263967 ENSP00000309845 ENSP00000251849 ENSP00000267163 ENSP00000355249 |
| 9 | ENSP00000262741 ENSP00000263967 ENSP00000274335 ENSP00000264033 ENSP00000344818 ENSP00000364133 ENSP00000351905 ENSP00000355896 |
| 5 | ENSP00000262741 ENSP00000263967 ENSP00000274335 ENSP00000264033 ENSP00000344818 ENSP00000358622 |
| 6 | ENSP00000262741 ENSP00000263967 ENSP00000270202 ENSP00000359424 |
| 3 | ENSP00000262741 ENSP00000263967 ENSP00000309845 ENSP00000361120 |
| 6 | ENSP00000262741 ENSP00000263967 ENSP00000309845 ENSP00000251849 ENSP00000335153 ENSP00000338018 ENSP00000361125 |
| 5 | ENSP00000262741 ENSP00000263967 ENSP00000274335 ENSP00000264033 ENSP00000344818 ENSP00000364133 |
| 15 | ENSP00000262741 ENSP00000263967 ENSP00000309845 ENSP00000251849 ENSP00000302486 ENSP00000366244 |
| 6 | ENSP00000262741 ENSP00000263967 ENSP00000270202 ENSP00000366563 |
| 6 | ENSP00000262741 ENSP00000263967 ENSP00000274335 ENSP00000264033 ENSP00000344818 ENSP00000350283 ENSP00000369497 |
| 10 | ENSP00000262741 ENSP00000263967 ENSP00000274335 ENSP00000303830 ENSP00000348986 ENSP00000375892 |
| 5 | ENSP00000262741 ENSP00000263967 ENSP00000274335 ENSP00000264033 ENSP00000344818 ENSP00000384273 |
| 5 | ENSP00000262904 ENSP00000267163 ENSP00000251849 ENSP00000302486 ENSP00000263025 |
| 39 | ENSP00000262904 ENSP00000267163 ENSP00000417281 ENSP00000270202 ENSP00000219476 ENSP00000263826 |
| 5 | ENSP00000262904 ENSP00000267163 ENSP00000417281 ENSP00000270202 ENSP00000263967 |
| 3 | ENSP00000262904 ENSP00000267163 ENSP00000227507 ENSP00000264657 |
| 5 | ENSP00000262904 ENSP00000267163 ENSP00000227507 ENSP00000264657 ENSP00000275493 ENSP00000265171 |
| 2 | ENSP00000262904 ENSP00000267163 ENSP00000265734 |
| 1 | ENSP00000262904 ENSP00000267163 |
| 4 | ENSP00000262904 ENSP00000267163 ENSP00000266970 ENSP00000269305 ENSP00000267868 |
| 16 | ENSP00000262904 ENSP00000267163 ENSP00000417281 ENSP00000270202 ENSP00000352121 ENSP00000269300 |
| 3 | ENSP00000262904 ENSP00000267163 ENSP00000266970 ENSP00000269305 |
| 4 | ENSP00000262904 ENSP00000267163 ENSP00000227507 ENSP00000344818 ENSP00000269571 |
| 3 | ENSP00000262904 ENSP00000267163 ENSP00000417281 ENSP00000270202 |
| 19 | ENSP00000262904 ENSP00000267163 ENSP00000227507 ENSP00000344818 ENSP00000003084 ENSP00000262613 ENSP00000338934 ENSP00000284384 ENSP00000342793 ENSP00000005257 ENSP00000019317 ENSP00000272519 |
| 5 | ENSP00000262904 ENSP00000267163 ENSP00000227507 ENSP00000344818 ENSP00000264033 ENSP00000274335 |
| 4 | ENSP00000262904 ENSP00000267163 ENSP00000227507 ENSP00000264657 ENSP00000275493 |
| 4 | ENSP00000262904 ENSP00000267163 ENSP00000251849 ENSP00000288602 |
| 5 | ENSP00000262904 ENSP00000267163 ENSP00000417281 ENSP00000270202 ENSP00000289153 |
| 5 | ENSP00000262904 ENSP00000267163 ENSP00000227507 ENSP00000264657 ENSP00000275493 ENSP00000295400 |
| 4 | ENSP00000262904 ENSP00000267163 ENSP00000251849 ENSP00000302486 |
| 6 | ENSP00000262904 ENSP00000267163 ENSP00000266970 ENSP00000269305 ENSP00000302564 |
| 10 | ENSP00000262904 ENSP00000267163 ENSP00000417281 ENSP00000270202 ENSP00000348461 ENSP00000269321 ENSP00000304283 |
| 5 | ENSP00000262904 ENSP00000267163 ENSP00000417281 ENSP00000270202 ENSP00000309103 |
| 6 | ENSP00000262904 ENSP00000267163 ENSP00000361423 ENSP00000339007 ENSP00000223023 ENSP00000314458 |
| 5 | ENSP00000262904 ENSP00000267163 ENSP00000266970 ENSP00000269305 ENSP00000321410 |
| 5 | ENSP00000262904 ENSP00000267163 ENSP00000227507 ENSP00000344818 ENSP00000347858 ENSP00000330237 |
| 4 | ENSP00000262904 ENSP00000267163 ENSP00000227507 ENSP00000344818 ENSP00000332973 |
| 4 | ENSP00000262904 ENSP00000267163 ENSP00000227507 ENSP00000344818 ENSP00000339151 |
| 4 | ENSP00000262904 ENSP00000267163 ENSP00000345571 ENSP00000329357 ENSP00000341551 |
| 15 | ENSP00000262904 ENSP00000267163 ENSP00000227507 ENSP00000344818 ENSP00000003084 ENSP00000262613 ENSP00000338934 ENSP00000284384 ENSP00000342793 |
| 4 | ENSP00000262904 ENSP00000267163 ENSP00000227507 ENSP00000264657 ENSP00000343204 |
| 2 | ENSP00000262904 ENSP00000267163 ENSP00000345571 |
| 5 | ENSP00000262904 ENSP00000267163 ENSP00000417281 ENSP00000270202 ENSP00000348461 |
| 5 | ENSP00000262904 ENSP00000267163 ENSP00000227507 ENSP00000344818 ENSP00000364133 ENSP00000351905 |
| 5 | ENSP00000262904 ENSP00000267163 ENSP00000417281 ENSP00000270202 ENSP00000352121 |
| 10 | ENSP00000262904 ENSP00000267163 ENSP00000266970 ENSP00000269305 ENSP00000353483 ENSP00000250894 ENSP00000352157 |
| 4 | ENSP00000262904 ENSP00000267163 ENSP00000266970 ENSP00000269305 ENSP00000353483 |
| 5 | ENSP00000262904 ENSP00000267163 ENSP00000227507 ENSP00000264657 ENSP00000354394 |
| 3 | ENSP00000262904 ENSP00000267163 ENSP00000257904 ENSP00000355153 |
| 2 | ENSP00000262904 ENSP00000267163 ENSP00000355249 |
| 8 | ENSP00000262904 ENSP00000267163 ENSP00000227507 ENSP00000344818 ENSP00000364133 ENSP00000351905 ENSP00000355896 |
| 4 | ENSP00000262904 ENSP00000267163 ENSP00000227507 ENSP00000344818 ENSP00000358622 |
| 4 | ENSP00000262904 ENSP00000267163 ENSP00000362649 ENSP00000384273 ENSP00000359424 |
| 5 | ENSP00000262904 ENSP00000267163 ENSP00000251849 ENSP00000309845 ENSP00000361120 |
| 5 | ENSP00000262904 ENSP00000267163 ENSP00000266970 ENSP00000269305 ENSP00000338018 ENSP00000361125 |
| 4 | ENSP00000262904 ENSP00000267163 ENSP00000227507 ENSP00000344818 ENSP00000364133 |
| 15 | ENSP00000262904 ENSP00000267163 ENSP00000251849 ENSP00000302486 ENSP00000366244 |
| 6 | ENSP00000262904 ENSP00000267163 ENSP00000417281 ENSP00000270202 ENSP00000366563 |
| 5 | ENSP00000262904 ENSP00000267163 ENSP00000266970 ENSP00000269305 ENSP00000267868 ENSP00000369497 |
| 13 | ENSP00000262904 ENSP00000267163 ENSP00000417281 ENSP00000270202 ENSP00000348986 ENSP00000375892 |
| 3 | ENSP00000262904 ENSP00000267163 ENSP00000362649 ENSP00000384273 |
| 39 | ENSP00000263025 ENSP00000302486 ENSP00000251849 ENSP00000300161 ENSP00000219476 ENSP00000263826 |
| 4 | ENSP00000263025 ENSP00000302486 ENSP00000251849 ENSP00000309845 ENSP00000263967 |
| 5 | ENSP00000263025 ENSP00000302486 ENSP00000215832 ENSP00000360266 ENSP00000263253 ENSP00000264657 |
| 6 | ENSP00000263025 ENSP00000302486 ENSP00000251849 ENSP00000335153 ENSP00000269571 ENSP00000265171 |
| 5 | ENSP00000263025 ENSP00000302486 ENSP00000251849 ENSP00000267163 ENSP00000265734 |
| 4 | ENSP00000263025 ENSP00000302486 ENSP00000251849 ENSP00000267163 |
| 5 | ENSP00000263025 ENSP00000302486 ENSP00000251849 ENSP00000335153 ENSP00000269305 ENSP00000267868 |
| 17 | ENSP00000263025 ENSP00000302486 ENSP00000251849 ENSP00000335153 ENSP00000270202 ENSP00000352121 ENSP00000269300 |
| 4 | ENSP00000263025 ENSP00000302486 ENSP00000251849 ENSP00000335153 ENSP00000269305 |
| 4 | ENSP00000263025 ENSP00000302486 ENSP00000251849 ENSP00000335153 ENSP00000269571 |
| 4 | ENSP00000263025 ENSP00000302486 ENSP00000251849 ENSP00000335153 ENSP00000270202 |
| 20 | ENSP00000263025 ENSP00000302486 ENSP00000251849 ENSP00000335153 ENSP00000297494 ENSP00000349467 ENSP00000005257 ENSP00000019317 ENSP00000272519 |
| 5 | ENSP00000263025 ENSP00000302486 ENSP00000251849 ENSP00000309845 ENSP00000263967 ENSP00000274335 |
| 5 | ENSP00000263025 ENSP00000302486 ENSP00000251849 ENSP00000335153 ENSP00000269571 ENSP00000275493 |
| 3 | ENSP00000263025 ENSP00000302486 ENSP00000251849 ENSP00000288602 |
| 6 | ENSP00000263025 ENSP00000302486 ENSP00000251849 ENSP00000335153 ENSP00000270202 ENSP00000289153 |
| 6 | ENSP00000263025 ENSP00000302486 ENSP00000251849 ENSP00000335153 ENSP00000269571 ENSP00000275493 ENSP00000295400 |
| 1 | ENSP00000263025 ENSP00000302486 |
| 5 | ENSP00000263025 ENSP00000302486 ENSP00000251849 ENSP00000309503 ENSP00000309103 ENSP00000302564 |
| 11 | ENSP00000263025 ENSP00000302486 ENSP00000251849 ENSP00000335153 ENSP00000270202 ENSP00000348461 ENSP00000269321 ENSP00000304283 |
| 4 | ENSP00000263025 ENSP00000302486 ENSP00000251849 ENSP00000309503 ENSP00000309103 |
| 7 | ENSP00000263025 ENSP00000302486 ENSP00000251849 ENSP00000335153 ENSP00000269571 ENSP00000339007 ENSP00000223023 ENSP00000314458 |
| 4 | ENSP00000263025 ENSP00000302486 ENSP00000215832 ENSP00000360266 ENSP00000321410 |
| 6 | ENSP00000263025 ENSP00000302486 ENSP00000215832 ENSP00000360266 ENSP00000344818 ENSP00000347858 ENSP00000330237 |
| 5 | ENSP00000263025 ENSP00000302486 ENSP00000215832 ENSP00000360266 ENSP00000332973 |
| 5 | ENSP00000263025 ENSP00000302486 ENSP00000215832 ENSP00000360266 ENSP00000344818 ENSP00000339151 |
| 5 | ENSP00000263025 ENSP00000302486 ENSP00000215832 ENSP00000360266 ENSP00000263253 ENSP00000341551 |
| 16 | ENSP00000263025 ENSP00000302486 ENSP00000215832 ENSP00000360266 ENSP00000344818 ENSP00000003084 ENSP00000262613 ENSP00000338934 ENSP00000284384 ENSP00000342793 |
| 6 | ENSP00000263025 ENSP00000302486 ENSP00000215832 ENSP00000360266 ENSP00000263253 ENSP00000264657 ENSP00000343204 |
| 5 | ENSP00000263025 ENSP00000302486 ENSP00000251849 ENSP00000267163 ENSP00000345571 |
| 6 | ENSP00000263025 ENSP00000302486 ENSP00000251849 ENSP00000335153 ENSP00000270202 ENSP00000348461 |
| 6 | ENSP00000263025 ENSP00000302486 ENSP00000215832 ENSP00000360266 ENSP00000344818 ENSP00000364133 ENSP00000351905 |
| 6 | ENSP00000263025 ENSP00000302486 ENSP00000251849 ENSP00000335153 ENSP00000270202 ENSP00000352121 |
| 10 | ENSP00000263025 ENSP00000302486 ENSP00000215832 ENSP00000360266 ENSP00000353483 ENSP00000250894 ENSP00000352157 |
| 4 | ENSP00000263025 ENSP00000302486 ENSP00000215832 ENSP00000360266 ENSP00000353483 |
| 5 | ENSP00000263025 ENSP00000302486 ENSP00000215832 ENSP00000360266 ENSP00000263253 ENSP00000354394 |
| 5 | ENSP00000263025 ENSP00000302486 ENSP00000251849 ENSP00000335153 ENSP00000269305 ENSP00000355153 |
| 5 | ENSP00000263025 ENSP00000302486 ENSP00000251849 ENSP00000267163 ENSP00000355249 |
| 9 | ENSP00000263025 ENSP00000302486 ENSP00000215832 ENSP00000360266 ENSP00000344818 ENSP00000364133 ENSP00000351905 ENSP00000355896 |
| 5 | ENSP00000263025 ENSP00000302486 ENSP00000215832 ENSP00000360266 ENSP00000344818 ENSP00000358622 |
| 6 | ENSP00000263025 ENSP00000302486 ENSP00000215832 ENSP00000360266 ENSP00000344818 ENSP00000216797 ENSP00000359424 |
| 4 | ENSP00000263025 ENSP00000302486 ENSP00000251849 ENSP00000309845 ENSP00000361120 |
| 5 | ENSP00000263025 ENSP00000302486 ENSP00000251849 ENSP00000335153 ENSP00000338018 ENSP00000361125 |
| 5 | ENSP00000263025 ENSP00000302486 ENSP00000215832 ENSP00000360266 ENSP00000344818 ENSP00000364133 |
| 12 | ENSP00000263025 ENSP00000302486 ENSP00000366244 |
| 7 | ENSP00000263025 ENSP00000302486 ENSP00000251849 ENSP00000335153 ENSP00000270202 ENSP00000366563 |
| 6 | ENSP00000263025 ENSP00000302486 ENSP00000251849 ENSP00000335153 ENSP00000269305 ENSP00000267868 ENSP00000369497 |
| 13 | ENSP00000263025 ENSP00000302486 ENSP00000251849 ENSP00000309845 ENSP00000263967 ENSP00000274335 ENSP00000303830 ENSP00000348986 ENSP00000375892 |
| 5 | ENSP00000263025 ENSP00000302486 ENSP00000215832 ENSP00000360266 ENSP00000263253 ENSP00000384273 |
| 38 | ENSP00000263826 ENSP00000219476 ENSP00000270202 ENSP00000263967 |
| 39 | ENSP00000263826 ENSP00000219476 ENSP00000354558 ENSP00000264657 |
| 39 | ENSP00000263826 ENSP00000219476 ENSP00000270202 ENSP00000344818 ENSP00000275493 ENSP00000265171 |
| 39 | ENSP00000263826 ENSP00000219476 ENSP00000270202 ENSP00000344818 ENSP00000227507 ENSP00000265734 |
| 38 | ENSP00000263826 ENSP00000219476 ENSP00000270202 ENSP00000417281 ENSP00000267163 |
| 39 | ENSP00000263826 ENSP00000219476 ENSP00000270202 ENSP00000335153 ENSP00000269305 ENSP00000267868 |
| 49 | ENSP00000263826 ENSP00000219476 ENSP00000270202 ENSP00000352121 ENSP00000269300 |
| 38 | ENSP00000263826 ENSP00000219476 ENSP00000270202 ENSP00000335153 ENSP00000269305 |
| 38 | ENSP00000263826 ENSP00000219476 ENSP00000270202 ENSP00000335153 ENSP00000269571 |
| 36 | ENSP00000263826 ENSP00000219476 ENSP00000270202 |
| 53 | ENSP00000263826 ENSP00000219476 ENSP00000270202 ENSP00000297494 ENSP00000349467 ENSP00000005257 ENSP00000019317 ENSP00000272519 |
| 39 | ENSP00000263826 ENSP00000219476 ENSP00000270202 ENSP00000263967 ENSP00000274335 |
| 38 | ENSP00000263826 ENSP00000219476 ENSP00000270202 ENSP00000344818 ENSP00000275493 |
| 38 | ENSP00000263826 ENSP00000219476 ENSP00000300161 ENSP00000251849 ENSP00000288602 |
| 38 | ENSP00000263826 ENSP00000219476 ENSP00000270202 ENSP00000289153 |
| 39 | ENSP00000263826 ENSP00000219476 ENSP00000270202 ENSP00000344818 ENSP00000275493 ENSP00000295400 |
| 38 | ENSP00000263826 ENSP00000219476 ENSP00000300161 ENSP00000251849 ENSP00000302486 |
| 38 | ENSP00000263826 ENSP00000219476 ENSP00000309503 ENSP00000309103 ENSP00000302564 |
| 43 | ENSP00000263826 ENSP00000219476 ENSP00000270202 ENSP00000348461 ENSP00000269321 ENSP00000304283 |
| 37 | ENSP00000263826 ENSP00000219476 ENSP00000309503 ENSP00000309103 |
| 40 | ENSP00000263826 ENSP00000219476 ENSP00000270202 ENSP00000348461 ENSP00000268182 ENSP00000314458 |
| 39 | ENSP00000263826 ENSP00000219476 ENSP00000270202 ENSP00000344818 ENSP00000360266 ENSP00000321410 |
| 39 | ENSP00000263826 ENSP00000219476 ENSP00000270202 ENSP00000344818 ENSP00000347858 ENSP00000330237 |
| 38 | ENSP00000263826 ENSP00000219476 ENSP00000270202 ENSP00000344818 ENSP00000332973 |
| 38 | ENSP00000263826 ENSP00000219476 ENSP00000270202 ENSP00000344818 ENSP00000339151 |
| 38 | ENSP00000263826 ENSP00000219476 ENSP00000270202 ENSP00000344818 ENSP00000341551 |
| 49 | ENSP00000263826 ENSP00000219476 ENSP00000270202 ENSP00000348461 ENSP00000298316 ENSP00000342793 |
| 40 | ENSP00000263826 ENSP00000219476 ENSP00000354558 ENSP00000264657 ENSP00000343204 |
| 38 | ENSP00000263826 ENSP00000219476 ENSP00000270202 ENSP00000344818 ENSP00000345571 |
| 38 | ENSP00000263826 ENSP00000219476 ENSP00000270202 ENSP00000348461 |
| 39 | ENSP00000263826 ENSP00000219476 ENSP00000270202 ENSP00000344818 ENSP00000364133 ENSP00000351905 |
| 38 | ENSP00000263826 ENSP00000219476 ENSP00000270202 ENSP00000352121 |
| 45 | ENSP00000263826 ENSP00000219476 ENSP00000270202 ENSP00000335153 ENSP00000269305 ENSP00000353483 ENSP00000250894 ENSP00000352157 |
| 39 | ENSP00000263826 ENSP00000219476 ENSP00000270202 ENSP00000335153 ENSP00000269305 ENSP00000353483 |
| 39 | ENSP00000263826 ENSP00000219476 ENSP00000270202 ENSP00000344818 ENSP00000275493 ENSP00000354394 |
| 38 | ENSP00000263826 ENSP00000219476 ENSP00000270202 ENSP00000417281 ENSP00000355153 |
| 39 | ENSP00000263826 ENSP00000219476 ENSP00000270202 ENSP00000417281 ENSP00000267163 ENSP00000355249 |
| 42 | ENSP00000263826 ENSP00000219476 ENSP00000270202 ENSP00000344818 ENSP00000364133 ENSP00000351905 ENSP00000355896 |
| 38 | ENSP00000263826 ENSP00000219476 ENSP00000270202 ENSP00000344818 ENSP00000358622 |
| 39 | ENSP00000263826 ENSP00000219476 ENSP00000270202 ENSP00000359424 |
| 39 | ENSP00000263826 ENSP00000219476 ENSP00000300161 ENSP00000251849 ENSP00000309845 ENSP00000361120 |
| 39 | ENSP00000263826 ENSP00000219476 ENSP00000270202 ENSP00000335153 ENSP00000338018 ENSP00000361125 |
| 38 | ENSP00000263826 ENSP00000219476 ENSP00000270202 ENSP00000344818 ENSP00000364133 |
| 49 | ENSP00000263826 ENSP00000219476 ENSP00000300161 ENSP00000251849 ENSP00000302486 ENSP00000366244 |
| 39 | ENSP00000263826 ENSP00000219476 ENSP00000270202 ENSP00000366563 |
| 39 | ENSP00000263826 ENSP00000219476 ENSP00000270202 ENSP00000344818 ENSP00000350283 ENSP00000369497 |
| 46 | ENSP00000263826 ENSP00000219476 ENSP00000270202 ENSP00000348986 ENSP00000375892 |
| 38 | ENSP00000263826 ENSP00000219476 ENSP00000270202 ENSP00000344818 ENSP00000384273 |
| 3 | ENSP00000263967 ENSP00000304895 ENSP00000343204 ENSP00000264657 |
| 4 | ENSP00000263967 ENSP00000274335 ENSP00000267101 ENSP00000265171 |
| 5 | ENSP00000263967 ENSP00000304895 ENSP00000343204 ENSP00000264657 ENSP00000227507 ENSP00000265734 |
| 4 | ENSP00000263967 ENSP00000309845 ENSP00000251849 ENSP00000267163 |
| 5 | ENSP00000263967 ENSP00000309845 ENSP00000251849 ENSP00000335153 ENSP00000269305 ENSP00000267868 |
| 15 | ENSP00000263967 ENSP00000270202 ENSP00000352121 ENSP00000269300 |
| 4 | ENSP00000263967 ENSP00000309845 ENSP00000251849 ENSP00000335153 ENSP00000269305 |
| 3 | ENSP00000263967 ENSP00000274335 ENSP00000267101 ENSP00000269571 |
| 2 | ENSP00000263967 ENSP00000270202 |
| 19 | ENSP00000263967 ENSP00000270202 ENSP00000297494 ENSP00000349467 ENSP00000005257 ENSP00000019317 ENSP00000272519 |
| 1 | ENSP00000263967 ENSP00000274335 |
| 3 | ENSP00000263967 ENSP00000274335 ENSP00000264033 ENSP00000275493 |
| 3 | ENSP00000263967 ENSP00000309845 ENSP00000288602 |
| 4 | ENSP00000263967 ENSP00000270202 ENSP00000289153 |
| 4 | ENSP00000263967 ENSP00000274335 ENSP00000264033 ENSP00000275493 ENSP00000295400 |
| 3 | ENSP00000263967 ENSP00000309845 ENSP00000251849 ENSP00000302486 |
| 5 | ENSP00000263967 ENSP00000270202 ENSP00000309103 ENSP00000302564 |
| 9 | ENSP00000263967 ENSP00000274335 ENSP00000264033 ENSP00000302269 ENSP00000304283 |
| 4 | ENSP00000263967 ENSP00000270202 ENSP00000309103 |
| 4 | ENSP00000263967 ENSP00000274335 ENSP00000339007 ENSP00000223023 ENSP00000314458 |
| 5 | ENSP00000263967 ENSP00000274335 ENSP00000264033 ENSP00000344818 ENSP00000360266 ENSP00000321410 |
| 5 | ENSP00000263967 ENSP00000274335 ENSP00000264033 ENSP00000344818 ENSP00000347858 ENSP00000330237 |
| 4 | ENSP00000263967 ENSP00000274335 ENSP00000264033 ENSP00000344818 ENSP00000332973 |
| 4 | ENSP00000263967 ENSP00000274335 ENSP00000264033 ENSP00000344818 ENSP00000339151 |
| 4 | ENSP00000263967 ENSP00000274335 ENSP00000264033 ENSP00000344818 ENSP00000341551 |
| 15 | ENSP00000263967 ENSP00000270202 ENSP00000348461 ENSP00000298316 ENSP00000342793 |
| 2 | ENSP00000263967 ENSP00000304895 ENSP00000343204 |
| 4 | ENSP00000263967 ENSP00000274335 ENSP00000264033 ENSP00000344818 ENSP00000345571 |
| 4 | ENSP00000263967 ENSP00000270202 ENSP00000348461 |
| 5 | ENSP00000263967 ENSP00000274335 ENSP00000264033 ENSP00000344818 ENSP00000364133 ENSP00000351905 |
| 4 | ENSP00000263967 ENSP00000270202 ENSP00000352121 |
| 9 | ENSP00000263967 ENSP00000304895 ENSP00000353483 ENSP00000250894 ENSP00000352157 |
| 3 | ENSP00000263967 ENSP00000304895 ENSP00000353483 |
| 3 | ENSP00000263967 ENSP00000304895 ENSP00000371067 ENSP00000354394 |
| 4 | ENSP00000263967 ENSP00000270202 ENSP00000417281 ENSP00000355153 |
| 5 | ENSP00000263967 ENSP00000309845 ENSP00000251849 ENSP00000267163 ENSP00000355249 |
| 8 | ENSP00000263967 ENSP00000274335 ENSP00000264033 ENSP00000344818 ENSP00000364133 ENSP00000351905 ENSP00000355896 |
| 4 | ENSP00000263967 ENSP00000274335 ENSP00000264033 ENSP00000344818 ENSP00000358622 |
| 5 | ENSP00000263967 ENSP00000270202 ENSP00000359424 |
| 2 | ENSP00000263967 ENSP00000309845 ENSP00000361120 |
| 5 | ENSP00000263967 ENSP00000309845 ENSP00000251849 ENSP00000335153 ENSP00000338018 ENSP00000361125 |
| 4 | ENSP00000263967 ENSP00000274335 ENSP00000264033 ENSP00000344818 ENSP00000364133 |
| 14 | ENSP00000263967 ENSP00000309845 ENSP00000251849 ENSP00000302486 ENSP00000366244 |
| 5 | ENSP00000263967 ENSP00000270202 ENSP00000366563 |
| 5 | ENSP00000263967 ENSP00000274335 ENSP00000264033 ENSP00000344818 ENSP00000350283 ENSP00000369497 |
| 9 | ENSP00000263967 ENSP00000274335 ENSP00000303830 ENSP00000348986 ENSP00000375892 |
| 4 | ENSP00000263967 ENSP00000274335 ENSP00000264033 ENSP00000344818 ENSP00000384273 |
| 2 | ENSP00000264657 ENSP00000275493 ENSP00000265171 |
| 2 | ENSP00000264657 ENSP00000227507 ENSP00000265734 |
| 2 | ENSP00000264657 ENSP00000227507 ENSP00000267163 |
| 3 | ENSP00000264657 ENSP00000263253 ENSP00000269305 ENSP00000267868 |
| 16 | ENSP00000264657 ENSP00000227507 ENSP00000344818 ENSP00000270202 ENSP00000352121 ENSP00000269300 |
| 2 | ENSP00000264657 ENSP00000263253 ENSP00000269305 |
| 2 | ENSP00000264657 ENSP00000275493 ENSP00000269571 |
| 3 | ENSP00000264657 ENSP00000227507 ENSP00000344818 ENSP00000270202 |
| 17 | ENSP00000264657 ENSP00000350941 ENSP00000282561 ENSP00000284384 ENSP00000342793 ENSP00000005257 ENSP00000019317 ENSP00000272519 |
| 3 | ENSP00000264657 ENSP00000275493 ENSP00000264033 ENSP00000274335 |
| 1 | ENSP00000264657 ENSP00000275493 |
| 5 | ENSP00000264657 ENSP00000227507 ENSP00000267163 ENSP00000251849 ENSP00000288602 |
| 5 | ENSP00000264657 ENSP00000227507 ENSP00000344818 ENSP00000270202 ENSP00000289153 |
| 2 | ENSP00000264657 ENSP00000275493 ENSP00000295400 |
| 4 | ENSP00000264657 ENSP00000263253 ENSP00000360266 ENSP00000215832 ENSP00000302486 |
| 5 | ENSP00000264657 ENSP00000263253 ENSP00000269305 ENSP00000302564 |
| 9 | ENSP00000264657 ENSP00000350941 ENSP00000304283 |
| 4 | ENSP00000264657 ENSP00000263253 ENSP00000269305 ENSP00000329623 ENSP00000309103 |
| 4 | ENSP00000264657 ENSP00000275493 ENSP00000339007 ENSP00000223023 ENSP00000314458 |
| 3 | ENSP00000264657 ENSP00000263253 ENSP00000360266 ENSP00000321410 |
| 4 | ENSP00000264657 ENSP00000227507 ENSP00000344818 ENSP00000347858 ENSP00000330237 |
| 2 | ENSP00000264657 ENSP00000263253 ENSP00000332973 |
| 3 | ENSP00000264657 ENSP00000227507 ENSP00000344818 ENSP00000339151 |
| 2 | ENSP00000264657 ENSP00000263253 ENSP00000341551 |
| 13 | ENSP00000264657 ENSP00000350941 ENSP00000282561 ENSP00000284384 ENSP00000342793 |
| 1 | ENSP00000264657 ENSP00000343204 |
| 3 | ENSP00000264657 ENSP00000227507 ENSP00000267163 ENSP00000345571 |
| 4 | ENSP00000264657 ENSP00000348461 |
| 4 | ENSP00000264657 ENSP00000227507 ENSP00000344818 ENSP00000364133 ENSP00000351905 |
| 5 | ENSP00000264657 ENSP00000227507 ENSP00000344818 ENSP00000270202 ENSP00000352121 |
| 9 | ENSP00000264657 ENSP00000263253 ENSP00000269305 ENSP00000353483 ENSP00000250894 ENSP00000352157 |
| 3 | ENSP00000264657 ENSP00000263253 ENSP00000269305 ENSP00000353483 |
| 2 | ENSP00000264657 ENSP00000354394 |
| 3 | ENSP00000264657 ENSP00000227507 ENSP00000257904 ENSP00000355153 |
| 3 | ENSP00000264657 ENSP00000227507 ENSP00000267163 ENSP00000355249 |
| 7 | ENSP00000264657 ENSP00000227507 ENSP00000344818 ENSP00000364133 ENSP00000351905 ENSP00000355896 |
| 3 | ENSP00000264657 ENSP00000227507 ENSP00000344818 ENSP00000358622 |
| 3 | ENSP00000264657 ENSP00000263253 ENSP00000384273 ENSP00000359424 |
| 5 | ENSP00000264657 ENSP00000350941 ENSP00000309845 ENSP00000361120 |
| 3 | ENSP00000264657 ENSP00000263253 ENSP00000338018 ENSP00000361125 |
| 3 | ENSP00000264657 ENSP00000227507 ENSP00000344818 ENSP00000364133 |
| 15 | ENSP00000264657 ENSP00000263253 ENSP00000360266 ENSP00000215832 ENSP00000302486 ENSP00000366244 |
| 6 | ENSP00000264657 ENSP00000227507 ENSP00000344818 ENSP00000270202 ENSP00000366563 |
| 4 | ENSP00000264657 ENSP00000263253 ENSP00000269305 ENSP00000267868 ENSP00000369497 |
| 10 | ENSP00000264657 ENSP00000343204 ENSP00000304895 ENSP00000303830 ENSP00000348986 ENSP00000375892 |
| 2 | ENSP00000264657 ENSP00000263253 ENSP00000384273 |
| 4 | ENSP00000265171 ENSP00000275493 ENSP00000264657 ENSP00000227507 ENSP00000265734 |
| 4 | ENSP00000265171 ENSP00000275493 ENSP00000264657 ENSP00000227507 ENSP00000267163 |
| 4 | ENSP00000265171 ENSP00000275493 ENSP00000344818 ENSP00000269305 ENSP00000267868 |
| 16 | ENSP00000265171 ENSP00000275493 ENSP00000344818 ENSP00000270202 ENSP00000352121 ENSP00000269300 |
| 3 | ENSP00000265171 ENSP00000275493 ENSP00000344818 ENSP00000269305 |
| 2 | ENSP00000265171 ENSP00000269571 |
| 3 | ENSP00000265171 ENSP00000275493 ENSP00000344818 ENSP00000270202 |
| 18 | ENSP00000265171 ENSP00000275493 ENSP00000350941 ENSP00000282561 ENSP00000284384 ENSP00000342793 ENSP00000005257 ENSP00000019317 ENSP00000272519 |
| 3 | ENSP00000265171 ENSP00000275493 ENSP00000264033 ENSP00000274335 |
| 1 | ENSP00000265171 ENSP00000275493 |
| 5 | ENSP00000265171 ENSP00000269571 ENSP00000335153 ENSP00000251849 ENSP00000288602 |
| 5 | ENSP00000265171 ENSP00000275493 ENSP00000344818 ENSP00000270202 ENSP00000289153 |
| 2 | ENSP00000265171 ENSP00000275493 ENSP00000295400 |
| 5 | ENSP00000265171 ENSP00000275493 ENSP00000344818 ENSP00000360266 ENSP00000215832 ENSP00000302486 |
| 5 | ENSP00000265171 ENSP00000275493 ENSP00000344818 ENSP00000358022 ENSP00000293288 ENSP00000302564 |
| 9 | ENSP00000265171 ENSP00000275493 ENSP00000264033 ENSP00000302269 ENSP00000304283 |
| 5 | ENSP00000265171 ENSP00000275493 ENSP00000344818 ENSP00000270202 ENSP00000309103 |
| 4 | ENSP00000265171 ENSP00000339007 ENSP00000223023 ENSP00000314458 |
| 4 | ENSP00000265171 ENSP00000275493 ENSP00000344818 ENSP00000360266 ENSP00000321410 |
| 4 | ENSP00000265171 ENSP00000275493 ENSP00000344818 ENSP00000347858 ENSP00000330237 |
| 3 | ENSP00000265171 ENSP00000275493 ENSP00000344818 ENSP00000332973 |
| 3 | ENSP00000265171 ENSP00000275493 ENSP00000344818 ENSP00000339151 |
| 3 | ENSP00000265171 ENSP00000275493 ENSP00000344818 ENSP00000341551 |
| 14 | ENSP00000265171 ENSP00000275493 ENSP00000350941 ENSP00000282561 ENSP00000284384 ENSP00000342793 |
| 3 | ENSP00000265171 ENSP00000275493 ENSP00000264657 ENSP00000343204 |
| 3 | ENSP00000265171 ENSP00000275493 ENSP00000344818 ENSP00000345571 |
| 5 | ENSP00000265171 ENSP00000275493 ENSP00000344818 ENSP00000270202 ENSP00000348461 |
| 4 | ENSP00000265171 ENSP00000275493 ENSP00000344818 ENSP00000364133 ENSP00000351905 |
| 5 | ENSP00000265171 ENSP00000275493 ENSP00000344818 ENSP00000270202 ENSP00000352121 |
| 10 | ENSP00000265171 ENSP00000275493 ENSP00000344818 ENSP00000269305 ENSP00000353483 ENSP00000250894 ENSP00000352157 |
| 4 | ENSP00000265171 ENSP00000275493 ENSP00000344818 ENSP00000269305 ENSP00000353483 |
| 2 | ENSP00000265171 ENSP00000275493 ENSP00000354394 |
| 4 | ENSP00000265171 ENSP00000275493 ENSP00000344818 ENSP00000269305 ENSP00000355153 |
| 5 | ENSP00000265171 ENSP00000275493 ENSP00000264657 ENSP00000227507 ENSP00000267163 ENSP00000355249 |
| 7 | ENSP00000265171 ENSP00000275493 ENSP00000344818 ENSP00000364133 ENSP00000351905 ENSP00000355896 |
| 3 | ENSP00000265171 ENSP00000275493 ENSP00000344818 ENSP00000358622 |
| 4 | ENSP00000265171 ENSP00000275493 ENSP00000344818 ENSP00000216797 ENSP00000359424 |
| 5 | ENSP00000265171 ENSP00000275493 ENSP00000384675 ENSP00000309845 ENSP00000361120 |
| 4 | ENSP00000265171 ENSP00000275493 ENSP00000344818 ENSP00000338018 ENSP00000361125 |
| 3 | ENSP00000265171 ENSP00000275493 ENSP00000344818 ENSP00000364133 |
| 16 | ENSP00000265171 ENSP00000275493 ENSP00000344818 ENSP00000360266 ENSP00000215832 ENSP00000302486 ENSP00000366244 |
| 6 | ENSP00000265171 ENSP00000275493 ENSP00000344818 ENSP00000270202 ENSP00000366563 |
| 4 | ENSP00000265171 ENSP00000275493 ENSP00000344818 ENSP00000350283 ENSP00000369497 |
| 10 | ENSP00000265171 ENSP00000275493 ENSP00000340944 ENSP00000303830 ENSP00000348986 ENSP00000375892 |
| 3 | ENSP00000265171 ENSP00000275493 ENSP00000344818 ENSP00000384273 |
| 1 | ENSP00000265734 ENSP00000267163 |
| 3 | ENSP00000265734 ENSP00000244741 ENSP00000269305 ENSP00000267868 |
| 16 | ENSP00000265734 ENSP00000228872 ENSP00000270202 ENSP00000352121 ENSP00000269300 |
| 2 | ENSP00000265734 ENSP00000244741 ENSP00000269305 |
| 3 | ENSP00000265734 ENSP00000227507 ENSP00000344818 ENSP00000269571 |
| 3 | ENSP00000265734 ENSP00000228872 ENSP00000270202 |
| 18 | ENSP00000265734 ENSP00000227507 ENSP00000344818 ENSP00000003084 ENSP00000262613 ENSP00000338934 ENSP00000284384 ENSP00000342793 ENSP00000005257 ENSP00000019317 ENSP00000272519 |
| 4 | ENSP00000265734 ENSP00000227507 ENSP00000344818 ENSP00000264033 ENSP00000274335 |
| 3 | ENSP00000265734 ENSP00000227507 ENSP00000264657 ENSP00000275493 |
| 4 | ENSP00000265734 ENSP00000267163 ENSP00000251849 ENSP00000288602 |
| 5 | ENSP00000265734 ENSP00000228872 ENSP00000270202 ENSP00000289153 |
| 4 | ENSP00000265734 ENSP00000227507 ENSP00000264657 ENSP00000275493 ENSP00000295400 |
| 4 | ENSP00000265734 ENSP00000267163 ENSP00000251849 ENSP00000302486 |
| 5 | ENSP00000265734 ENSP00000244741 ENSP00000269305 ENSP00000302564 |
| 10 | ENSP00000265734 ENSP00000227507 ENSP00000344818 ENSP00000264033 ENSP00000302269 ENSP00000304283 |
| 4 | ENSP00000265734 ENSP00000244741 ENSP00000269305 ENSP00000329623 ENSP00000309103 |
| 6 | ENSP00000265734 ENSP00000227507 ENSP00000344818 ENSP00000264033 ENSP00000339007 ENSP00000223023 ENSP00000314458 |
| 4 | ENSP00000265734 ENSP00000244741 ENSP00000269305 ENSP00000321410 |
| 4 | ENSP00000265734 ENSP00000227507 ENSP00000344818 ENSP00000347858 ENSP00000330237 |
| 3 | ENSP00000265734 ENSP00000227507 ENSP00000344818 ENSP00000332973 |
| 3 | ENSP00000265734 ENSP00000227507 ENSP00000344818 ENSP00000339151 |
| 3 | ENSP00000265734 ENSP00000227507 ENSP00000344818 ENSP00000341551 |
| 14 | ENSP00000265734 ENSP00000227507 ENSP00000344818 ENSP00000003084 ENSP00000262613 ENSP00000338934 ENSP00000284384 ENSP00000342793 |
| 3 | ENSP00000265734 ENSP00000227507 ENSP00000264657 ENSP00000343204 |
| 2 | ENSP00000265734 ENSP00000267163 ENSP00000345571 |
| 5 | ENSP00000265734 ENSP00000228872 ENSP00000270202 ENSP00000348461 |
| 4 | ENSP00000265734 ENSP00000227507 ENSP00000344818 ENSP00000364133 ENSP00000351905 |
| 5 | ENSP00000265734 ENSP00000228872 ENSP00000270202 ENSP00000352121 |
| 9 | ENSP00000265734 ENSP00000244741 ENSP00000269305 ENSP00000353483 ENSP00000250894 ENSP00000352157 |
| 3 | ENSP00000265734 ENSP00000244741 ENSP00000269305 ENSP00000353483 |
| 4 | ENSP00000265734 ENSP00000227507 ENSP00000264657 ENSP00000354394 |
| 1 | ENSP00000265734 ENSP00000355153 |
| 2 | ENSP00000265734 ENSP00000267163 ENSP00000355249 |
| 7 | ENSP00000265734 ENSP00000227507 ENSP00000344818 ENSP00000364133 ENSP00000351905 ENSP00000355896 |
| 3 | ENSP00000265734 ENSP00000227507 ENSP00000344818 ENSP00000358622 |
| 4 | ENSP00000265734 ENSP00000227507 ENSP00000344818 ENSP00000216797 ENSP00000359424 |
| 5 | ENSP00000265734 ENSP00000267163 ENSP00000251849 ENSP00000309845 ENSP00000361120 |
| 4 | ENSP00000265734 ENSP00000244741 ENSP00000269305 ENSP00000338018 ENSP00000361125 |
| 3 | ENSP00000265734 ENSP00000227507 ENSP00000344818 ENSP00000364133 |
| 15 | ENSP00000265734 ENSP00000267163 ENSP00000251849 ENSP00000302486 ENSP00000366244 |
| 6 | ENSP00000265734 ENSP00000228872 ENSP00000270202 ENSP00000366563 |
| 4 | ENSP00000265734 ENSP00000244741 ENSP00000269305 ENSP00000267868 ENSP00000369497 |
| 12 | ENSP00000265734 ENSP00000227507 ENSP00000344818 ENSP00000264033 ENSP00000274335 ENSP00000303830 ENSP00000348986 ENSP00000375892 |
| 3 | ENSP00000265734 ENSP00000227507 ENSP00000344818 ENSP00000384273 |
| 3 | ENSP00000267163 ENSP00000266970 ENSP00000269305 ENSP00000267868 |
| 15 | ENSP00000267163 ENSP00000417281 ENSP00000270202 ENSP00000352121 ENSP00000269300 |
| 2 | ENSP00000267163 ENSP00000266970 ENSP00000269305 |
| 3 | ENSP00000267163 ENSP00000227507 ENSP00000344818 ENSP00000269571 |
| 2 | ENSP00000267163 ENSP00000417281 ENSP00000270202 |
| 18 | ENSP00000267163 ENSP00000227507 ENSP00000344818 ENSP00000003084 ENSP00000262613 ENSP00000338934 ENSP00000284384 ENSP00000342793 ENSP00000005257 ENSP00000019317 ENSP00000272519 |
| 4 | ENSP00000267163 ENSP00000227507 ENSP00000344818 ENSP00000264033 ENSP00000274335 |
| 3 | ENSP00000267163 ENSP00000227507 ENSP00000264657 ENSP00000275493 |
| 3 | ENSP00000267163 ENSP00000251849 ENSP00000288602 |
| 4 | ENSP00000267163 ENSP00000417281 ENSP00000270202 ENSP00000289153 |
| 4 | ENSP00000267163 ENSP00000227507 ENSP00000264657 ENSP00000275493 ENSP00000295400 |
| 3 | ENSP00000267163 ENSP00000251849 ENSP00000302486 |
| 5 | ENSP00000267163 ENSP00000266970 ENSP00000269305 ENSP00000302564 |
| 9 | ENSP00000267163 ENSP00000417281 ENSP00000270202 ENSP00000348461 ENSP00000269321 ENSP00000304283 |
| 4 | ENSP00000267163 ENSP00000417281 ENSP00000270202 ENSP00000309103 |
| 5 | ENSP00000267163 ENSP00000361423 ENSP00000339007 ENSP00000223023 ENSP00000314458 |
| 4 | ENSP00000267163 ENSP00000266970 ENSP00000269305 ENSP00000321410 |
| 4 | ENSP00000267163 ENSP00000227507 ENSP00000344818 ENSP00000347858 ENSP00000330237 |
| 3 | ENSP00000267163 ENSP00000227507 ENSP00000344818 ENSP00000332973 |
| 3 | ENSP00000267163 ENSP00000227507 ENSP00000344818 ENSP00000339151 |
| 3 | ENSP00000267163 ENSP00000345571 ENSP00000329357 ENSP00000341551 |
| 14 | ENSP00000267163 ENSP00000227507 ENSP00000344818 ENSP00000003084 ENSP00000262613 ENSP00000338934 ENSP00000284384 ENSP00000342793 |
| 3 | ENSP00000267163 ENSP00000227507 ENSP00000264657 ENSP00000343204 |
| 1 | ENSP00000267163 ENSP00000345571 |
| 4 | ENSP00000267163 ENSP00000417281 ENSP00000270202 ENSP00000348461 |
| 4 | ENSP00000267163 ENSP00000227507 ENSP00000344818 ENSP00000364133 ENSP00000351905 |
| 4 | ENSP00000267163 ENSP00000417281 ENSP00000270202 ENSP00000352121 |
| 9 | ENSP00000267163 ENSP00000266970 ENSP00000269305 ENSP00000353483 ENSP00000250894 ENSP00000352157 |
| 3 | ENSP00000267163 ENSP00000266970 ENSP00000269305 ENSP00000353483 |
| 4 | ENSP00000267163 ENSP00000227507 ENSP00000264657 ENSP00000354394 |
| 2 | ENSP00000267163 ENSP00000257904 ENSP00000355153 |
| 1 | ENSP00000267163 ENSP00000355249 |
| 7 | ENSP00000267163 ENSP00000227507 ENSP00000344818 ENSP00000364133 ENSP00000351905 ENSP00000355896 |
| 3 | ENSP00000267163 ENSP00000227507 ENSP00000344818 ENSP00000358622 |
| 3 | ENSP00000267163 ENSP00000362649 ENSP00000384273 ENSP00000359424 |
| 4 | ENSP00000267163 ENSP00000251849 ENSP00000309845 ENSP00000361120 |
| 4 | ENSP00000267163 ENSP00000266970 ENSP00000269305 ENSP00000338018 ENSP00000361125 |
| 3 | ENSP00000267163 ENSP00000227507 ENSP00000344818 ENSP00000364133 |
| 14 | ENSP00000267163 ENSP00000251849 ENSP00000302486 ENSP00000366244 |
| 5 | ENSP00000267163 ENSP00000417281 ENSP00000270202 ENSP00000366563 |
| 4 | ENSP00000267163 ENSP00000266970 ENSP00000269305 ENSP00000267868 ENSP00000369497 |
| 12 | ENSP00000267163 ENSP00000417281 ENSP00000270202 ENSP00000348986 ENSP00000375892 |
| 2 | ENSP00000267163 ENSP00000362649 ENSP00000384273 |
| 16 | ENSP00000267868 ENSP00000269305 ENSP00000335153 ENSP00000270202 ENSP00000352121 ENSP00000269300 |
| 1 | ENSP00000267868 ENSP00000269305 |
| 3 | ENSP00000267868 ENSP00000269305 ENSP00000335153 ENSP00000269571 |
| 3 | ENSP00000267868 ENSP00000269305 ENSP00000335153 ENSP00000270202 |
| 18 | ENSP00000267868 ENSP00000269305 ENSP00000268058 ENSP00000254066 ENSP00000284384 ENSP00000342793 ENSP00000005257 ENSP00000019317 ENSP00000272519 |
| 4 | ENSP00000267868 ENSP00000269305 ENSP00000344818 ENSP00000264033 ENSP00000274335 |
| 3 | ENSP00000267868 ENSP00000269305 ENSP00000344818 ENSP00000275493 |
| 4 | ENSP00000267868 ENSP00000269305 ENSP00000335153 ENSP00000251849 ENSP00000288602 |
| 5 | ENSP00000267868 ENSP00000269305 ENSP00000335153 ENSP00000270202 ENSP00000289153 |
| 4 | ENSP00000267868 ENSP00000269305 ENSP00000344818 ENSP00000275493 ENSP00000295400 |
| 4 | ENSP00000267868 ENSP00000269305 ENSP00000335153 ENSP00000251849 ENSP00000302486 |
| 4 | ENSP00000267868 ENSP00000269305 ENSP00000302564 |
| 10 | ENSP00000267868 ENSP00000269305 ENSP00000344818 ENSP00000264033 ENSP00000302269 ENSP00000304283 |
| 3 | ENSP00000267868 ENSP00000269305 ENSP00000329623 ENSP00000309103 |
| 6 | ENSP00000267868 ENSP00000269305 ENSP00000344818 ENSP00000264033 ENSP00000339007 ENSP00000223023 ENSP00000314458 |
| 3 | ENSP00000267868 ENSP00000269305 ENSP00000321410 |
| 4 | ENSP00000267868 ENSP00000269305 ENSP00000344818 ENSP00000347858 ENSP00000330237 |
| 3 | ENSP00000267868 ENSP00000269305 ENSP00000263253 ENSP00000332973 |
| 3 | ENSP00000267868 ENSP00000269305 ENSP00000344818 ENSP00000339151 |
| 3 | ENSP00000267868 ENSP00000269305 ENSP00000263253 ENSP00000341551 |
| 14 | ENSP00000267868 ENSP00000269305 ENSP00000268058 ENSP00000254066 ENSP00000284384 ENSP00000342793 |
| 4 | ENSP00000267868 ENSP00000269305 ENSP00000263253 ENSP00000264657 ENSP00000343204 |
| 3 | ENSP00000267868 ENSP00000269305 ENSP00000329357 ENSP00000345571 |
| 5 | ENSP00000267868 ENSP00000269305 ENSP00000335153 ENSP00000270202 ENSP00000348461 |
| 4 | ENSP00000267868 ENSP00000269305 ENSP00000344818 ENSP00000364133 ENSP00000351905 |
| 5 | ENSP00000267868 ENSP00000269305 ENSP00000335153 ENSP00000270202 ENSP00000352121 |
| 8 | ENSP00000267868 ENSP00000269305 ENSP00000353483 ENSP00000250894 ENSP00000352157 |
| 2 | ENSP00000267868 ENSP00000269305 ENSP00000353483 |
| 3 | ENSP00000267868 ENSP00000269305 ENSP00000262367 ENSP00000354394 |
| 2 | ENSP00000267868 ENSP00000269305 ENSP00000355153 |
| 4 | ENSP00000267868 ENSP00000269305 ENSP00000266970 ENSP00000267163 ENSP00000355249 |
| 7 | ENSP00000267868 ENSP00000269305 ENSP00000344818 ENSP00000364133 ENSP00000351905 ENSP00000355896 |
| 3 | ENSP00000267868 ENSP00000269305 ENSP00000344818 ENSP00000358622 |
| 4 | ENSP00000267868 ENSP00000269305 ENSP00000344818 ENSP00000216797 ENSP00000359424 |
| 5 | ENSP00000267868 ENSP00000269305 ENSP00000335153 ENSP00000251849 ENSP00000309845 ENSP00000361120 |
| 3 | ENSP00000267868 ENSP00000269305 ENSP00000338018 ENSP00000361125 |
| 3 | ENSP00000267868 ENSP00000269305 ENSP00000344818 ENSP00000364133 |
| 15 | ENSP00000267868 ENSP00000269305 ENSP00000335153 ENSP00000251849 ENSP00000302486 ENSP00000366244 |
| 6 | ENSP00000267868 ENSP00000269305 ENSP00000335153 ENSP00000270202 ENSP00000366563 |
| 1 | ENSP00000267868 ENSP00000369497 |
| 12 | ENSP00000267868 ENSP00000269305 ENSP00000344818 ENSP00000264033 ENSP00000274335 ENSP00000303830 ENSP00000348986 ENSP00000375892 |
| 3 | ENSP00000267868 ENSP00000269305 ENSP00000262367 ENSP00000384273 |
| 15 | ENSP00000269300 ENSP00000352121 ENSP00000270202 ENSP00000335153 ENSP00000269305 |
| 15 | ENSP00000269300 ENSP00000352121 ENSP00000270202 ENSP00000335153 ENSP00000269571 |
| 13 | ENSP00000269300 ENSP00000352121 ENSP00000270202 |
| 30 | ENSP00000269300 ENSP00000352121 ENSP00000270202 ENSP00000297494 ENSP00000349467 ENSP00000005257 ENSP00000019317 ENSP00000272519 |
| 16 | ENSP00000269300 ENSP00000352121 ENSP00000270202 ENSP00000263967 ENSP00000274335 |
| 15 | ENSP00000269300 ENSP00000352121 ENSP00000270202 ENSP00000344818 ENSP00000275493 |
| 16 | ENSP00000269300 ENSP00000352121 ENSP00000270202 ENSP00000335153 ENSP00000251849 ENSP00000288602 |
| 15 | ENSP00000269300 ENSP00000352121 ENSP00000270202 ENSP00000289153 |
| 16 | ENSP00000269300 ENSP00000352121 ENSP00000270202 ENSP00000344818 ENSP00000275493 ENSP00000295400 |
| 16 | ENSP00000269300 ENSP00000352121 ENSP00000270202 ENSP00000335153 ENSP00000251849 ENSP00000302486 |
| 16 | ENSP00000269300 ENSP00000352121 ENSP00000270202 ENSP00000309103 ENSP00000302564 |
| 20 | ENSP00000269300 ENSP00000352121 ENSP00000270202 ENSP00000348461 ENSP00000269321 ENSP00000304283 |
| 15 | ENSP00000269300 ENSP00000352121 ENSP00000270202 ENSP00000309103 |
| 17 | ENSP00000269300 ENSP00000352121 ENSP00000270202 ENSP00000348461 ENSP00000268182 ENSP00000314458 |
| 16 | ENSP00000269300 ENSP00000352121 ENSP00000270202 ENSP00000344818 ENSP00000360266 ENSP00000321410 |
| 16 | ENSP00000269300 ENSP00000352121 ENSP00000270202 ENSP00000344818 ENSP00000347858 ENSP00000330237 |
| 15 | ENSP00000269300 ENSP00000352121 ENSP00000270202 ENSP00000344818 ENSP00000332973 |
| 15 | ENSP00000269300 ENSP00000352121 ENSP00000270202 ENSP00000344818 ENSP00000339151 |
| 15 | ENSP00000269300 ENSP00000352121 ENSP00000270202 ENSP00000344818 ENSP00000341551 |
| 26 | ENSP00000269300 ENSP00000352121 ENSP00000270202 ENSP00000348461 ENSP00000298316 ENSP00000342793 |
| 17 | ENSP00000269300 ENSP00000352121 ENSP00000270202 ENSP00000354558 ENSP00000264657 ENSP00000343204 |
| 15 | ENSP00000269300 ENSP00000352121 ENSP00000270202 ENSP00000344818 ENSP00000345571 |
| 15 | ENSP00000269300 ENSP00000352121 ENSP00000270202 ENSP00000348461 |
| 16 | ENSP00000269300 ENSP00000352121 ENSP00000270202 ENSP00000344818 ENSP00000364133 ENSP00000351905 |
| 11 | ENSP00000269300 ENSP00000352121 |
| 22 | ENSP00000269300 ENSP00000352121 ENSP00000270202 ENSP00000335153 ENSP00000269305 ENSP00000353483 ENSP00000250894 ENSP00000352157 |
| 16 | ENSP00000269300 ENSP00000352121 ENSP00000270202 ENSP00000335153 ENSP00000269305 ENSP00000353483 |
| 16 | ENSP00000269300 ENSP00000352121 ENSP00000270202 ENSP00000344818 ENSP00000275493 ENSP00000354394 |
| 15 | ENSP00000269300 ENSP00000352121 ENSP00000270202 ENSP00000417281 ENSP00000355153 |
| 16 | ENSP00000269300 ENSP00000352121 ENSP00000270202 ENSP00000417281 ENSP00000267163 ENSP00000355249 |
| 19 | ENSP00000269300 ENSP00000352121 ENSP00000270202 ENSP00000344818 ENSP00000364133 ENSP00000351905 ENSP00000355896 |
| 15 | ENSP00000269300 ENSP00000352121 ENSP00000270202 ENSP00000344818 ENSP00000358622 |
| 16 | ENSP00000269300 ENSP00000352121 ENSP00000270202 ENSP00000359424 |
| 17 | ENSP00000269300 ENSP00000352121 ENSP00000309845 ENSP00000361120 |
| 16 | ENSP00000269300 ENSP00000352121 ENSP00000270202 ENSP00000335153 ENSP00000338018 ENSP00000361125 |
| 15 | ENSP00000269300 ENSP00000352121 ENSP00000270202 ENSP00000344818 ENSP00000364133 |
| 27 | ENSP00000269300 ENSP00000352121 ENSP00000270202 ENSP00000335153 ENSP00000251849 ENSP00000302486 ENSP00000366244 |
| 16 | ENSP00000269300 ENSP00000352121 ENSP00000270202 ENSP00000366563 |
| 16 | ENSP00000269300 ENSP00000352121 ENSP00000270202 ENSP00000344818 ENSP00000350283 ENSP00000369497 |
| 23 | ENSP00000269300 ENSP00000352121 ENSP00000270202 ENSP00000348986 ENSP00000375892 |
| 15 | ENSP00000269300 ENSP00000352121 ENSP00000270202 ENSP00000344818 ENSP00000384273 |
| 2 | ENSP00000269305 ENSP00000335153 ENSP00000269571 |
| 2 | ENSP00000269305 ENSP00000335153 ENSP00000270202 |
| 17 | ENSP00000269305 ENSP00000268058 ENSP00000254066 ENSP00000284384 ENSP00000342793 ENSP00000005257 ENSP00000019317 ENSP00000272519 |
| 3 | ENSP00000269305 ENSP00000344818 ENSP00000264033 ENSP00000274335 |
| 2 | ENSP00000269305 ENSP00000344818 ENSP00000275493 |
| 3 | ENSP00000269305 ENSP00000335153 ENSP00000251849 ENSP00000288602 |
| 4 | ENSP00000269305 ENSP00000335153 ENSP00000270202 ENSP00000289153 |
| 3 | ENSP00000269305 ENSP00000344818 ENSP00000275493 ENSP00000295400 |
| 3 | ENSP00000269305 ENSP00000335153 ENSP00000251849 ENSP00000302486 |
| 3 | ENSP00000269305 ENSP00000302564 |
| 9 | ENSP00000269305 ENSP00000344818 ENSP00000264033 ENSP00000302269 ENSP00000304283 |
| 2 | ENSP00000269305 ENSP00000329623 ENSP00000309103 |
| 5 | ENSP00000269305 ENSP00000344818 ENSP00000264033 ENSP00000339007 ENSP00000223023 ENSP00000314458 |
| 2 | ENSP00000269305 ENSP00000321410 |
| 3 | ENSP00000269305 ENSP00000344818 ENSP00000347858 ENSP00000330237 |
| 2 | ENSP00000269305 ENSP00000263253 ENSP00000332973 |
| 2 | ENSP00000269305 ENSP00000344818 ENSP00000339151 |
| 2 | ENSP00000269305 ENSP00000263253 ENSP00000341551 |
| 13 | ENSP00000269305 ENSP00000268058 ENSP00000254066 ENSP00000284384 ENSP00000342793 |
| 3 | ENSP00000269305 ENSP00000263253 ENSP00000264657 ENSP00000343204 |
| 2 | ENSP00000269305 ENSP00000329357 ENSP00000345571 |
| 4 | ENSP00000269305 ENSP00000335153 ENSP00000270202 ENSP00000348461 |
| 3 | ENSP00000269305 ENSP00000344818 ENSP00000364133 ENSP00000351905 |
| 4 | ENSP00000269305 ENSP00000335153 ENSP00000270202 ENSP00000352121 |
| 7 | ENSP00000269305 ENSP00000353483 ENSP00000250894 ENSP00000352157 |
| 1 | ENSP00000269305 ENSP00000353483 |
| 2 | ENSP00000269305 ENSP00000262367 ENSP00000354394 |
| 1 | ENSP00000269305 ENSP00000355153 |
| 3 | ENSP00000269305 ENSP00000266970 ENSP00000267163 ENSP00000355249 |
| 6 | ENSP00000269305 ENSP00000344818 ENSP00000364133 ENSP00000351905 ENSP00000355896 |
| 2 | ENSP00000269305 ENSP00000344818 ENSP00000358622 |
| 3 | ENSP00000269305 ENSP00000344818 ENSP00000216797 ENSP00000359424 |
| 4 | ENSP00000269305 ENSP00000335153 ENSP00000251849 ENSP00000309845 ENSP00000361120 |
| 2 | ENSP00000269305 ENSP00000338018 ENSP00000361125 |
| 2 | ENSP00000269305 ENSP00000344818 ENSP00000364133 |
| 14 | ENSP00000269305 ENSP00000335153 ENSP00000251849 ENSP00000302486 ENSP00000366244 |
| 5 | ENSP00000269305 ENSP00000335153 ENSP00000270202 ENSP00000366563 |
| 2 | ENSP00000269305 ENSP00000267868 ENSP00000369497 |
| 11 | ENSP00000269305 ENSP00000344818 ENSP00000264033 ENSP00000274335 ENSP00000303830 ENSP00000348986 ENSP00000375892 |
| 2 | ENSP00000269305 ENSP00000262367 ENSP00000384273 |
| 2 | ENSP00000269571 ENSP00000335153 ENSP00000270202 |
| 17 | ENSP00000269571 ENSP00000350941 ENSP00000282561 ENSP00000284384 ENSP00000342793 ENSP00000005257 ENSP00000019317 ENSP00000272519 |
| 2 | ENSP00000269571 ENSP00000267101 ENSP00000274335 |
| 1 | ENSP00000269571 ENSP00000275493 |
| 3 | ENSP00000269571 ENSP00000335153 ENSP00000251849 ENSP00000288602 |
| 4 | ENSP00000269571 ENSP00000335153 ENSP00000270202 ENSP00000289153 |
| 2 | ENSP00000269571 ENSP00000275493 ENSP00000295400 |
| 3 | ENSP00000269571 ENSP00000335153 ENSP00000251849 ENSP00000302486 |
| 4 | ENSP00000269571 ENSP00000344818 ENSP00000358022 ENSP00000293288 ENSP00000302564 |
| 8 | ENSP00000269571 ENSP00000339007 ENSP00000302269 ENSP00000304283 |
| 4 | ENSP00000269571 ENSP00000335153 ENSP00000270202 ENSP00000309103 |
| 3 | ENSP00000269571 ENSP00000339007 ENSP00000223023 ENSP00000314458 |
| 3 | ENSP00000269571 ENSP00000344818 ENSP00000360266 ENSP00000321410 |
| 3 | ENSP00000269571 ENSP00000344818 ENSP00000347858 ENSP00000330237 |
| 2 | ENSP00000269571 ENSP00000344818 ENSP00000332973 |
| 2 | ENSP00000269571 ENSP00000344818 ENSP00000339151 |
| 2 | ENSP00000269571 ENSP00000344818 ENSP00000341551 |
| 13 | ENSP00000269571 ENSP00000350941 ENSP00000282561 ENSP00000284384 ENSP00000342793 |
| 3 | ENSP00000269571 ENSP00000275493 ENSP00000264657 ENSP00000343204 |
| 2 | ENSP00000269571 ENSP00000344818 ENSP00000345571 |
| 4 | ENSP00000269571 ENSP00000335153 ENSP00000270202 ENSP00000348461 |
| 3 | ENSP00000269571 ENSP00000344818 ENSP00000364133 ENSP00000351905 |
| 4 | ENSP00000269571 ENSP00000335153 ENSP00000270202 ENSP00000352121 |
| 9 | ENSP00000269571 ENSP00000335153 ENSP00000269305 ENSP00000353483 ENSP00000250894 ENSP00000352157 |
| 3 | ENSP00000269571 ENSP00000335153 ENSP00000269305 ENSP00000353483 |
| 2 | ENSP00000269571 ENSP00000275493 ENSP00000354394 |
| 3 | ENSP00000269571 ENSP00000335153 ENSP00000269305 ENSP00000355153 |
| 4 | ENSP00000269571 ENSP00000344818 ENSP00000227507 ENSP00000267163 ENSP00000355249 |
| 6 | ENSP00000269571 ENSP00000344818 ENSP00000364133 ENSP00000351905 ENSP00000355896 |
| 2 | ENSP00000269571 ENSP00000344818 ENSP00000358622 |
| 3 | ENSP00000269571 ENSP00000344818 ENSP00000216797 ENSP00000359424 |
| 4 | ENSP00000269571 ENSP00000335153 ENSP00000251849 ENSP00000309845 ENSP00000361120 |
| 3 | ENSP00000269571 ENSP00000335153 ENSP00000338018 ENSP00000361125 |
| 2 | ENSP00000269571 ENSP00000344818 ENSP00000364133 |
| 14 | ENSP00000269571 ENSP00000335153 ENSP00000251849 ENSP00000302486 ENSP00000366244 |
| 5 | ENSP00000269571 ENSP00000335153 ENSP00000270202 ENSP00000366563 |
| 3 | ENSP00000269571 ENSP00000344818 ENSP00000350283 ENSP00000369497 |
| 10 | ENSP00000269571 ENSP00000267101 ENSP00000274335 ENSP00000303830 ENSP00000348986 ENSP00000375892 |
| 2 | ENSP00000269571 ENSP00000344818 ENSP00000384273 |
| 17 | ENSP00000270202 ENSP00000297494 ENSP00000349467 ENSP00000005257 ENSP00000019317 ENSP00000272519 |
| 3 | ENSP00000270202 ENSP00000263967 ENSP00000274335 |
| 2 | ENSP00000270202 ENSP00000344818 ENSP00000275493 |
| 3 | ENSP00000270202 ENSP00000335153 ENSP00000251849 ENSP00000288602 |
| 2 | ENSP00000270202 ENSP00000289153 |
| 3 | ENSP00000270202 ENSP00000344818 ENSP00000275493 ENSP00000295400 |
| 3 | ENSP00000270202 ENSP00000335153 ENSP00000251849 ENSP00000302486 |
| 3 | ENSP00000270202 ENSP00000309103 ENSP00000302564 |
| 7 | ENSP00000270202 ENSP00000348461 ENSP00000269321 ENSP00000304283 |
| 2 | ENSP00000270202 ENSP00000309103 |
| 4 | ENSP00000270202 ENSP00000348461 ENSP00000268182 ENSP00000314458 |
| 3 | ENSP00000270202 ENSP00000344818 ENSP00000360266 ENSP00000321410 |
| 3 | ENSP00000270202 ENSP00000344818 ENSP00000347858 ENSP00000330237 |
| 2 | ENSP00000270202 ENSP00000344818 ENSP00000332973 |
| 2 | ENSP00000270202 ENSP00000344818 ENSP00000339151 |
| 2 | ENSP00000270202 ENSP00000344818 ENSP00000341551 |
| 13 | ENSP00000270202 ENSP00000348461 ENSP00000298316 ENSP00000342793 |
| 4 | ENSP00000270202 ENSP00000354558 ENSP00000264657 ENSP00000343204 |
| 2 | ENSP00000270202 ENSP00000344818 ENSP00000345571 |
| 2 | ENSP00000270202 ENSP00000348461 |
| 3 | ENSP00000270202 ENSP00000344818 ENSP00000364133 ENSP00000351905 |
| 2 | ENSP00000270202 ENSP00000352121 |
| 9 | ENSP00000270202 ENSP00000335153 ENSP00000269305 ENSP00000353483 ENSP00000250894 ENSP00000352157 |
| 3 | ENSP00000270202 ENSP00000335153 ENSP00000269305 ENSP00000353483 |
| 3 | ENSP00000270202 ENSP00000344818 ENSP00000275493 ENSP00000354394 |
| 2 | ENSP00000270202 ENSP00000417281 ENSP00000355153 |
| 3 | ENSP00000270202 ENSP00000417281 ENSP00000267163 ENSP00000355249 |
| 6 | ENSP00000270202 ENSP00000344818 ENSP00000364133 ENSP00000351905 ENSP00000355896 |
| 2 | ENSP00000270202 ENSP00000344818 ENSP00000358622 |
| 3 | ENSP00000270202 ENSP00000359424 |
| 4 | ENSP00000270202 ENSP00000335153 ENSP00000251849 ENSP00000309845 ENSP00000361120 |
| 3 | ENSP00000270202 ENSP00000335153 ENSP00000338018 ENSP00000361125 |
| 2 | ENSP00000270202 ENSP00000344818 ENSP00000364133 |
| 14 | ENSP00000270202 ENSP00000335153 ENSP00000251849 ENSP00000302486 ENSP00000366244 |
| 3 | ENSP00000270202 ENSP00000366563 |
| 3 | ENSP00000270202 ENSP00000344818 ENSP00000350283 ENSP00000369497 |
| 10 | ENSP00000270202 ENSP00000348986 ENSP00000375892 |
| 2 | ENSP00000270202 ENSP00000344818 ENSP00000384273 |
| 18 | ENSP00000272519 ENSP00000019317 ENSP00000005257 ENSP00000342793 ENSP00000284384 ENSP00000338934 ENSP00000262613 ENSP00000003084 ENSP00000344818 ENSP00000264033 ENSP00000274335 |
| 17 | ENSP00000272519 ENSP00000019317 ENSP00000005257 ENSP00000342793 ENSP00000284384 ENSP00000338934 ENSP00000262613 ENSP00000003084 ENSP00000344818 ENSP00000275493 |
| 19 | ENSP00000272519 ENSP00000019317 ENSP00000005257 ENSP00000349467 ENSP00000297494 ENSP00000335153 ENSP00000251849 ENSP00000288602 |
| 19 | ENSP00000272519 ENSP00000019317 ENSP00000005257 ENSP00000349467 ENSP00000297494 ENSP00000270202 ENSP00000289153 |
| 18 | ENSP00000272519 ENSP00000019317 ENSP00000005257 ENSP00000342793 ENSP00000284384 ENSP00000338934 ENSP00000262613 ENSP00000003084 ENSP00000344818 ENSP00000275493 ENSP00000295400 |
| 19 | ENSP00000272519 ENSP00000019317 ENSP00000005257 ENSP00000342793 ENSP00000284384 ENSP00000338934 ENSP00000262613 ENSP00000003084 ENSP00000344818 ENSP00000360266 ENSP00000215832 ENSP00000302486 |
| 19 | ENSP00000272519 ENSP00000019317 ENSP00000005257 ENSP00000342793 ENSP00000284384 ENSP00000338934 ENSP00000262613 ENSP00000003084 ENSP00000344818 ENSP00000358022 ENSP00000293288 ENSP00000302564 |
| 20 | ENSP00000272519 ENSP00000019317 ENSP00000005257 ENSP00000342793 ENSP00000298316 ENSP00000348461 ENSP00000269321 ENSP00000304283 |
| 19 | ENSP00000272519 ENSP00000019317 ENSP00000005257 ENSP00000349467 ENSP00000297494 ENSP00000270202 ENSP00000309103 |
| 17 | ENSP00000272519 ENSP00000019317 ENSP00000005257 ENSP00000342793 ENSP00000298316 ENSP00000348461 ENSP00000268182 ENSP00000314458 |
| 18 | ENSP00000272519 ENSP00000019317 ENSP00000005257 ENSP00000342793 ENSP00000284384 ENSP00000338934 ENSP00000262613 ENSP00000003084 ENSP00000344818 ENSP00000360266 ENSP00000321410 |
| 18 | ENSP00000272519 ENSP00000019317 ENSP00000005257 ENSP00000342793 ENSP00000284384 ENSP00000338934 ENSP00000262613 ENSP00000003084 ENSP00000344818 ENSP00000347858 ENSP00000330237 |
| 17 | ENSP00000272519 ENSP00000019317 ENSP00000005257 ENSP00000342793 ENSP00000284384 ENSP00000338934 ENSP00000262613 ENSP00000003084 ENSP00000344818 ENSP00000332973 |
| 17 | ENSP00000272519 ENSP00000019317 ENSP00000005257 ENSP00000342793 ENSP00000284384 ENSP00000338934 ENSP00000262613 ENSP00000003084 ENSP00000344818 ENSP00000339151 |
| 17 | ENSP00000272519 ENSP00000019317 ENSP00000005257 ENSP00000342793 ENSP00000284384 ENSP00000338934 ENSP00000262613 ENSP00000003084 ENSP00000344818 ENSP00000341551 |
| 4 | ENSP00000272519 ENSP00000019317 ENSP00000005257 ENSP00000342793 |
| 18 | ENSP00000272519 ENSP00000019317 ENSP00000005257 ENSP00000342793 ENSP00000284384 ENSP00000282561 ENSP00000350941 ENSP00000264657 ENSP00000343204 |
| 17 | ENSP00000272519 ENSP00000019317 ENSP00000005257 ENSP00000342793 ENSP00000284384 ENSP00000338934 ENSP00000262613 ENSP00000003084 ENSP00000344818 ENSP00000345571 |
| 15 | ENSP00000272519 ENSP00000019317 ENSP00000005257 ENSP00000342793 ENSP00000298316 ENSP00000348461 |
| 18 | ENSP00000272519 ENSP00000019317 ENSP00000005257 ENSP00000342793 ENSP00000284384 ENSP00000338934 ENSP00000262613 ENSP00000003084 ENSP00000344818 ENSP00000364133 ENSP00000351905 |
| 19 | ENSP00000272519 ENSP00000019317 ENSP00000005257 ENSP00000349467 ENSP00000297494 ENSP00000270202 ENSP00000352121 |
| 24 | ENSP00000272519 ENSP00000019317 ENSP00000005257 ENSP00000342793 ENSP00000284384 ENSP00000254066 ENSP00000268058 ENSP00000269305 ENSP00000353483 ENSP00000250894 ENSP00000352157 |
| 18 | ENSP00000272519 ENSP00000019317 ENSP00000005257 ENSP00000342793 ENSP00000284384 ENSP00000254066 ENSP00000268058 ENSP00000269305 ENSP00000353483 |
| 18 | ENSP00000272519 ENSP00000019317 ENSP00000005257 ENSP00000342793 ENSP00000284384 ENSP00000254066 ENSP00000320940 ENSP00000262367 ENSP00000354394 |
| 18 | ENSP00000272519 ENSP00000019317 ENSP00000005257 ENSP00000342793 ENSP00000284384 ENSP00000254066 ENSP00000268058 ENSP00000269305 ENSP00000355153 |
| 19 | ENSP00000272519 ENSP00000019317 ENSP00000005257 ENSP00000342793 ENSP00000284384 ENSP00000338934 ENSP00000262613 ENSP00000003084 ENSP00000344818 ENSP00000227507 ENSP00000267163 ENSP00000355249 |
| 21 | ENSP00000272519 ENSP00000019317 ENSP00000005257 ENSP00000342793 ENSP00000284384 ENSP00000338934 ENSP00000262613 ENSP00000003084 ENSP00000344818 ENSP00000364133 ENSP00000351905 ENSP00000355896 |
| 17 | ENSP00000272519 ENSP00000019317 ENSP00000005257 ENSP00000342793 ENSP00000284384 ENSP00000338934 ENSP00000262613 ENSP00000003084 ENSP00000344818 ENSP00000358622 |
| 18 | ENSP00000272519 ENSP00000019317 ENSP00000005257 ENSP00000342793 ENSP00000284384 ENSP00000338934 ENSP00000262613 ENSP00000003084 ENSP00000344818 ENSP00000216797 ENSP00000359424 |
| 20 | ENSP00000272519 ENSP00000019317 ENSP00000005257 ENSP00000342793 ENSP00000284384 ENSP00000282561 ENSP00000350941 ENSP00000309845 ENSP00000361120 |
| 18 | ENSP00000272519 ENSP00000019317 ENSP00000005257 ENSP00000342793 ENSP00000284384 ENSP00000338934 ENSP00000262613 ENSP00000003084 ENSP00000344818 ENSP00000338018 ENSP00000361125 |
| 17 | ENSP00000272519 ENSP00000019317 ENSP00000005257 ENSP00000342793 ENSP00000284384 ENSP00000338934 ENSP00000262613 ENSP00000003084 ENSP00000344818 ENSP00000364133 |
| 30 | ENSP00000272519 ENSP00000019317 ENSP00000005257 ENSP00000342793 ENSP00000284384 ENSP00000338934 ENSP00000262613 ENSP00000003084 ENSP00000344818 ENSP00000360266 ENSP00000215832 ENSP00000302486 ENSP00000366244 |
| 20 | ENSP00000272519 ENSP00000019317 ENSP00000005257 ENSP00000349467 ENSP00000297494 ENSP00000270202 ENSP00000366563 |
| 18 | ENSP00000272519 ENSP00000019317 ENSP00000005257 ENSP00000342793 ENSP00000284384 ENSP00000338934 ENSP00000262613 ENSP00000003084 ENSP00000344818 ENSP00000350283 ENSP00000369497 |
| 25 | ENSP00000272519 ENSP00000019317 ENSP00000005257 ENSP00000342793 ENSP00000284384 ENSP00000282561 ENSP00000350941 ENSP00000360683 ENSP00000303830 ENSP00000348986 ENSP00000375892 |
| 17 | ENSP00000272519 ENSP00000019317 ENSP00000005257 ENSP00000342793 ENSP00000284384 ENSP00000338934 ENSP00000262613 ENSP00000003084 ENSP00000344818 ENSP00000384273 |
| 2 | ENSP00000274335 ENSP00000264033 ENSP00000275493 |
| 4 | ENSP00000274335 ENSP00000263967 ENSP00000309845 ENSP00000288602 |
| 5 | ENSP00000274335 ENSP00000289153 |
| 3 | ENSP00000274335 ENSP00000264033 ENSP00000275493 ENSP00000295400 |
| 4 | ENSP00000274335 ENSP00000263967 ENSP00000309845 ENSP00000251849 ENSP00000302486 |
| 5 | ENSP00000274335 ENSP00000264033 ENSP00000344818 ENSP00000358022 ENSP00000293288 ENSP00000302564 |
| 8 | ENSP00000274335 ENSP00000264033 ENSP00000302269 ENSP00000304283 |
| 5 | ENSP00000274335 ENSP00000263967 ENSP00000270202 ENSP00000309103 |
| 3 | ENSP00000274335 ENSP00000339007 ENSP00000223023 ENSP00000314458 |
| 4 | ENSP00000274335 ENSP00000264033 ENSP00000344818 ENSP00000360266 ENSP00000321410 |
| 4 | ENSP00000274335 ENSP00000264033 ENSP00000344818 ENSP00000347858 ENSP00000330237 |
| 3 | ENSP00000274335 ENSP00000264033 ENSP00000344818 ENSP00000332973 |
| 3 | ENSP00000274335 ENSP00000264033 ENSP00000344818 ENSP00000339151 |
| 3 | ENSP00000274335 ENSP00000264033 ENSP00000344818 ENSP00000341551 |
| 14 | ENSP00000274335 ENSP00000264033 ENSP00000350941 ENSP00000282561 ENSP00000284384 ENSP00000342793 |
| 2 | ENSP00000274335 ENSP00000304895 ENSP00000343204 |
| 3 | ENSP00000274335 ENSP00000264033 ENSP00000344818 ENSP00000345571 |
| 4 | ENSP00000274335 ENSP00000264033 ENSP00000302269 ENSP00000348461 |
| 4 | ENSP00000274335 ENSP00000264033 ENSP00000344818 ENSP00000364133 ENSP00000351905 |
| 5 | ENSP00000274335 ENSP00000263967 ENSP00000270202 ENSP00000352121 |
| 9 | ENSP00000274335 ENSP00000304895 ENSP00000353483 ENSP00000250894 ENSP00000352157 |
| 3 | ENSP00000274335 ENSP00000304895 ENSP00000353483 |
| 3 | ENSP00000274335 ENSP00000264033 ENSP00000275493 ENSP00000354394 |
| 4 | ENSP00000274335 ENSP00000264033 ENSP00000344818 ENSP00000269305 ENSP00000355153 |
| 5 | ENSP00000274335 ENSP00000264033 ENSP00000361423 ENSP00000267163 ENSP00000355249 |
| 7 | ENSP00000274335 ENSP00000264033 ENSP00000344818 ENSP00000364133 ENSP00000351905 ENSP00000355896 |
| 3 | ENSP00000274335 ENSP00000264033 ENSP00000344818 ENSP00000358622 |
| 4 | ENSP00000274335 ENSP00000264033 ENSP00000344818 ENSP00000216797 ENSP00000359424 |
| 3 | ENSP00000274335 ENSP00000263967 ENSP00000309845 ENSP00000361120 |
| 4 | ENSP00000274335 ENSP00000264033 ENSP00000344818 ENSP00000338018 ENSP00000361125 |
| 3 | ENSP00000274335 ENSP00000264033 ENSP00000344818 ENSP00000364133 |
| 15 | ENSP00000274335 ENSP00000263967 ENSP00000309845 ENSP00000251849 ENSP00000302486 ENSP00000366244 |
| 6 | ENSP00000274335 ENSP00000263967 ENSP00000270202 ENSP00000366563 |
| 4 | ENSP00000274335 ENSP00000264033 ENSP00000344818 ENSP00000350283 ENSP00000369497 |
| 8 | ENSP00000274335 ENSP00000303830 ENSP00000348986 ENSP00000375892 |
| 3 | ENSP00000274335 ENSP00000264033 ENSP00000344818 ENSP00000384273 |
| 4 | ENSP00000275493 ENSP00000269571 ENSP00000335153 ENSP00000251849 ENSP00000288602 |
| 4 | ENSP00000275493 ENSP00000344818 ENSP00000270202 ENSP00000289153 |
| 1 | ENSP00000275493 ENSP00000295400 |
| 4 | ENSP00000275493 ENSP00000344818 ENSP00000360266 ENSP00000215832 ENSP00000302486 |
| 4 | ENSP00000275493 ENSP00000344818 ENSP00000358022 ENSP00000293288 ENSP00000302564 |
| 8 | ENSP00000275493 ENSP00000264033 ENSP00000302269 ENSP00000304283 |
| 4 | ENSP00000275493 ENSP00000344818 ENSP00000270202 ENSP00000309103 |
| 3 | ENSP00000275493 ENSP00000339007 ENSP00000223023 ENSP00000314458 |
| 3 | ENSP00000275493 ENSP00000344818 ENSP00000360266 ENSP00000321410 |
| 3 | ENSP00000275493 ENSP00000344818 ENSP00000347858 ENSP00000330237 |
| 2 | ENSP00000275493 ENSP00000344818 ENSP00000332973 |
| 2 | ENSP00000275493 ENSP00000344818 ENSP00000339151 |
| 2 | ENSP00000275493 ENSP00000344818 ENSP00000341551 |
| 13 | ENSP00000275493 ENSP00000350941 ENSP00000282561 ENSP00000284384 ENSP00000342793 |
| 2 | ENSP00000275493 ENSP00000264657 ENSP00000343204 |
| 2 | ENSP00000275493 ENSP00000344818 ENSP00000345571 |
| 4 | ENSP00000275493 ENSP00000344818 ENSP00000270202 ENSP00000348461 |
| 3 | ENSP00000275493 ENSP00000344818 ENSP00000364133 ENSP00000351905 |
| 4 | ENSP00000275493 ENSP00000344818 ENSP00000270202 ENSP00000352121 |
| 9 | ENSP00000275493 ENSP00000344818 ENSP00000269305 ENSP00000353483 ENSP00000250894 ENSP00000352157 |
| 3 | ENSP00000275493 ENSP00000344818 ENSP00000269305 ENSP00000353483 |
| 1 | ENSP00000275493 ENSP00000354394 |
| 3 | ENSP00000275493 ENSP00000344818 ENSP00000269305 ENSP00000355153 |
| 4 | ENSP00000275493 ENSP00000264657 ENSP00000227507 ENSP00000267163 ENSP00000355249 |
| 6 | ENSP00000275493 ENSP00000344818 ENSP00000364133 ENSP00000351905 ENSP00000355896 |
| 2 | ENSP00000275493 ENSP00000344818 ENSP00000358622 |
| 3 | ENSP00000275493 ENSP00000344818 ENSP00000216797 ENSP00000359424 |
| 4 | ENSP00000275493 ENSP00000384675 ENSP00000309845 ENSP00000361120 |
| 3 | ENSP00000275493 ENSP00000344818 ENSP00000338018 ENSP00000361125 |
| 2 | ENSP00000275493 ENSP00000344818 ENSP00000364133 |
| 15 | ENSP00000275493 ENSP00000344818 ENSP00000360266 ENSP00000215832 ENSP00000302486 ENSP00000366244 |
| 5 | ENSP00000275493 ENSP00000344818 ENSP00000270202 ENSP00000366563 |
| 3 | ENSP00000275493 ENSP00000344818 ENSP00000350283 ENSP00000369497 |
| 9 | ENSP00000275493 ENSP00000340944 ENSP00000303830 ENSP00000348986 ENSP00000375892 |
| 2 | ENSP00000275493 ENSP00000344818 ENSP00000384273 |
| 5 | ENSP00000288602 ENSP00000251849 ENSP00000335153 ENSP00000270202 ENSP00000289153 |
| 5 | ENSP00000288602 ENSP00000251849 ENSP00000335153 ENSP00000269571 ENSP00000275493 ENSP00000295400 |
| 2 | ENSP00000288602 ENSP00000251849 ENSP00000302486 |
| 4 | ENSP00000288602 ENSP00000251849 ENSP00000309503 ENSP00000309103 ENSP00000302564 |
| 10 | ENSP00000288602 ENSP00000251849 ENSP00000335153 ENSP00000270202 ENSP00000348461 ENSP00000269321 ENSP00000304283 |
| 3 | ENSP00000288602 ENSP00000251849 ENSP00000309503 ENSP00000309103 |
| 6 | ENSP00000288602 ENSP00000251849 ENSP00000335153 ENSP00000269571 ENSP00000339007 ENSP00000223023 ENSP00000314458 |
| 5 | ENSP00000288602 ENSP00000251849 ENSP00000335153 ENSP00000269305 ENSP00000321410 |
| 6 | ENSP00000288602 ENSP00000251849 ENSP00000335153 ENSP00000206249 ENSP00000344818 ENSP00000347858 ENSP00000330237 |
| 5 | ENSP00000288602 ENSP00000251849 ENSP00000335153 ENSP00000206249 ENSP00000263253 ENSP00000332973 |
| 5 | ENSP00000288602 ENSP00000251849 ENSP00000335153 ENSP00000206249 ENSP00000344818 ENSP00000339151 |
| 5 | ENSP00000288602 ENSP00000251849 ENSP00000335153 ENSP00000206249 ENSP00000263253 ENSP00000341551 |
| 16 | ENSP00000288602 ENSP00000251849 ENSP00000335153 ENSP00000270202 ENSP00000348461 ENSP00000298316 ENSP00000342793 |
| 5 | ENSP00000288602 ENSP00000309845 ENSP00000263967 ENSP00000304895 ENSP00000343204 |
| 4 | ENSP00000288602 ENSP00000251849 ENSP00000267163 ENSP00000345571 |
| 5 | ENSP00000288602 ENSP00000251849 ENSP00000335153 ENSP00000270202 ENSP00000348461 |
| 6 | ENSP00000288602 ENSP00000251849 ENSP00000335153 ENSP00000206249 ENSP00000344818 ENSP00000364133 ENSP00000351905 |
| 5 | ENSP00000288602 ENSP00000251849 ENSP00000335153 ENSP00000270202 ENSP00000352121 |
| 10 | ENSP00000288602 ENSP00000251849 ENSP00000335153 ENSP00000269305 ENSP00000353483 ENSP00000250894 ENSP00000352157 |
| 4 | ENSP00000288602 ENSP00000251849 ENSP00000335153 ENSP00000269305 ENSP00000353483 |
| 5 | ENSP00000288602 ENSP00000251849 ENSP00000335153 ENSP00000206249 ENSP00000262367 ENSP00000354394 |
| 4 | ENSP00000288602 ENSP00000251849 ENSP00000335153 ENSP00000269305 ENSP00000355153 |
| 4 | ENSP00000288602 ENSP00000251849 ENSP00000267163 ENSP00000355249 |
| 9 | ENSP00000288602 ENSP00000251849 ENSP00000335153 ENSP00000206249 ENSP00000344818 ENSP00000364133 ENSP00000351905 ENSP00000355896 |
| 5 | ENSP00000288602 ENSP00000251849 ENSP00000335153 ENSP00000206249 ENSP00000344818 ENSP00000358622 |
| 6 | ENSP00000288602 ENSP00000251849 ENSP00000335153 ENSP00000270202 ENSP00000359424 |
| 3 | ENSP00000288602 ENSP00000309845 ENSP00000361120 |
| 4 | ENSP00000288602 ENSP00000251849 ENSP00000335153 ENSP00000338018 ENSP00000361125 |
| 5 | ENSP00000288602 ENSP00000251849 ENSP00000335153 ENSP00000206249 ENSP00000344818 ENSP00000364133 |
| 13 | ENSP00000288602 ENSP00000251849 ENSP00000302486 ENSP00000366244 |
| 6 | ENSP00000288602 ENSP00000251849 ENSP00000335153 ENSP00000270202 ENSP00000366563 |
| 5 | ENSP00000288602 ENSP00000251849 ENSP00000335153 ENSP00000269305 ENSP00000267868 ENSP00000369497 |
| 12 | ENSP00000288602 ENSP00000309845 ENSP00000263967 ENSP00000274335 ENSP00000303830 ENSP00000348986 ENSP00000375892 |
| 5 | ENSP00000288602 ENSP00000251849 ENSP00000335153 ENSP00000206249 ENSP00000262367 ENSP00000384273 |
| 5 | ENSP00000289153 ENSP00000270202 ENSP00000344818 ENSP00000275493 ENSP00000295400 |
| 5 | ENSP00000289153 ENSP00000270202 ENSP00000335153 ENSP00000251849 ENSP00000302486 |
| 5 | ENSP00000289153 ENSP00000270202 ENSP00000309103 ENSP00000302564 |
| 9 | ENSP00000289153 ENSP00000270202 ENSP00000348461 ENSP00000269321 ENSP00000304283 |
| 4 | ENSP00000289153 ENSP00000270202 ENSP00000309103 |
| 6 | ENSP00000289153 ENSP00000270202 ENSP00000348461 ENSP00000268182 ENSP00000314458 |
| 5 | ENSP00000289153 ENSP00000270202 ENSP00000344818 ENSP00000360266 ENSP00000321410 |
| 5 | ENSP00000289153 ENSP00000270202 ENSP00000344818 ENSP00000347858 ENSP00000330237 |
| 4 | ENSP00000289153 ENSP00000270202 ENSP00000344818 ENSP00000332973 |
| 4 | ENSP00000289153 ENSP00000270202 ENSP00000344818 ENSP00000339151 |
| 4 | ENSP00000289153 ENSP00000270202 ENSP00000344818 ENSP00000341551 |
| 15 | ENSP00000289153 ENSP00000270202 ENSP00000348461 ENSP00000298316 ENSP00000342793 |
| 6 | ENSP00000289153 ENSP00000270202 ENSP00000354558 ENSP00000264657 ENSP00000343204 |
| 4 | ENSP00000289153 ENSP00000270202 ENSP00000344818 ENSP00000345571 |
| 4 | ENSP00000289153 ENSP00000270202 ENSP00000348461 |
| 5 | ENSP00000289153 ENSP00000270202 ENSP00000344818 ENSP00000364133 ENSP00000351905 |
| 4 | ENSP00000289153 ENSP00000270202 ENSP00000352121 |
| 11 | ENSP00000289153 ENSP00000270202 ENSP00000335153 ENSP00000269305 ENSP00000353483 ENSP00000250894 ENSP00000352157 |
| 5 | ENSP00000289153 ENSP00000270202 ENSP00000335153 ENSP00000269305 ENSP00000353483 |
| 5 | ENSP00000289153 ENSP00000270202 ENSP00000344818 ENSP00000275493 ENSP00000354394 |
| 4 | ENSP00000289153 ENSP00000270202 ENSP00000417281 ENSP00000355153 |
| 5 | ENSP00000289153 ENSP00000270202 ENSP00000417281 ENSP00000267163 ENSP00000355249 |
| 8 | ENSP00000289153 ENSP00000270202 ENSP00000344818 ENSP00000364133 ENSP00000351905 ENSP00000355896 |
| 4 | ENSP00000289153 ENSP00000270202 ENSP00000344818 ENSP00000358622 |
| 5 | ENSP00000289153 ENSP00000270202 ENSP00000359424 |
| 6 | ENSP00000289153 ENSP00000270202 ENSP00000335153 ENSP00000251849 ENSP00000309845 ENSP00000361120 |
| 5 | ENSP00000289153 ENSP00000270202 ENSP00000335153 ENSP00000338018 ENSP00000361125 |
| 4 | ENSP00000289153 ENSP00000270202 ENSP00000344818 ENSP00000364133 |
| 16 | ENSP00000289153 ENSP00000270202 ENSP00000335153 ENSP00000251849 ENSP00000302486 ENSP00000366244 |
| 5 | ENSP00000289153 ENSP00000270202 ENSP00000366563 |
| 5 | ENSP00000289153 ENSP00000270202 ENSP00000344818 ENSP00000350283 ENSP00000369497 |
| 12 | ENSP00000289153 ENSP00000270202 ENSP00000348986 ENSP00000375892 |
| 4 | ENSP00000289153 ENSP00000270202 ENSP00000344818 ENSP00000384273 |
| 5 | ENSP00000295400 ENSP00000275493 ENSP00000344818 ENSP00000360266 ENSP00000215832 ENSP00000302486 |
| 5 | ENSP00000295400 ENSP00000275493 ENSP00000344818 ENSP00000358022 ENSP00000293288 ENSP00000302564 |
| 9 | ENSP00000295400 ENSP00000275493 ENSP00000264033 ENSP00000302269 ENSP00000304283 |
| 5 | ENSP00000295400 ENSP00000275493 ENSP00000344818 ENSP00000270202 ENSP00000309103 |
| 4 | ENSP00000295400 ENSP00000275493 ENSP00000339007 ENSP00000223023 ENSP00000314458 |
| 4 | ENSP00000295400 ENSP00000275493 ENSP00000344818 ENSP00000360266 ENSP00000321410 |
| 4 | ENSP00000295400 ENSP00000275493 ENSP00000344818 ENSP00000347858 ENSP00000330237 |
| 3 | ENSP00000295400 ENSP00000275493 ENSP00000344818 ENSP00000332973 |
| 3 | ENSP00000295400 ENSP00000275493 ENSP00000344818 ENSP00000339151 |
| 3 | ENSP00000295400 ENSP00000275493 ENSP00000344818 ENSP00000341551 |
| 14 | ENSP00000295400 ENSP00000275493 ENSP00000350941 ENSP00000282561 ENSP00000284384 ENSP00000342793 |
| 3 | ENSP00000295400 ENSP00000275493 ENSP00000264657 ENSP00000343204 |
| 3 | ENSP00000295400 ENSP00000275493 ENSP00000344818 ENSP00000345571 |
| 5 | ENSP00000295400 ENSP00000275493 ENSP00000344818 ENSP00000270202 ENSP00000348461 |
| 4 | ENSP00000295400 ENSP00000275493 ENSP00000344818 ENSP00000364133 ENSP00000351905 |
| 5 | ENSP00000295400 ENSP00000275493 ENSP00000344818 ENSP00000270202 ENSP00000352121 |
| 10 | ENSP00000295400 ENSP00000275493 ENSP00000344818 ENSP00000269305 ENSP00000353483 ENSP00000250894 ENSP00000352157 |
| 4 | ENSP00000295400 ENSP00000275493 ENSP00000344818 ENSP00000269305 ENSP00000353483 |
| 2 | ENSP00000295400 ENSP00000275493 ENSP00000354394 |
| 4 | ENSP00000295400 ENSP00000275493 ENSP00000344818 ENSP00000269305 ENSP00000355153 |
| 5 | ENSP00000295400 ENSP00000275493 ENSP00000264657 ENSP00000227507 ENSP00000267163 ENSP00000355249 |
| 7 | ENSP00000295400 ENSP00000275493 ENSP00000344818 ENSP00000364133 ENSP00000351905 ENSP00000355896 |
| 3 | ENSP00000295400 ENSP00000275493 ENSP00000344818 ENSP00000358622 |
| 4 | ENSP00000295400 ENSP00000275493 ENSP00000344818 ENSP00000216797 ENSP00000359424 |
| 5 | ENSP00000295400 ENSP00000275493 ENSP00000384675 ENSP00000309845 ENSP00000361120 |
| 4 | ENSP00000295400 ENSP00000275493 ENSP00000344818 ENSP00000338018 ENSP00000361125 |
| 3 | ENSP00000295400 ENSP00000275493 ENSP00000344818 ENSP00000364133 |
| 16 | ENSP00000295400 ENSP00000275493 ENSP00000344818 ENSP00000360266 ENSP00000215832 ENSP00000302486 ENSP00000366244 |
| 6 | ENSP00000295400 ENSP00000275493 ENSP00000344818 ENSP00000270202 ENSP00000366563 |
| 4 | ENSP00000295400 ENSP00000275493 ENSP00000344818 ENSP00000350283 ENSP00000369497 |
| 10 | ENSP00000295400 ENSP00000275493 ENSP00000340944 ENSP00000303830 ENSP00000348986 ENSP00000375892 |
| 3 | ENSP00000295400 ENSP00000275493 ENSP00000344818 ENSP00000384273 |
| 4 | ENSP00000302486 ENSP00000251849 ENSP00000309503 ENSP00000309103 ENSP00000302564 |
| 10 | ENSP00000302486 ENSP00000251849 ENSP00000335153 ENSP00000270202 ENSP00000348461 ENSP00000269321 ENSP00000304283 |
| 3 | ENSP00000302486 ENSP00000251849 ENSP00000309503 ENSP00000309103 |
| 6 | ENSP00000302486 ENSP00000251849 ENSP00000335153 ENSP00000269571 ENSP00000339007 ENSP00000223023 ENSP00000314458 |
| 3 | ENSP00000302486 ENSP00000215832 ENSP00000360266 ENSP00000321410 |
| 5 | ENSP00000302486 ENSP00000215832 ENSP00000360266 ENSP00000344818 ENSP00000347858 ENSP00000330237 |
| 4 | ENSP00000302486 ENSP00000215832 ENSP00000360266 ENSP00000332973 |
| 4 | ENSP00000302486 ENSP00000215832 ENSP00000360266 ENSP00000344818 ENSP00000339151 |
| 4 | ENSP00000302486 ENSP00000215832 ENSP00000360266 ENSP00000263253 ENSP00000341551 |
| 15 | ENSP00000302486 ENSP00000215832 ENSP00000360266 ENSP00000344818 ENSP00000003084 ENSP00000262613 ENSP00000338934 ENSP00000284384 ENSP00000342793 |
| 5 | ENSP00000302486 ENSP00000215832 ENSP00000360266 ENSP00000263253 ENSP00000264657 ENSP00000343204 |
| 4 | ENSP00000302486 ENSP00000251849 ENSP00000267163 ENSP00000345571 |
| 5 | ENSP00000302486 ENSP00000251849 ENSP00000335153 ENSP00000270202 ENSP00000348461 |
| 5 | ENSP00000302486 ENSP00000215832 ENSP00000360266 ENSP00000344818 ENSP00000364133 ENSP00000351905 |
| 5 | ENSP00000302486 ENSP00000251849 ENSP00000335153 ENSP00000270202 ENSP00000352121 |
| 9 | ENSP00000302486 ENSP00000215832 ENSP00000360266 ENSP00000353483 ENSP00000250894 ENSP00000352157 |
| 3 | ENSP00000302486 ENSP00000215832 ENSP00000360266 ENSP00000353483 |
| 4 | ENSP00000302486 ENSP00000215832 ENSP00000360266 ENSP00000263253 ENSP00000354394 |
| 4 | ENSP00000302486 ENSP00000251849 ENSP00000335153 ENSP00000269305 ENSP00000355153 |
| 4 | ENSP00000302486 ENSP00000251849 ENSP00000267163 ENSP00000355249 |
| 8 | ENSP00000302486 ENSP00000215832 ENSP00000360266 ENSP00000344818 ENSP00000364133 ENSP00000351905 ENSP00000355896 |
| 4 | ENSP00000302486 ENSP00000215832 ENSP00000360266 ENSP00000344818 ENSP00000358622 |
| 5 | ENSP00000302486 ENSP00000215832 ENSP00000360266 ENSP00000344818 ENSP00000216797 ENSP00000359424 |
| 3 | ENSP00000302486 ENSP00000251849 ENSP00000309845 ENSP00000361120 |
| 4 | ENSP00000302486 ENSP00000251849 ENSP00000335153 ENSP00000338018 ENSP00000361125 |
| 4 | ENSP00000302486 ENSP00000215832 ENSP00000360266 ENSP00000344818 ENSP00000364133 |
| 11 | ENSP00000302486 ENSP00000366244 |
| 6 | ENSP00000302486 ENSP00000251849 ENSP00000335153 ENSP00000270202 ENSP00000366563 |
| 5 | ENSP00000302486 ENSP00000251849 ENSP00000335153 ENSP00000269305 ENSP00000267868 ENSP00000369497 |
| 12 | ENSP00000302486 ENSP00000251849 ENSP00000309845 ENSP00000263967 ENSP00000274335 ENSP00000303830 ENSP00000348986 ENSP00000375892 |
| 4 | ENSP00000302486 ENSP00000215832 ENSP00000360266 ENSP00000263253 ENSP00000384273 |
| 10 | ENSP00000302564 ENSP00000309103 ENSP00000270202 ENSP00000348461 ENSP00000269321 ENSP00000304283 |
| 1 | ENSP00000302564 ENSP00000309103 |
| 7 | ENSP00000302564 ENSP00000293288 ENSP00000358022 ENSP00000344818 ENSP00000264033 ENSP00000339007 ENSP00000223023 ENSP00000314458 |
| 5 | ENSP00000302564 ENSP00000269305 ENSP00000321410 |
| 5 | ENSP00000302564 ENSP00000293288 ENSP00000358022 ENSP00000344818 ENSP00000347858 ENSP00000330237 |
| 4 | ENSP00000302564 ENSP00000293288 ENSP00000358022 ENSP00000344818 ENSP00000332973 |
| 4 | ENSP00000302564 ENSP00000293288 ENSP00000358022 ENSP00000344818 ENSP00000339151 |
| 4 | ENSP00000302564 ENSP00000293288 ENSP00000358022 ENSP00000344818 ENSP00000341551 |
| 15 | ENSP00000302564 ENSP00000293288 ENSP00000358022 ENSP00000344818 ENSP00000003084 ENSP00000262613 ENSP00000338934 ENSP00000284384 ENSP00000342793 |
| 6 | ENSP00000302564 ENSP00000293288 ENSP00000358022 ENSP00000344818 ENSP00000227507 ENSP00000264657 ENSP00000343204 |
| 4 | ENSP00000302564 ENSP00000293288 ENSP00000358022 ENSP00000344818 ENSP00000345571 |
| 5 | ENSP00000302564 ENSP00000309103 ENSP00000270202 ENSP00000348461 |
| 5 | ENSP00000302564 ENSP00000293288 ENSP00000358022 ENSP00000344818 ENSP00000364133 ENSP00000351905 |
| 5 | ENSP00000302564 ENSP00000309103 ENSP00000270202 ENSP00000352121 |
| 10 | ENSP00000302564 ENSP00000269305 ENSP00000353483 ENSP00000250894 ENSP00000352157 |
| 4 | ENSP00000302564 ENSP00000269305 ENSP00000353483 |
| 5 | ENSP00000302564 ENSP00000269305 ENSP00000262367 ENSP00000354394 |
| 4 | ENSP00000302564 ENSP00000269305 ENSP00000355153 |
| 6 | ENSP00000302564 ENSP00000309103 ENSP00000309503 ENSP00000251849 ENSP00000267163 ENSP00000355249 |
| 8 | ENSP00000302564 ENSP00000293288 ENSP00000358022 ENSP00000344818 ENSP00000364133 ENSP00000351905 ENSP00000355896 |
| 4 | ENSP00000302564 ENSP00000293288 ENSP00000358022 ENSP00000344818 ENSP00000358622 |
| 5 | ENSP00000302564 ENSP00000293288 ENSP00000358022 ENSP00000344818 ENSP00000216797 ENSP00000359424 |
| 5 | ENSP00000302564 ENSP00000309103 ENSP00000309503 ENSP00000251849 ENSP00000309845 ENSP00000361120 |
| 5 | ENSP00000302564 ENSP00000269305 ENSP00000338018 ENSP00000361125 |
| 4 | ENSP00000302564 ENSP00000293288 ENSP00000358022 ENSP00000344818 ENSP00000364133 |
| 15 | ENSP00000302564 ENSP00000309103 ENSP00000309503 ENSP00000251849 ENSP00000302486 ENSP00000366244 |
| 6 | ENSP00000302564 ENSP00000309103 ENSP00000270202 ENSP00000366563 |
| 5 | ENSP00000302564 ENSP00000269305 ENSP00000267868 ENSP00000369497 |
| 13 | ENSP00000302564 ENSP00000309103 ENSP00000270202 ENSP00000348986 ENSP00000375892 |
| 4 | ENSP00000302564 ENSP00000293288 ENSP00000358022 ENSP00000344818 ENSP00000384273 |
| 9 | ENSP00000304283 ENSP00000269321 ENSP00000348461 ENSP00000270202 ENSP00000309103 |
| 5 | ENSP00000304283 ENSP00000269321 ENSP00000314458 |
| 10 | ENSP00000304283 ENSP00000302269 ENSP00000264033 ENSP00000344818 ENSP00000360266 ENSP00000321410 |
| 10 | ENSP00000304283 ENSP00000302269 ENSP00000264033 ENSP00000344818 ENSP00000347858 ENSP00000330237 |
| 9 | ENSP00000304283 ENSP00000302269 ENSP00000264033 ENSP00000344818 ENSP00000332973 |
| 9 | ENSP00000304283 ENSP00000302269 ENSP00000264033 ENSP00000344818 ENSP00000339151 |
| 9 | ENSP00000304283 ENSP00000302269 ENSP00000264033 ENSP00000344818 ENSP00000341551 |
| 16 | ENSP00000304283 ENSP00000269321 ENSP00000348461 ENSP00000298316 ENSP00000342793 |
| 9 | ENSP00000304283 ENSP00000269321 ENSP00000314458 ENSP00000223023 ENSP00000339007 ENSP00000304895 ENSP00000343204 |
| 9 | ENSP00000304283 ENSP00000302269 ENSP00000264033 ENSP00000344818 ENSP00000345571 |
| 5 | ENSP00000304283 ENSP00000269321 ENSP00000348461 |
| 10 | ENSP00000304283 ENSP00000302269 ENSP00000264033 ENSP00000344818 ENSP00000364133 ENSP00000351905 |
| 9 | ENSP00000304283 ENSP00000269321 ENSP00000348461 ENSP00000270202 ENSP00000352121 |
| 16 | ENSP00000304283 ENSP00000269321 ENSP00000314458 ENSP00000223023 ENSP00000339007 ENSP00000304895 ENSP00000353483 ENSP00000250894 ENSP00000352157 |
| 10 | ENSP00000304283 ENSP00000269321 ENSP00000314458 ENSP00000223023 ENSP00000339007 ENSP00000304895 ENSP00000353483 |
| 9 | ENSP00000304283 ENSP00000302269 ENSP00000264033 ENSP00000275493 ENSP00000354394 |
| 9 | ENSP00000304283 ENSP00000269321 ENSP00000348461 ENSP00000270202 ENSP00000417281 ENSP00000355153 |
| 10 | ENSP00000304283 ENSP00000269321 ENSP00000348461 ENSP00000270202 ENSP00000417281 ENSP00000267163 ENSP00000355249 |
| 13 | ENSP00000304283 ENSP00000302269 ENSP00000264033 ENSP00000344818 ENSP00000364133 ENSP00000351905 ENSP00000355896 |
| 9 | ENSP00000304283 ENSP00000302269 ENSP00000264033 ENSP00000344818 ENSP00000358622 |
| 10 | ENSP00000304283 ENSP00000269321 ENSP00000348461 ENSP00000270202 ENSP00000359424 |
| 10 | ENSP00000304283 ENSP00000269321 ENSP00000314458 ENSP00000223023 ENSP00000339007 ENSP00000384675 ENSP00000309845 ENSP00000361120 |
| 10 | ENSP00000304283 ENSP00000269321 ENSP00000348461 ENSP00000270202 ENSP00000335153 ENSP00000338018 ENSP00000361125 |
| 9 | ENSP00000304283 ENSP00000302269 ENSP00000264033 ENSP00000344818 ENSP00000364133 |
| 21 | ENSP00000304283 ENSP00000269321 ENSP00000348461 ENSP00000270202 ENSP00000335153 ENSP00000251849 ENSP00000302486 ENSP00000366244 |
| 10 | ENSP00000304283 ENSP00000269321 ENSP00000348461 ENSP00000270202 ENSP00000366563 |
| 10 | ENSP00000304283 ENSP00000302269 ENSP00000264033 ENSP00000344818 ENSP00000350283 ENSP00000369497 |
| 16 | ENSP00000304283 ENSP00000302269 ENSP00000264033 ENSP00000274335 ENSP00000303830 ENSP00000348986 ENSP00000375892 |
| 9 | ENSP00000304283 ENSP00000302269 ENSP00000264033 ENSP00000344818 ENSP00000384273 |
| 6 | ENSP00000309103 ENSP00000270202 ENSP00000348461 ENSP00000268182 ENSP00000314458 |
| 4 | ENSP00000309103 ENSP00000329623 ENSP00000269305 ENSP00000321410 |
| 5 | ENSP00000309103 ENSP00000329623 ENSP00000269305 ENSP00000344818 ENSP00000347858 ENSP00000330237 |
| 4 | ENSP00000309103 ENSP00000329623 ENSP00000269305 ENSP00000263253 ENSP00000332973 |
| 4 | ENSP00000309103 ENSP00000329623 ENSP00000269305 ENSP00000344818 ENSP00000339151 |
| 4 | ENSP00000309103 ENSP00000329623 ENSP00000269305 ENSP00000263253 ENSP00000341551 |
| 15 | ENSP00000309103 ENSP00000270202 ENSP00000348461 ENSP00000298316 ENSP00000342793 |
| 5 | ENSP00000309103 ENSP00000329623 ENSP00000269305 ENSP00000263253 ENSP00000264657 ENSP00000343204 |
| 4 | ENSP00000309103 ENSP00000329623 ENSP00000269305 ENSP00000329357 ENSP00000345571 |
| 4 | ENSP00000309103 ENSP00000270202 ENSP00000348461 |
| 5 | ENSP00000309103 ENSP00000329623 ENSP00000269305 ENSP00000344818 ENSP00000364133 ENSP00000351905 |
| 4 | ENSP00000309103 ENSP00000270202 ENSP00000352121 |
| 9 | ENSP00000309103 ENSP00000329623 ENSP00000269305 ENSP00000353483 ENSP00000250894 ENSP00000352157 |
| 3 | ENSP00000309103 ENSP00000329623 ENSP00000269305 ENSP00000353483 |
| 4 | ENSP00000309103 ENSP00000329623 ENSP00000269305 ENSP00000262367 ENSP00000354394 |
| 3 | ENSP00000309103 ENSP00000329623 ENSP00000269305 ENSP00000355153 |
| 5 | ENSP00000309103 ENSP00000309503 ENSP00000251849 ENSP00000267163 ENSP00000355249 |
| 8 | ENSP00000309103 ENSP00000329623 ENSP00000269305 ENSP00000344818 ENSP00000364133 ENSP00000351905 ENSP00000355896 |
| 4 | ENSP00000309103 ENSP00000329623 ENSP00000269305 ENSP00000344818 ENSP00000358622 |
| 5 | ENSP00000309103 ENSP00000270202 ENSP00000359424 |
| 4 | ENSP00000309103 ENSP00000309503 ENSP00000251849 ENSP00000309845 ENSP00000361120 |
| 4 | ENSP00000309103 ENSP00000329623 ENSP00000269305 ENSP00000338018 ENSP00000361125 |
| 4 | ENSP00000309103 ENSP00000329623 ENSP00000269305 ENSP00000344818 ENSP00000364133 |
| 14 | ENSP00000309103 ENSP00000309503 ENSP00000251849 ENSP00000302486 ENSP00000366244 |
| 5 | ENSP00000309103 ENSP00000270202 ENSP00000366563 |
| 4 | ENSP00000309103 ENSP00000329623 ENSP00000269305 ENSP00000267868 ENSP00000369497 |
| 12 | ENSP00000309103 ENSP00000270202 ENSP00000348986 ENSP00000375892 |
| 4 | ENSP00000309103 ENSP00000329623 ENSP00000269305 ENSP00000262367 ENSP00000384273 |
| 6 | ENSP00000314458 ENSP00000278568 ENSP00000288986 ENSP00000264033 ENSP00000344818 ENSP00000360266 ENSP00000321410 |
| 6 | ENSP00000314458 ENSP00000278568 ENSP00000288986 ENSP00000264033 ENSP00000344818 ENSP00000347858 ENSP00000330237 |
| 5 | ENSP00000314458 ENSP00000278568 ENSP00000288986 ENSP00000264033 ENSP00000344818 ENSP00000332973 |
| 5 | ENSP00000314458 ENSP00000278568 ENSP00000288986 ENSP00000264033 ENSP00000344818 ENSP00000339151 |
| 5 | ENSP00000314458 ENSP00000278568 ENSP00000288986 ENSP00000264033 ENSP00000344818 ENSP00000341551 |
| 13 | ENSP00000314458 ENSP00000268182 ENSP00000348461 ENSP00000298316 ENSP00000342793 |
| 4 | ENSP00000314458 ENSP00000223023 ENSP00000339007 ENSP00000304895 ENSP00000343204 |
| 5 | ENSP00000314458 ENSP00000278568 ENSP00000288986 ENSP00000264033 ENSP00000344818 ENSP00000345571 |
| 2 | ENSP00000314458 ENSP00000268182 ENSP00000348461 |
| 6 | ENSP00000314458 ENSP00000278568 ENSP00000288986 ENSP00000264033 ENSP00000344818 ENSP00000364133 ENSP00000351905 |
| 6 | ENSP00000314458 ENSP00000268182 ENSP00000348461 ENSP00000270202 ENSP00000352121 |
| 11 | ENSP00000314458 ENSP00000223023 ENSP00000339007 ENSP00000304895 ENSP00000353483 ENSP00000250894 ENSP00000352157 |
| 5 | ENSP00000314458 ENSP00000223023 ENSP00000339007 ENSP00000304895 ENSP00000353483 |
| 4 | ENSP00000314458 ENSP00000223023 ENSP00000339007 ENSP00000275493 ENSP00000354394 |
| 6 | ENSP00000314458 ENSP00000223023 ENSP00000339007 ENSP00000361423 ENSP00000278616 ENSP00000269305 ENSP00000355153 |
| 6 | ENSP00000314458 ENSP00000223023 ENSP00000339007 ENSP00000361423 ENSP00000267163 ENSP00000355249 |
| 9 | ENSP00000314458 ENSP00000278568 ENSP00000288986 ENSP00000264033 ENSP00000344818 ENSP00000364133 ENSP00000351905 ENSP00000355896 |
| 5 | ENSP00000314458 ENSP00000278568 ENSP00000288986 ENSP00000264033 ENSP00000344818 ENSP00000358622 |
| 6 | ENSP00000314458 ENSP00000278568 ENSP00000288986 ENSP00000264033 ENSP00000344818 ENSP00000216797 ENSP00000359424 |
| 5 | ENSP00000314458 ENSP00000223023 ENSP00000339007 ENSP00000384675 ENSP00000309845 ENSP00000361120 |
| 6 | ENSP00000314458 ENSP00000223023 ENSP00000339007 ENSP00000269571 ENSP00000335153 ENSP00000338018 ENSP00000361125 |
| 5 | ENSP00000314458 ENSP00000278568 ENSP00000288986 ENSP00000264033 ENSP00000344818 ENSP00000364133 |
| 17 | ENSP00000314458 ENSP00000223023 ENSP00000339007 ENSP00000384675 ENSP00000309845 ENSP00000251849 ENSP00000302486 ENSP00000366244 |
| 7 | ENSP00000314458 ENSP00000268182 ENSP00000348461 ENSP00000270202 ENSP00000366563 |
| 6 | ENSP00000314458 ENSP00000223023 ENSP00000339007 ENSP00000361423 ENSP00000278616 ENSP00000287647 ENSP00000369497 |
| 11 | ENSP00000314458 ENSP00000223023 ENSP00000339007 ENSP00000274335 ENSP00000303830 ENSP00000348986 ENSP00000375892 |
| 5 | ENSP00000314458 ENSP00000278568 ENSP00000288986 ENSP00000264033 ENSP00000344818 ENSP00000384273 |
| 4 | ENSP00000321410 ENSP00000360266 ENSP00000344818 ENSP00000347858 ENSP00000330237 |
| 3 | ENSP00000321410 ENSP00000360266 ENSP00000332973 |
| 3 | ENSP00000321410 ENSP00000360266 ENSP00000344818 ENSP00000339151 |
| 3 | ENSP00000321410 ENSP00000360266 ENSP00000263253 ENSP00000341551 |
| 14 | ENSP00000321410 ENSP00000360266 ENSP00000344818 ENSP00000003084 ENSP00000262613 ENSP00000338934 ENSP00000284384 ENSP00000342793 |
| 4 | ENSP00000321410 ENSP00000360266 ENSP00000263253 ENSP00000264657 ENSP00000343204 |
| 3 | ENSP00000321410 ENSP00000360266 ENSP00000344818 ENSP00000345571 |
| 5 | ENSP00000321410 ENSP00000360266 ENSP00000344818 ENSP00000270202 ENSP00000348461 |
| 4 | ENSP00000321410 ENSP00000360266 ENSP00000344818 ENSP00000364133 ENSP00000351905 |
| 5 | ENSP00000321410 ENSP00000360266 ENSP00000344818 ENSP00000270202 ENSP00000352121 |
| 8 | ENSP00000321410 ENSP00000360266 ENSP00000353483 ENSP00000250894 ENSP00000352157 |
| 2 | ENSP00000321410 ENSP00000360266 ENSP00000353483 |
| 3 | ENSP00000321410 ENSP00000360266 ENSP00000263253 ENSP00000354394 |
| 3 | ENSP00000321410 ENSP00000269305 ENSP00000355153 |
| 5 | ENSP00000321410 ENSP00000360266 ENSP00000344818 ENSP00000227507 ENSP00000267163 ENSP00000355249 |
| 7 | ENSP00000321410 ENSP00000360266 ENSP00000344818 ENSP00000364133 ENSP00000351905 ENSP00000355896 |
| 3 | ENSP00000321410 ENSP00000360266 ENSP00000344818 ENSP00000358622 |
| 4 | ENSP00000321410 ENSP00000360266 ENSP00000344818 ENSP00000216797 ENSP00000359424 |
| 6 | ENSP00000321410 ENSP00000360266 ENSP00000215832 ENSP00000302486 ENSP00000251849 ENSP00000309845 ENSP00000361120 |
| 4 | ENSP00000321410 ENSP00000360266 ENSP00000263253 ENSP00000338018 ENSP00000361125 |
| 3 | ENSP00000321410 ENSP00000360266 ENSP00000344818 ENSP00000364133 |
| 14 | ENSP00000321410 ENSP00000360266 ENSP00000215832 ENSP00000302486 ENSP00000366244 |
| 6 | ENSP00000321410 ENSP00000360266 ENSP00000344818 ENSP00000270202 ENSP00000366563 |
| 4 | ENSP00000321410 ENSP00000269305 ENSP00000267868 ENSP00000369497 |
| 12 | ENSP00000321410 ENSP00000360266 ENSP00000344818 ENSP00000264033 ENSP00000274335 ENSP00000303830 ENSP00000348986 ENSP00000375892 |
| 3 | ENSP00000321410 ENSP00000360266 ENSP00000263253 ENSP00000384273 |
| 3 | ENSP00000330237 ENSP00000347858 ENSP00000344818 ENSP00000332973 |
| 3 | ENSP00000330237 ENSP00000347858 ENSP00000344818 ENSP00000339151 |
| 3 | ENSP00000330237 ENSP00000347858 ENSP00000344818 ENSP00000341551 |
| 14 | ENSP00000330237 ENSP00000347858 ENSP00000344818 ENSP00000003084 ENSP00000262613 ENSP00000338934 ENSP00000284384 ENSP00000342793 |
| 5 | ENSP00000330237 ENSP00000347858 ENSP00000344818 ENSP00000227507 ENSP00000264657 ENSP00000343204 |
| 3 | ENSP00000330237 ENSP00000347858 ENSP00000344818 ENSP00000345571 |
| 5 | ENSP00000330237 ENSP00000347858 ENSP00000344818 ENSP00000270202 ENSP00000348461 |
| 4 | ENSP00000330237 ENSP00000347858 ENSP00000344818 ENSP00000364133 ENSP00000351905 |
| 5 | ENSP00000330237 ENSP00000347858 ENSP00000344818 ENSP00000270202 ENSP00000352121 |
| 10 | ENSP00000330237 ENSP00000347858 ENSP00000344818 ENSP00000269305 ENSP00000353483 ENSP00000250894 ENSP00000352157 |
| 4 | ENSP00000330237 ENSP00000347858 ENSP00000344818 ENSP00000269305 ENSP00000353483 |
| 4 | ENSP00000330237 ENSP00000347858 ENSP00000344818 ENSP00000275493 ENSP00000354394 |
| 4 | ENSP00000330237 ENSP00000347858 ENSP00000344818 ENSP00000269305 ENSP00000355153 |
| 5 | ENSP00000330237 ENSP00000347858 ENSP00000344818 ENSP00000227507 ENSP00000267163 ENSP00000355249 |
| 7 | ENSP00000330237 ENSP00000347858 ENSP00000344818 ENSP00000364133 ENSP00000351905 ENSP00000355896 |
| 3 | ENSP00000330237 ENSP00000347858 ENSP00000344818 ENSP00000358622 |
| 4 | ENSP00000330237 ENSP00000347858 ENSP00000344818 ENSP00000216797 ENSP00000359424 |
| 7 | ENSP00000330237 ENSP00000347858 ENSP00000344818 ENSP00000206249 ENSP00000335153 ENSP00000251849 ENSP00000309845 ENSP00000361120 |
| 4 | ENSP00000330237 ENSP00000347858 ENSP00000344818 ENSP00000338018 ENSP00000361125 |
| 3 | ENSP00000330237 ENSP00000347858 ENSP00000344818 ENSP00000364133 |
| 16 | ENSP00000330237 ENSP00000347858 ENSP00000344818 ENSP00000360266 ENSP00000215832 ENSP00000302486 ENSP00000366244 |
| 6 | ENSP00000330237 ENSP00000347858 ENSP00000344818 ENSP00000270202 ENSP00000366563 |
| 4 | ENSP00000330237 ENSP00000347858 ENSP00000344818 ENSP00000350283 ENSP00000369497 |
| 12 | ENSP00000330237 ENSP00000347858 ENSP00000344818 ENSP00000264033 ENSP00000274335 ENSP00000303830 ENSP00000348986 ENSP00000375892 |
| 3 | ENSP00000330237 ENSP00000347858 ENSP00000344818 ENSP00000384273 |
| 2 | ENSP00000332973 ENSP00000344818 ENSP00000339151 |
| 1 | ENSP00000332973 ENSP00000341551 |
| 13 | ENSP00000332973 ENSP00000344818 ENSP00000003084 ENSP00000262613 ENSP00000338934 ENSP00000284384 ENSP00000342793 |
| 3 | ENSP00000332973 ENSP00000263253 ENSP00000264657 ENSP00000343204 |
| 2 | ENSP00000332973 ENSP00000344818 ENSP00000345571 |
| 4 | ENSP00000332973 ENSP00000344818 ENSP00000270202 ENSP00000348461 |
| 3 | ENSP00000332973 ENSP00000344818 ENSP00000364133 ENSP00000351905 |
| 4 | ENSP00000332973 ENSP00000344818 ENSP00000270202 ENSP00000352121 |
| 9 | ENSP00000332973 ENSP00000263253 ENSP00000269305 ENSP00000353483 ENSP00000250894 ENSP00000352157 |
| 3 | ENSP00000332973 ENSP00000263253 ENSP00000269305 ENSP00000353483 |
| 2 | ENSP00000332973 ENSP00000263253 ENSP00000354394 |
| 3 | ENSP00000332973 ENSP00000263253 ENSP00000269305 ENSP00000355153 |
| 4 | ENSP00000332973 ENSP00000344818 ENSP00000227507 ENSP00000267163 ENSP00000355249 |
| 6 | ENSP00000332973 ENSP00000344818 ENSP00000364133 ENSP00000351905 ENSP00000355896 |
| 2 | ENSP00000332973 ENSP00000344818 ENSP00000358622 |
| 3 | ENSP00000332973 ENSP00000344818 ENSP00000216797 ENSP00000359424 |
| 6 | ENSP00000332973 ENSP00000263253 ENSP00000206249 ENSP00000335153 ENSP00000251849 ENSP00000309845 ENSP00000361120 |
| 3 | ENSP00000332973 ENSP00000263253 ENSP00000338018 ENSP00000361125 |
| 2 | ENSP00000332973 ENSP00000344818 ENSP00000364133 |
| 15 | ENSP00000332973 ENSP00000360266 ENSP00000215832 ENSP00000302486 ENSP00000366244 |
| 5 | ENSP00000332973 ENSP00000344818 ENSP00000270202 ENSP00000366563 |
| 3 | ENSP00000332973 ENSP00000344818 ENSP00000350283 ENSP00000369497 |
| 11 | ENSP00000332973 ENSP00000344818 ENSP00000264033 ENSP00000274335 ENSP00000303830 ENSP00000348986 ENSP00000375892 |
| 2 | ENSP00000332973 ENSP00000263253 ENSP00000384273 |
| 2 | ENSP00000339151 ENSP00000344818 ENSP00000341551 |
| 13 | ENSP00000339151 ENSP00000344818 ENSP00000003084 ENSP00000262613 ENSP00000338934 ENSP00000284384 ENSP00000342793 |
| 4 | ENSP00000339151 ENSP00000344818 ENSP00000227507 ENSP00000264657 ENSP00000343204 |
| 2 | ENSP00000339151 ENSP00000344818 ENSP00000345571 |
| 4 | ENSP00000339151 ENSP00000344818 ENSP00000270202 ENSP00000348461 |
| 3 | ENSP00000339151 ENSP00000344818 ENSP00000364133 ENSP00000351905 |
| 4 | ENSP00000339151 ENSP00000344818 ENSP00000270202 ENSP00000352121 |
| 9 | ENSP00000339151 ENSP00000344818 ENSP00000269305 ENSP00000353483 ENSP00000250894 ENSP00000352157 |
| 3 | ENSP00000339151 ENSP00000344818 ENSP00000269305 ENSP00000353483 |
| 3 | ENSP00000339151 ENSP00000384273 ENSP00000262367 ENSP00000354394 |
| 3 | ENSP00000339151 ENSP00000344818 ENSP00000269305 ENSP00000355153 |
| 4 | ENSP00000339151 ENSP00000344818 ENSP00000227507 ENSP00000267163 ENSP00000355249 |
| 6 | ENSP00000339151 ENSP00000344818 ENSP00000364133 ENSP00000351905 ENSP00000355896 |
| 1 | ENSP00000339151 ENSP00000358622 |
| 1 | ENSP00000339151 ENSP00000359424 |
| 6 | ENSP00000339151 ENSP00000344818 ENSP00000206249 ENSP00000335153 ENSP00000251849 ENSP00000309845 ENSP00000361120 |
| 3 | ENSP00000339151 ENSP00000344818 ENSP00000338018 ENSP00000361125 |
| 2 | ENSP00000339151 ENSP00000344818 ENSP00000364133 |
| 15 | ENSP00000339151 ENSP00000344818 ENSP00000360266 ENSP00000215832 ENSP00000302486 ENSP00000366244 |
| 5 | ENSP00000339151 ENSP00000344818 ENSP00000270202 ENSP00000366563 |
| 3 | ENSP00000339151 ENSP00000344818 ENSP00000350283 ENSP00000369497 |
| 11 | ENSP00000339151 ENSP00000344818 ENSP00000264033 ENSP00000274335 ENSP00000303830 ENSP00000348986 ENSP00000375892 |
| 1 | ENSP00000339151 ENSP00000384273 |
| 13 | ENSP00000341551 ENSP00000344818 ENSP00000003084 ENSP00000262613 ENSP00000338934 ENSP00000284384 ENSP00000342793 |
| 3 | ENSP00000341551 ENSP00000263253 ENSP00000264657 ENSP00000343204 |
| 2 | ENSP00000341551 ENSP00000329357 ENSP00000345571 |
| 4 | ENSP00000341551 ENSP00000344818 ENSP00000270202 ENSP00000348461 |
| 3 | ENSP00000341551 ENSP00000262160 ENSP00000364133 ENSP00000351905 |
| 4 | ENSP00000341551 ENSP00000344818 ENSP00000270202 ENSP00000352121 |
| 9 | ENSP00000341551 ENSP00000263253 ENSP00000269305 ENSP00000353483 ENSP00000250894 ENSP00000352157 |
| 3 | ENSP00000341551 ENSP00000263253 ENSP00000269305 ENSP00000353483 |
| 2 | ENSP00000341551 ENSP00000263253 ENSP00000354394 |
| 3 | ENSP00000341551 ENSP00000263253 ENSP00000269305 ENSP00000355153 |
| 4 | ENSP00000341551 ENSP00000344818 ENSP00000227507 ENSP00000267163 ENSP00000355249 |
| 6 | ENSP00000341551 ENSP00000262160 ENSP00000364133 ENSP00000351905 ENSP00000355896 |
| 2 | ENSP00000341551 ENSP00000344818 ENSP00000358622 |
| 3 | ENSP00000341551 ENSP00000344818 ENSP00000216797 ENSP00000359424 |
| 6 | ENSP00000341551 ENSP00000263253 ENSP00000206249 ENSP00000335153 ENSP00000251849 ENSP00000309845 ENSP00000361120 |
| 3 | ENSP00000341551 ENSP00000263253 ENSP00000338018 ENSP00000361125 |
| 2 | ENSP00000341551 ENSP00000262160 ENSP00000364133 |
| 15 | ENSP00000341551 ENSP00000263253 ENSP00000360266 ENSP00000215832 ENSP00000302486 ENSP00000366244 |
| 5 | ENSP00000341551 ENSP00000344818 ENSP00000270202 ENSP00000366563 |
| 3 | ENSP00000341551 ENSP00000344818 ENSP00000350283 ENSP00000369497 |
| 11 | ENSP00000341551 ENSP00000344818 ENSP00000264033 ENSP00000274335 ENSP00000303830 ENSP00000348986 ENSP00000375892 |
| 2 | ENSP00000341551 ENSP00000263253 ENSP00000384273 |
| 14 | ENSP00000342793 ENSP00000284384 ENSP00000282561 ENSP00000350941 ENSP00000264657 ENSP00000343204 |
| 13 | ENSP00000342793 ENSP00000284384 ENSP00000338934 ENSP00000262613 ENSP00000003084 ENSP00000344818 ENSP00000345571 |
| 11 | ENSP00000342793 ENSP00000298316 ENSP00000348461 |
| 14 | ENSP00000342793 ENSP00000284384 ENSP00000338934 ENSP00000262613 ENSP00000003084 ENSP00000344818 ENSP00000364133 ENSP00000351905 |
| 15 | ENSP00000342793 ENSP00000298316 ENSP00000348461 ENSP00000270202 ENSP00000352121 |
| 20 | ENSP00000342793 ENSP00000284384 ENSP00000254066 ENSP00000268058 ENSP00000269305 ENSP00000353483 ENSP00000250894 ENSP00000352157 |
| 14 | ENSP00000342793 ENSP00000284384 ENSP00000254066 ENSP00000268058 ENSP00000269305 ENSP00000353483 |
| 14 | ENSP00000342793 ENSP00000284384 ENSP00000254066 ENSP00000320940 ENSP00000262367 ENSP00000354394 |
| 14 | ENSP00000342793 ENSP00000284384 ENSP00000254066 ENSP00000268058 ENSP00000269305 ENSP00000355153 |
| 15 | ENSP00000342793 ENSP00000284384 ENSP00000338934 ENSP00000262613 ENSP00000003084 ENSP00000344818 ENSP00000227507 ENSP00000267163 ENSP00000355249 |
| 17 | ENSP00000342793 ENSP00000284384 ENSP00000338934 ENSP00000262613 ENSP00000003084 ENSP00000344818 ENSP00000364133 ENSP00000351905 ENSP00000355896 |
| 13 | ENSP00000342793 ENSP00000284384 ENSP00000338934 ENSP00000262613 ENSP00000003084 ENSP00000344818 ENSP00000358622 |
| 14 | ENSP00000342793 ENSP00000284384 ENSP00000338934 ENSP00000262613 ENSP00000003084 ENSP00000344818 ENSP00000216797 ENSP00000359424 |
| 16 | ENSP00000342793 ENSP00000284384 ENSP00000282561 ENSP00000350941 ENSP00000309845 ENSP00000361120 |
| 14 | ENSP00000342793 ENSP00000284384 ENSP00000338934 ENSP00000262613 ENSP00000003084 ENSP00000344818 ENSP00000338018 ENSP00000361125 |
| 13 | ENSP00000342793 ENSP00000284384 ENSP00000338934 ENSP00000262613 ENSP00000003084 ENSP00000344818 ENSP00000364133 |
| 26 | ENSP00000342793 ENSP00000284384 ENSP00000338934 ENSP00000262613 ENSP00000003084 ENSP00000344818 ENSP00000360266 ENSP00000215832 ENSP00000302486 ENSP00000366244 |
| 16 | ENSP00000342793 ENSP00000298316 ENSP00000348461 ENSP00000270202 ENSP00000366563 |
| 14 | ENSP00000342793 ENSP00000284384 ENSP00000338934 ENSP00000262613 ENSP00000003084 ENSP00000344818 ENSP00000350283 ENSP00000369497 |
| 21 | ENSP00000342793 ENSP00000284384 ENSP00000282561 ENSP00000350941 ENSP00000360683 ENSP00000303830 ENSP00000348986 ENSP00000375892 |
| 13 | ENSP00000342793 ENSP00000284384 ENSP00000338934 ENSP00000262613 ENSP00000003084 ENSP00000344818 ENSP00000384273 |
| 4 | ENSP00000343204 ENSP00000264657 ENSP00000227507 ENSP00000267163 ENSP00000345571 |
| 5 | ENSP00000343204 ENSP00000264657 ENSP00000348461 |
| 5 | ENSP00000343204 ENSP00000264657 ENSP00000227507 ENSP00000344818 ENSP00000364133 ENSP00000351905 |
| 6 | ENSP00000343204 ENSP00000304895 ENSP00000263967 ENSP00000270202 ENSP00000352121 |
| 9 | ENSP00000343204 ENSP00000304895 ENSP00000353483 ENSP00000250894 ENSP00000352157 |
| 3 | ENSP00000343204 ENSP00000304895 ENSP00000353483 |
| 2 | ENSP00000343204 ENSP00000354394 |
| 4 | ENSP00000343204 ENSP00000264657 ENSP00000227507 ENSP00000257904 ENSP00000355153 |
| 4 | ENSP00000343204 ENSP00000264657 ENSP00000227507 ENSP00000267163 ENSP00000355249 |
| 8 | ENSP00000343204 ENSP00000264657 ENSP00000227507 ENSP00000344818 ENSP00000364133 ENSP00000351905 ENSP00000355896 |
| 4 | ENSP00000343204 ENSP00000264657 ENSP00000227507 ENSP00000344818 ENSP00000358622 |
| 4 | ENSP00000343204 ENSP00000264657 ENSP00000263253 ENSP00000384273 ENSP00000359424 |
| 4 | ENSP00000343204 ENSP00000304895 ENSP00000263967 ENSP00000309845 ENSP00000361120 |
| 4 | ENSP00000343204 ENSP00000264657 ENSP00000263253 ENSP00000338018 ENSP00000361125 |
| 4 | ENSP00000343204 ENSP00000264657 ENSP00000227507 ENSP00000344818 ENSP00000364133 |
| 16 | ENSP00000343204 ENSP00000264657 ENSP00000263253 ENSP00000360266 ENSP00000215832 ENSP00000302486 ENSP00000366244 |
| 7 | ENSP00000343204 ENSP00000304895 ENSP00000263967 ENSP00000270202 ENSP00000366563 |
| 5 | ENSP00000343204 ENSP00000264657 ENSP00000263253 ENSP00000269305 ENSP00000267868 ENSP00000369497 |
| 9 | ENSP00000343204 ENSP00000304895 ENSP00000303830 ENSP00000348986 ENSP00000375892 |
| 3 | ENSP00000343204 ENSP00000264657 ENSP00000263253 ENSP00000384273 |
| 4 | ENSP00000345571 ENSP00000344818 ENSP00000270202 ENSP00000348461 |
| 3 | ENSP00000345571 ENSP00000344818 ENSP00000364133 ENSP00000351905 |
| 4 | ENSP00000345571 ENSP00000344818 ENSP00000270202 ENSP00000352121 |
| 9 | ENSP00000345571 ENSP00000329357 ENSP00000269305 ENSP00000353483 ENSP00000250894 ENSP00000352157 |
| 3 | ENSP00000345571 ENSP00000329357 ENSP00000269305 ENSP00000353483 |
| 3 | ENSP00000345571 ENSP00000329357 ENSP00000263253 ENSP00000354394 |
| 3 | ENSP00000345571 ENSP00000267163 ENSP00000257904 ENSP00000355153 |
| 2 | ENSP00000345571 ENSP00000267163 ENSP00000355249 |
| 6 | ENSP00000345571 ENSP00000344818 ENSP00000364133 ENSP00000351905 ENSP00000355896 |
| 2 | ENSP00000345571 ENSP00000344818 ENSP00000358622 |
| 3 | ENSP00000345571 ENSP00000344818 ENSP00000216797 ENSP00000359424 |
| 5 | ENSP00000345571 ENSP00000267163 ENSP00000251849 ENSP00000309845 ENSP00000361120 |
| 3 | ENSP00000345571 ENSP00000344818 ENSP00000338018 ENSP00000361125 |
| 2 | ENSP00000345571 ENSP00000344818 ENSP00000364133 |
| 15 | ENSP00000345571 ENSP00000344818 ENSP00000360266 ENSP00000215832 ENSP00000302486 ENSP00000366244 |
| 5 | ENSP00000345571 ENSP00000344818 ENSP00000270202 ENSP00000366563 |
| 3 | ENSP00000345571 ENSP00000344818 ENSP00000350283 ENSP00000369497 |
| 11 | ENSP00000345571 ENSP00000344818 ENSP00000264033 ENSP00000274335 ENSP00000303830 ENSP00000348986 ENSP00000375892 |
| 2 | ENSP00000345571 ENSP00000344818 ENSP00000384273 |
| 5 | ENSP00000348461 ENSP00000270202 ENSP00000344818 ENSP00000364133 ENSP00000351905 |
| 4 | ENSP00000348461 ENSP00000270202 ENSP00000352121 |
| 11 | ENSP00000348461 ENSP00000270202 ENSP00000335153 ENSP00000269305 ENSP00000353483 ENSP00000250894 ENSP00000352157 |
| 5 | ENSP00000348461 ENSP00000270202 ENSP00000335153 ENSP00000269305 ENSP00000353483 |
| 5 | ENSP00000348461 ENSP00000278568 ENSP00000288986 ENSP00000264033 ENSP00000275493 ENSP00000354394 |
| 4 | ENSP00000348461 ENSP00000270202 ENSP00000417281 ENSP00000355153 |
| 5 | ENSP00000348461 ENSP00000270202 ENSP00000417281 ENSP00000267163 ENSP00000355249 |
| 8 | ENSP00000348461 ENSP00000270202 ENSP00000344818 ENSP00000364133 ENSP00000351905 ENSP00000355896 |
| 4 | ENSP00000348461 ENSP00000270202 ENSP00000344818 ENSP00000358622 |
| 5 | ENSP00000348461 ENSP00000270202 ENSP00000359424 |
| 6 | ENSP00000348461 ENSP00000270202 ENSP00000335153 ENSP00000251849 ENSP00000309845 ENSP00000361120 |
| 5 | ENSP00000348461 ENSP00000270202 ENSP00000335153 ENSP00000338018 ENSP00000361125 |
| 4 | ENSP00000348461 ENSP00000270202 ENSP00000344818 ENSP00000364133 |
| 16 | ENSP00000348461 ENSP00000270202 ENSP00000335153 ENSP00000251849 ENSP00000302486 ENSP00000366244 |
| 5 | ENSP00000348461 ENSP00000270202 ENSP00000366563 |
| 5 | ENSP00000348461 ENSP00000270202 ENSP00000344818 ENSP00000350283 ENSP00000369497 |
| 12 | ENSP00000348461 ENSP00000270202 ENSP00000348986 ENSP00000375892 |
| 4 | ENSP00000348461 ENSP00000270202 ENSP00000344818 ENSP00000384273 |
| 5 | ENSP00000351905 ENSP00000364133 ENSP00000344818 ENSP00000270202 ENSP00000352121 |
| 10 | ENSP00000351905 ENSP00000364133 ENSP00000344818 ENSP00000269305 ENSP00000353483 ENSP00000250894 ENSP00000352157 |
| 4 | ENSP00000351905 ENSP00000364133 ENSP00000344818 ENSP00000269305 ENSP00000353483 |
| 4 | ENSP00000351905 ENSP00000364133 ENSP00000262160 ENSP00000262367 ENSP00000354394 |
| 4 | ENSP00000351905 ENSP00000364133 ENSP00000344818 ENSP00000269305 ENSP00000355153 |
| 5 | ENSP00000351905 ENSP00000364133 ENSP00000344818 ENSP00000227507 ENSP00000267163 ENSP00000355249 |
| 3 | ENSP00000351905 ENSP00000355896 |
| 3 | ENSP00000351905 ENSP00000364133 ENSP00000344818 ENSP00000358622 |
| 4 | ENSP00000351905 ENSP00000364133 ENSP00000344818 ENSP00000216797 ENSP00000359424 |
| 7 | ENSP00000351905 ENSP00000364133 ENSP00000344818 ENSP00000206249 ENSP00000335153 ENSP00000251849 ENSP00000309845 ENSP00000361120 |
| 4 | ENSP00000351905 ENSP00000364133 ENSP00000344818 ENSP00000338018 ENSP00000361125 |
| 1 | ENSP00000351905 ENSP00000364133 |
| 16 | ENSP00000351905 ENSP00000364133 ENSP00000344818 ENSP00000360266 ENSP00000215832 ENSP00000302486 ENSP00000366244 |
| 6 | ENSP00000351905 ENSP00000364133 ENSP00000344818 ENSP00000270202 ENSP00000366563 |
| 4 | ENSP00000351905 ENSP00000364133 ENSP00000344818 ENSP00000350283 ENSP00000369497 |
| 12 | ENSP00000351905 ENSP00000364133 ENSP00000344818 ENSP00000264033 ENSP00000274335 ENSP00000303830 ENSP00000348986 ENSP00000375892 |
| 3 | ENSP00000351905 ENSP00000364133 ENSP00000344818 ENSP00000384273 |
| 11 | ENSP00000352121 ENSP00000270202 ENSP00000335153 ENSP00000269305 ENSP00000353483 ENSP00000250894 ENSP00000352157 |
| 5 | ENSP00000352121 ENSP00000270202 ENSP00000335153 ENSP00000269305 ENSP00000353483 |
| 5 | ENSP00000352121 ENSP00000270202 ENSP00000344818 ENSP00000275493 ENSP00000354394 |
| 4 | ENSP00000352121 ENSP00000270202 ENSP00000417281 ENSP00000355153 |
| 5 | ENSP00000352121 ENSP00000270202 ENSP00000417281 ENSP00000267163 ENSP00000355249 |
| 8 | ENSP00000352121 ENSP00000270202 ENSP00000344818 ENSP00000364133 ENSP00000351905 ENSP00000355896 |
| 4 | ENSP00000352121 ENSP00000270202 ENSP00000344818 ENSP00000358622 |
| 5 | ENSP00000352121 ENSP00000270202 ENSP00000359424 |
| 6 | ENSP00000352121 ENSP00000309845 ENSP00000361120 |
| 5 | ENSP00000352121 ENSP00000270202 ENSP00000335153 ENSP00000338018 ENSP00000361125 |
| 4 | ENSP00000352121 ENSP00000270202 ENSP00000344818 ENSP00000364133 |
| 16 | ENSP00000352121 ENSP00000270202 ENSP00000335153 ENSP00000251849 ENSP00000302486 ENSP00000366244 |
| 5 | ENSP00000352121 ENSP00000270202 ENSP00000366563 |
| 5 | ENSP00000352121 ENSP00000270202 ENSP00000344818 ENSP00000350283 ENSP00000369497 |
| 12 | ENSP00000352121 ENSP00000270202 ENSP00000348986 ENSP00000375892 |
| 4 | ENSP00000352121 ENSP00000270202 ENSP00000344818 ENSP00000384273 |
| 6 | ENSP00000352157 ENSP00000250894 ENSP00000353483 |
| 9 | ENSP00000352157 ENSP00000250894 ENSP00000353483 ENSP00000269305 ENSP00000262367 ENSP00000354394 |
| 8 | ENSP00000352157 ENSP00000250894 ENSP00000353483 ENSP00000269305 ENSP00000355153 |
| 10 | ENSP00000352157 ENSP00000250894 ENSP00000353483 ENSP00000269305 ENSP00000266970 ENSP00000267163 ENSP00000355249 |
| 13 | ENSP00000352157 ENSP00000250894 ENSP00000353483 ENSP00000269305 ENSP00000344818 ENSP00000364133 ENSP00000351905 ENSP00000355896 |
| 9 | ENSP00000352157 ENSP00000250894 ENSP00000353483 ENSP00000269305 ENSP00000344818 ENSP00000358622 |
| 10 | ENSP00000352157 ENSP00000250894 ENSP00000353483 ENSP00000269305 ENSP00000344818 ENSP00000216797 ENSP00000359424 |
| 11 | ENSP00000352157 ENSP00000250894 ENSP00000353483 ENSP00000269305 ENSP00000335153 ENSP00000251849 ENSP00000309845 ENSP00000361120 |
| 9 | ENSP00000352157 ENSP00000250894 ENSP00000353483 ENSP00000269305 ENSP00000338018 ENSP00000361125 |
| 9 | ENSP00000352157 ENSP00000250894 ENSP00000353483 ENSP00000269305 ENSP00000344818 ENSP00000364133 |
| 20 | ENSP00000352157 ENSP00000250894 ENSP00000353483 ENSP00000360266 ENSP00000215832 ENSP00000302486 ENSP00000366244 |
| 12 | ENSP00000352157 ENSP00000250894 ENSP00000353483 ENSP00000269305 ENSP00000335153 ENSP00000270202 ENSP00000366563 |
| 9 | ENSP00000352157 ENSP00000250894 ENSP00000353483 ENSP00000269305 ENSP00000267868 ENSP00000369497 |
| 16 | ENSP00000352157 ENSP00000250894 ENSP00000353483 ENSP00000304895 ENSP00000303830 ENSP00000348986 ENSP00000375892 |
| 9 | ENSP00000352157 ENSP00000250894 ENSP00000353483 ENSP00000269305 ENSP00000262367 ENSP00000384273 |
| 3 | ENSP00000353483 ENSP00000269305 ENSP00000262367 ENSP00000354394 |
| 2 | ENSP00000353483 ENSP00000269305 ENSP00000355153 |
| 4 | ENSP00000353483 ENSP00000269305 ENSP00000266970 ENSP00000267163 ENSP00000355249 |
| 7 | ENSP00000353483 ENSP00000269305 ENSP00000344818 ENSP00000364133 ENSP00000351905 ENSP00000355896 |
| 3 | ENSP00000353483 ENSP00000269305 ENSP00000344818 ENSP00000358622 |
| 4 | ENSP00000353483 ENSP00000269305 ENSP00000344818 ENSP00000216797 ENSP00000359424 |
| 5 | ENSP00000353483 ENSP00000269305 ENSP00000335153 ENSP00000251849 ENSP00000309845 ENSP00000361120 |
| 3 | ENSP00000353483 ENSP00000269305 ENSP00000338018 ENSP00000361125 |
| 3 | ENSP00000353483 ENSP00000269305 ENSP00000344818 ENSP00000364133 |
| 14 | ENSP00000353483 ENSP00000360266 ENSP00000215832 ENSP00000302486 ENSP00000366244 |
| 6 | ENSP00000353483 ENSP00000269305 ENSP00000335153 ENSP00000270202 ENSP00000366563 |
| 3 | ENSP00000353483 ENSP00000269305 ENSP00000267868 ENSP00000369497 |
| 10 | ENSP00000353483 ENSP00000304895 ENSP00000303830 ENSP00000348986 ENSP00000375892 |
| 3 | ENSP00000353483 ENSP00000269305 ENSP00000262367 ENSP00000384273 |
| 3 | ENSP00000354394 ENSP00000262367 ENSP00000269305 ENSP00000355153 |
| 5 | ENSP00000354394 ENSP00000262367 ENSP00000206249 ENSP00000227507 ENSP00000267163 ENSP00000355249 |
| 7 | ENSP00000354394 ENSP00000262367 ENSP00000262160 ENSP00000364133 ENSP00000351905 ENSP00000355896 |
| 3 | ENSP00000354394 ENSP00000275493 ENSP00000344818 ENSP00000358622 |
| 3 | ENSP00000354394 ENSP00000262367 ENSP00000384273 ENSP00000359424 |
| 5 | ENSP00000354394 ENSP00000371067 ENSP00000304895 ENSP00000263967 ENSP00000309845 ENSP00000361120 |
| 3 | ENSP00000354394 ENSP00000262367 ENSP00000338018 ENSP00000361125 |
| 3 | ENSP00000354394 ENSP00000262367 ENSP00000262160 ENSP00000364133 |
| 15 | ENSP00000354394 ENSP00000263253 ENSP00000360266 ENSP00000215832 ENSP00000302486 ENSP00000366244 |
| 6 | ENSP00000354394 ENSP00000275493 ENSP00000344818 ENSP00000270202 ENSP00000366563 |
| 4 | ENSP00000354394 ENSP00000262367 ENSP00000269305 ENSP00000267868 ENSP00000369497 |
| 10 | ENSP00000354394 ENSP00000371067 ENSP00000304895 ENSP00000303830 ENSP00000348986 ENSP00000375892 |
| 2 | ENSP00000354394 ENSP00000262367 ENSP00000384273 |
| 3 | ENSP00000355153 ENSP00000257904 ENSP00000267163 ENSP00000355249 |
| 7 | ENSP00000355153 ENSP00000269305 ENSP00000344818 ENSP00000364133 ENSP00000351905 ENSP00000355896 |
| 3 | ENSP00000355153 ENSP00000269305 ENSP00000344818 ENSP00000358622 |
| 4 | ENSP00000355153 ENSP00000269305 ENSP00000344818 ENSP00000216797 ENSP00000359424 |
| 5 | ENSP00000355153 ENSP00000269305 ENSP00000335153 ENSP00000251849 ENSP00000309845 ENSP00000361120 |
| 3 | ENSP00000355153 ENSP00000269305 ENSP00000338018 ENSP00000361125 |
| 3 | ENSP00000355153 ENSP00000269305 ENSP00000344818 ENSP00000364133 |
| 15 | ENSP00000355153 ENSP00000269305 ENSP00000335153 ENSP00000251849 ENSP00000302486 ENSP00000366244 |
| 5 | ENSP00000355153 ENSP00000417281 ENSP00000270202 ENSP00000366563 |
| 3 | ENSP00000355153 ENSP00000269305 ENSP00000267868 ENSP00000369497 |
| 12 | ENSP00000355153 ENSP00000417281 ENSP00000270202 ENSP00000348986 ENSP00000375892 |
| 3 | ENSP00000355153 ENSP00000269305 ENSP00000262367 ENSP00000384273 |
| 8 | ENSP00000355249 ENSP00000267163 ENSP00000227507 ENSP00000344818 ENSP00000364133 ENSP00000351905 ENSP00000355896 |
| 4 | ENSP00000355249 ENSP00000267163 ENSP00000227507 ENSP00000344818 ENSP00000358622 |
| 4 | ENSP00000355249 ENSP00000267163 ENSP00000362649 ENSP00000384273 ENSP00000359424 |
| 5 | ENSP00000355249 ENSP00000267163 ENSP00000251849 ENSP00000309845 ENSP00000361120 |
| 5 | ENSP00000355249 ENSP00000267163 ENSP00000266970 ENSP00000269305 ENSP00000338018 ENSP00000361125 |
| 4 | ENSP00000355249 ENSP00000267163 ENSP00000227507 ENSP00000344818 ENSP00000364133 |
| 15 | ENSP00000355249 ENSP00000267163 ENSP00000251849 ENSP00000302486 ENSP00000366244 |
| 6 | ENSP00000355249 ENSP00000267163 ENSP00000417281 ENSP00000270202 ENSP00000366563 |
| 5 | ENSP00000355249 ENSP00000267163 ENSP00000266970 ENSP00000269305 ENSP00000267868 ENSP00000369497 |
| 13 | ENSP00000355249 ENSP00000267163 ENSP00000417281 ENSP00000270202 ENSP00000348986 ENSP00000375892 |
| 3 | ENSP00000355249 ENSP00000267163 ENSP00000362649 ENSP00000384273 |
| 6 | ENSP00000355896 ENSP00000351905 ENSP00000364133 ENSP00000344818 ENSP00000358622 |
| 7 | ENSP00000355896 ENSP00000351905 ENSP00000364133 ENSP00000344818 ENSP00000216797 ENSP00000359424 |
| 10 | ENSP00000355896 ENSP00000351905 ENSP00000364133 ENSP00000344818 ENSP00000206249 ENSP00000335153 ENSP00000251849 ENSP00000309845 ENSP00000361120 |
| 7 | ENSP00000355896 ENSP00000351905 ENSP00000364133 ENSP00000344818 ENSP00000338018 ENSP00000361125 |
| 4 | ENSP00000355896 ENSP00000351905 ENSP00000364133 |
| 19 | ENSP00000355896 ENSP00000351905 ENSP00000364133 ENSP00000344818 ENSP00000360266 ENSP00000215832 ENSP00000302486 ENSP00000366244 |
| 9 | ENSP00000355896 ENSP00000351905 ENSP00000364133 ENSP00000344818 ENSP00000270202 ENSP00000366563 |
| 7 | ENSP00000355896 ENSP00000351905 ENSP00000364133 ENSP00000344818 ENSP00000350283 ENSP00000369497 |
| 15 | ENSP00000355896 ENSP00000351905 ENSP00000364133 ENSP00000344818 ENSP00000264033 ENSP00000274335 ENSP00000303830 ENSP00000348986 ENSP00000375892 |
| 6 | ENSP00000355896 ENSP00000351905 ENSP00000364133 ENSP00000344818 ENSP00000384273 |
| 1 | ENSP00000358622 ENSP00000359424 |
| 6 | ENSP00000358622 ENSP00000344818 ENSP00000206249 ENSP00000335153 ENSP00000251849 ENSP00000309845 ENSP00000361120 |
| 3 | ENSP00000358622 ENSP00000344818 ENSP00000338018 ENSP00000361125 |
| 2 | ENSP00000358622 ENSP00000344818 ENSP00000364133 |
| 15 | ENSP00000358622 ENSP00000344818 ENSP00000360266 ENSP00000215832 ENSP00000302486 ENSP00000366244 |
| 5 | ENSP00000358622 ENSP00000344818 ENSP00000270202 ENSP00000366563 |
| 3 | ENSP00000358622 ENSP00000344818 ENSP00000350283 ENSP00000369497 |
| 11 | ENSP00000358622 ENSP00000344818 ENSP00000264033 ENSP00000274335 ENSP00000303830 ENSP00000348986 ENSP00000375892 |
| 2 | ENSP00000358622 ENSP00000216797 ENSP00000384273 |
| 7 | ENSP00000359424 ENSP00000384273 ENSP00000362649 ENSP00000267163 ENSP00000251849 ENSP00000309845 ENSP00000361120 |
| 4 | ENSP00000359424 ENSP00000384273 ENSP00000262367 ENSP00000338018 ENSP00000361125 |
| 3 | ENSP00000359424 ENSP00000216797 ENSP00000344818 ENSP00000364133 |
| 16 | ENSP00000359424 ENSP00000384273 ENSP00000263253 ENSP00000360266 ENSP00000215832 ENSP00000302486 ENSP00000366244 |
| 6 | ENSP00000359424 ENSP00000270202 ENSP00000366563 |
| 4 | ENSP00000359424 ENSP00000216797 ENSP00000344818 ENSP00000350283 ENSP00000369497 |
| 12 | ENSP00000359424 ENSP00000216797 ENSP00000344818 ENSP00000264033 ENSP00000274335 ENSP00000303830 ENSP00000348986 ENSP00000375892 |
| 1 | ENSP00000359424 ENSP00000384273 |
| 5 | ENSP00000361120 ENSP00000309845 ENSP00000251849 ENSP00000335153 ENSP00000338018 ENSP00000361125 |
| 6 | ENSP00000361120 ENSP00000309845 ENSP00000251849 ENSP00000335153 ENSP00000206249 ENSP00000344818 ENSP00000364133 |
| 14 | ENSP00000361120 ENSP00000309845 ENSP00000251849 ENSP00000302486 ENSP00000366244 |
| 7 | ENSP00000361120 ENSP00000309845 ENSP00000263967 ENSP00000270202 ENSP00000366563 |
| 6 | ENSP00000361120 ENSP00000309845 ENSP00000251849 ENSP00000335153 ENSP00000269305 ENSP00000267868 ENSP00000369497 |
| 11 | ENSP00000361120 ENSP00000309845 ENSP00000263967 ENSP00000274335 ENSP00000303830 ENSP00000348986 ENSP00000375892 |
| 6 | ENSP00000361120 ENSP00000309845 ENSP00000251849 ENSP00000335153 ENSP00000206249 ENSP00000262367 ENSP00000384273 |
| 3 | ENSP00000361125 ENSP00000338018 ENSP00000344818 ENSP00000364133 |
| 15 | ENSP00000361125 ENSP00000338018 ENSP00000335153 ENSP00000251849 ENSP00000302486 ENSP00000366244 |
| 6 | ENSP00000361125 ENSP00000338018 ENSP00000335153 ENSP00000270202 ENSP00000366563 |
| 4 | ENSP00000361125 ENSP00000338018 ENSP00000269305 ENSP00000267868 ENSP00000369497 |
| 12 | ENSP00000361125 ENSP00000338018 ENSP00000344818 ENSP00000264033 ENSP00000274335 ENSP00000303830 ENSP00000348986 ENSP00000375892 |
| 3 | ENSP00000361125 ENSP00000338018 ENSP00000262367 ENSP00000384273 |
| 15 | ENSP00000364133 ENSP00000344818 ENSP00000360266 ENSP00000215832 ENSP00000302486 ENSP00000366244 |
| 5 | ENSP00000364133 ENSP00000344818 ENSP00000270202 ENSP00000366563 |
| 3 | ENSP00000364133 ENSP00000344818 ENSP00000350283 ENSP00000369497 |
| 11 | ENSP00000364133 ENSP00000344818 ENSP00000264033 ENSP00000274335 ENSP00000303830 ENSP00000348986 ENSP00000375892 |
| 2 | ENSP00000364133 ENSP00000344818 ENSP00000384273 |
| 17 | ENSP00000366244 ENSP00000302486 ENSP00000251849 ENSP00000335153 ENSP00000270202 ENSP00000366563 |
| 16 | ENSP00000366244 ENSP00000302486 ENSP00000251849 ENSP00000335153 ENSP00000269305 ENSP00000267868 ENSP00000369497 |
| 23 | ENSP00000366244 ENSP00000302486 ENSP00000251849 ENSP00000309845 ENSP00000263967 ENSP00000274335 ENSP00000303830 ENSP00000348986 ENSP00000375892 |
| 15 | ENSP00000366244 ENSP00000302486 ENSP00000215832 ENSP00000360266 ENSP00000263253 ENSP00000384273 |
| 6 | ENSP00000366563 ENSP00000270202 ENSP00000344818 ENSP00000350283 ENSP00000369497 |
| 13 | ENSP00000366563 ENSP00000270202 ENSP00000348986 ENSP00000375892 |
| 5 | ENSP00000366563 ENSP00000270202 ENSP00000344818 ENSP00000384273 |
| 12 | ENSP00000369497 ENSP00000350283 ENSP00000344818 ENSP00000264033 ENSP00000274335 ENSP00000303830 ENSP00000348986 ENSP00000375892 |
| 3 | ENSP00000369497 ENSP00000350283 ENSP00000344818 ENSP00000384273 |
| 11 | ENSP00000375892 ENSP00000348986 ENSP00000303830 ENSP00000274335 ENSP00000264033 ENSP00000344818 ENSP00000384273 |
