# Supplementary material for: Mining for Candidate Genes Related to Pancreatic Cancer Using Protein-Protein Interactions and a Shortest Path Approach
Source: Biomed Res Int. 2015 Nov 3;2015:623121. doi: 10.1155/2015/623121 (PMC4647023; doi:10.1155/2015/623121)
Supplement: Supplementary file 1 — Supplementary Material I: lists 65 PC-related genes and their ensembl IDs. Supplementary Material II: lists the detailed information of 2,080 shortest paths. Supplementary Material III: lists edges in a graph consisting of shortest paths connecting any two PC-related genes. Supplementary Material IV: lists 69 shortest path genes and their betweenness and permutation FDRs. [file 623121.f1.zip › Supplementary Material III.docx]

**Supplementary Material III.** Edges in a graph consisting of shortest paths connecting any two PC-related genes

| Node #1 | Node #2 | Weight |
| --- | --- | --- |
| ENSP00000003084 | ENSP00000262613 | 1 |
| ENSP00000003084 | ENSP00000344818 | 1 |
| ENSP00000262613 | ENSP00000338934 | 1 |
| ENSP00000344818 | ENSP00000345571 | 1 |
| ENSP00000344818 | ENSP00000347858 | 1 |
| ENSP00000344818 | ENSP00000350283 | 1 |
| ENSP00000344818 | ENSP00000358022 | 1 |
| ENSP00000344818 | ENSP00000358622 | 1 |
| ENSP00000344818 | ENSP00000360266 | 1 |
| ENSP00000344818 | ENSP00000364133 | 1 |
| ENSP00000344818 | ENSP00000384273 | 1 |
| ENSP00000005257 | ENSP00000019317 | 1 |
| ENSP00000005257 | ENSP00000342793 | 2 |
| ENSP00000005257 | ENSP00000349467 | 10 |
| ENSP00000019317 | ENSP00000272519 | 1 |
| ENSP00000046794 | ENSP00000244007 | 1 |
| ENSP00000046794 | ENSP00000302269 | 1 |
| ENSP00000244007 | ENSP00000261799 | 1 |
| ENSP00000244007 | ENSP00000264033 | 1 |
| ENSP00000244007 | ENSP00000275493 | 1 |
| ENSP00000302269 | ENSP00000304283 | 6 |
| ENSP00000302269 | ENSP00000339007 | 1 |
| ENSP00000302269 | ENSP00000348461 | 2 |
| ENSP00000162330 | ENSP00000228307 | 1 |
| ENSP00000162330 | ENSP00000350941 | 1 |
| ENSP00000162330 | ENSP00000360683 | 1 |
| ENSP00000228307 | ENSP00000299421 | 1 |
| ENSP00000228307 | ENSP00000300574 | 1 |
| ENSP00000228307 | ENSP00000341189 | 1 |
| ENSP00000350941 | ENSP00000360683 | 1 |
| ENSP00000206249 | ENSP00000227507 | 1 |
| ENSP00000206249 | ENSP00000262367 | 1 |
| ENSP00000206249 | ENSP00000263253 | 1 |
| ENSP00000206249 | ENSP00000335153 | 1 |
| ENSP00000206249 | ENSP00000344818 | 1 |
| ENSP00000206249 | ENSP00000350283 | 1 |
| ENSP00000227507 | ENSP00000244741 | 1 |
| ENSP00000227507 | ENSP00000257904 | 1 |
| ENSP00000227507 | ENSP00000264657 | 1 |
| ENSP00000227507 | ENSP00000265734 | 1 |
| ENSP00000227507 | ENSP00000267163 | 1 |
| ENSP00000227507 | ENSP00000344456 | 1 |
| ENSP00000227507 | ENSP00000344818 | 1 |
| ENSP00000262367 | ENSP00000269305 | 1 |
| ENSP00000262367 | ENSP00000320940 | 1 |
| ENSP00000262367 | ENSP00000338018 | 1 |
| ENSP00000262367 | ENSP00000354394 | 1 |
| ENSP00000262367 | ENSP00000384273 | 1 |
| ENSP00000263253 | ENSP00000264657 | 1 |
| ENSP00000263253 | ENSP00000269305 | 1 |
| ENSP00000263253 | ENSP00000329357 | 1 |
| ENSP00000263253 | ENSP00000332973 | 1 |
| ENSP00000263253 | ENSP00000338018 | 1 |
| ENSP00000263253 | ENSP00000341551 | 1 |
| ENSP00000263253 | ENSP00000354394 | 1 |
| ENSP00000263253 | ENSP00000360266 | 1 |
| ENSP00000263253 | ENSP00000384273 | 1 |
| ENSP00000335153 | ENSP00000338018 | 1 |
| ENSP00000350283 | ENSP00000369497 | 1 |
| ENSP00000215832 | ENSP00000219476 | 3 |
| ENSP00000215832 | ENSP00000269305 | 3 |
| ENSP00000215832 | ENSP00000302486 | 1 |
| ENSP00000215832 | ENSP00000360266 | 1 |
| ENSP00000219476 | ENSP00000263826 | 35 |
| ENSP00000219476 | ENSP00000270202 | 1 |
| ENSP00000219476 | ENSP00000300161 | 1 |
| ENSP00000219476 | ENSP00000309503 | 1 |
| ENSP00000219476 | ENSP00000354558 | 2 |
| ENSP00000269305 | ENSP00000278616 | 1 |
| ENSP00000269305 | ENSP00000302564 | 3 |
| ENSP00000269305 | ENSP00000321410 | 2 |
| ENSP00000269305 | ENSP00000329357 | 1 |
| ENSP00000269305 | ENSP00000329623 | 1 |
| ENSP00000269305 | ENSP00000335153 | 1 |
| ENSP00000269305 | ENSP00000338018 | 1 |
| ENSP00000269305 | ENSP00000344818 | 1 |
| ENSP00000269305 | ENSP00000353483 | 1 |
| ENSP00000269305 | ENSP00000355153 | 1 |
| ENSP00000302486 | ENSP00000366244 | 11 |
| ENSP00000216797 | ENSP00000226574 | 1 |
| ENSP00000216797 | ENSP00000339151 | 1 |
| ENSP00000216797 | ENSP00000344818 | 1 |
| ENSP00000216797 | ENSP00000358622 | 1 |
| ENSP00000216797 | ENSP00000359424 | 1 |
| ENSP00000216797 | ENSP00000384273 | 1 |
| ENSP00000226574 | ENSP00000263253 | 2 |
| ENSP00000226574 | ENSP00000359206 | 1 |
| ENSP00000226574 | ENSP00000384273 | 1 |
| ENSP00000339151 | ENSP00000344818 | 1 |
| ENSP00000339151 | ENSP00000358622 | 1 |
| ENSP00000339151 | ENSP00000359424 | 1 |
| ENSP00000339151 | ENSP00000384273 | 1 |
| ENSP00000358622 | ENSP00000359424 | 1 |
| ENSP00000359424 | ENSP00000384273 | 1 |
| ENSP00000270202 | ENSP00000289153 | 2 |
| ENSP00000270202 | ENSP00000297494 | 3 |
| ENSP00000270202 | ENSP00000299421 | 2 |
| ENSP00000270202 | ENSP00000309103 | 2 |
| ENSP00000270202 | ENSP00000335153 | 1 |
| ENSP00000270202 | ENSP00000344818 | 1 |
| ENSP00000270202 | ENSP00000348461 | 2 |
| ENSP00000270202 | ENSP00000348986 | 4 |
| ENSP00000270202 | ENSP00000352121 | 2 |
| ENSP00000270202 | ENSP00000354558 | 1 |
| ENSP00000270202 | ENSP00000359424 | 3 |
| ENSP00000270202 | ENSP00000366563 | 3 |
| ENSP00000270202 | ENSP00000417281 | 1 |
| ENSP00000221930 | ENSP00000351905 | 1 |
| ENSP00000221930 | ENSP00000364133 | 1 |
| ENSP00000351905 | ENSP00000355896 | 3 |
| ENSP00000351905 | ENSP00000364133 | 1 |
| ENSP00000222005 | ENSP00000257904 | 1 |
| ENSP00000222005 | ENSP00000335153 | 1 |
| ENSP00000257904 | ENSP00000267163 | 1 |
| ENSP00000257904 | ENSP00000355153 | 1 |
| ENSP00000222254 | ENSP00000261799 | 1 |
| ENSP00000222254 | ENSP00000263967 | 1 |
| ENSP00000261799 | ENSP00000274335 | 1 |
| ENSP00000261799 | ENSP00000288986 | 2 |
| ENSP00000261799 | ENSP00000339007 | 2 |
| ENSP00000263967 | ENSP00000270202 | 2 |
| ENSP00000263967 | ENSP00000274335 | 1 |
| ENSP00000263967 | ENSP00000304895 | 1 |
| ENSP00000263967 | ENSP00000309845 | 1 |
| ENSP00000223023 | ENSP00000314458 | 1 |
| ENSP00000223023 | ENSP00000339007 | 1 |
| ENSP00000339007 | ENSP00000341189 | 1 |
| ENSP00000339007 | ENSP00000361423 | 1 |
| ENSP00000339007 | ENSP00000384675 | 1 |
| ENSP00000244741 | ENSP00000257904 | 1 |
| ENSP00000244741 | ENSP00000265734 | 1 |
| ENSP00000244741 | ENSP00000269305 | 1 |
| ENSP00000264657 | ENSP00000275493 | 1 |
| ENSP00000264657 | ENSP00000343204 | 1 |
| ENSP00000264657 | ENSP00000348461 | 4 |
| ENSP00000264657 | ENSP00000350941 | 1 |
| ENSP00000264657 | ENSP00000354394 | 2 |
| ENSP00000264657 | ENSP00000354558 | 2 |
| ENSP00000265734 | ENSP00000267163 | 1 |
| ENSP00000265734 | ENSP00000355153 | 1 |
| ENSP00000267163 | ENSP00000345571 | 1 |
| ENSP00000267163 | ENSP00000355249 | 1 |
| ENSP00000267163 | ENSP00000361423 | 2 |
| ENSP00000267163 | ENSP00000362649 | 1 |
| ENSP00000267163 | ENSP00000417281 | 1 |
| ENSP00000344456 | ENSP00000359206 | 1 |
| ENSP00000299421 | ENSP00000384515 | 2 |
| ENSP00000228872 | ENSP00000257904 | 1 |
| ENSP00000228872 | ENSP00000265734 | 1 |
| ENSP00000228872 | ENSP00000270202 | 2 |
| ENSP00000238682 | ENSP00000351905 | 1 |
| ENSP00000238682 | ENSP00000364133 | 1 |
| ENSP00000264033 | ENSP00000274335 | 1 |
| ENSP00000264033 | ENSP00000275493 | 1 |
| ENSP00000264033 | ENSP00000288986 | 1 |
| ENSP00000264033 | ENSP00000300574 | 1 |
| ENSP00000264033 | ENSP00000302269 | 1 |
| ENSP00000264033 | ENSP00000339007 | 1 |
| ENSP00000264033 | ENSP00000344818 | 1 |
| ENSP00000264033 | ENSP00000350941 | 1 |
| ENSP00000264033 | ENSP00000361423 | 1 |
| ENSP00000275493 | ENSP00000295400 | 1 |
| ENSP00000275493 | ENSP00000339007 | 1 |
| ENSP00000275493 | ENSP00000340944 | 1 |
| ENSP00000275493 | ENSP00000344818 | 1 |
| ENSP00000275493 | ENSP00000350941 | 1 |
| ENSP00000275493 | ENSP00000354394 | 1 |
| ENSP00000275493 | ENSP00000384675 | 2 |
| ENSP00000249071 | ENSP00000269321 | 1 |
| ENSP00000269321 | ENSP00000304283 | 4 |
| ENSP00000269321 | ENSP00000314458 | 1 |
| ENSP00000269321 | ENSP00000348461 | 1 |
| ENSP00000250617 | ENSP00000384515 | 3 |
| ENSP00000250894 | ENSP00000352157 | 3 |
| ENSP00000250894 | ENSP00000353483 | 3 |
| ENSP00000353483 | ENSP00000360266 | 1 |
| ENSP00000251849 | ENSP00000267163 | 2 |
| ENSP00000251849 | ENSP00000288602 | 1 |
| ENSP00000251849 | ENSP00000300161 | 1 |
| ENSP00000251849 | ENSP00000302486 | 1 |
| ENSP00000251849 | ENSP00000309503 | 1 |
| ENSP00000251849 | ENSP00000309845 | 1 |
| ENSP00000251849 | ENSP00000335153 | 1 |
| ENSP00000288602 | ENSP00000309845 | 2 |
| ENSP00000309845 | ENSP00000350941 | 3 |
| ENSP00000309845 | ENSP00000352121 | 5 |
| ENSP00000309845 | ENSP00000361120 | 1 |
| ENSP00000309845 | ENSP00000384675 | 1 |
| ENSP00000254066 | ENSP00000268058 | 1 |
| ENSP00000254066 | ENSP00000284384 | 8 |
| ENSP00000254066 | ENSP00000320940 | 1 |
| ENSP00000268058 | ENSP00000269305 | 1 |
| ENSP00000284384 | ENSP00000338934 | 6 |
| ENSP00000284384 | ENSP00000342793 | 3 |
| ENSP00000355153 | ENSP00000417281 | 1 |
| ENSP00000274335 | ENSP00000289153 | 5 |
| ENSP00000274335 | ENSP00000300574 | 2 |
| ENSP00000274335 | ENSP00000303830 | 1 |
| ENSP00000274335 | ENSP00000304895 | 1 |
| ENSP00000274335 | ENSP00000339007 | 1 |
| ENSP00000262160 | ENSP00000262367 | 1 |
| ENSP00000262160 | ENSP00000262435 | 1 |
| ENSP00000262160 | ENSP00000329357 | 1 |
| ENSP00000262160 | ENSP00000341551 | 1 |
| ENSP00000262160 | ENSP00000344818 | 1 |
| ENSP00000262160 | ENSP00000364133 | 1 |
| ENSP00000262435 | ENSP00000332973 | 1 |
| ENSP00000329357 | ENSP00000341551 | 1 |
| ENSP00000329357 | ENSP00000345571 | 1 |
| ENSP00000341551 | ENSP00000344818 | 1 |
| ENSP00000338018 | ENSP00000344818 | 1 |
| ENSP00000338018 | ENSP00000361125 | 1 |
| ENSP00000354394 | ENSP00000371067 | 1 |
| ENSP00000332973 | ENSP00000341551 | 1 |
| ENSP00000332973 | ENSP00000344818 | 1 |
| ENSP00000332973 | ENSP00000360266 | 2 |
| ENSP00000262741 | ENSP00000263967 | 1 |
| ENSP00000262904 | ENSP00000267163 | 1 |
| ENSP00000263025 | ENSP00000302486 | 1 |
| ENSP00000304895 | ENSP00000339007 | 1 |
| ENSP00000304895 | ENSP00000343204 | 1 |
| ENSP00000304895 | ENSP00000353483 | 2 |
| ENSP00000304895 | ENSP00000371067 | 1 |
| ENSP00000343204 | ENSP00000354394 | 2 |
| ENSP00000265171 | ENSP00000267101 | 2 |
| ENSP00000265171 | ENSP00000269571 | 2 |
| ENSP00000265171 | ENSP00000275493 | 1 |
| ENSP00000265171 | ENSP00000339007 | 2 |
| ENSP00000267101 | ENSP00000269571 | 1 |
| ENSP00000267101 | ENSP00000274335 | 1 |
| ENSP00000269571 | ENSP00000275493 | 1 |
| ENSP00000269571 | ENSP00000335153 | 1 |
| ENSP00000269571 | ENSP00000339007 | 1 |
| ENSP00000269571 | ENSP00000344818 | 1 |
| ENSP00000269571 | ENSP00000350941 | 1 |
| ENSP00000266970 | ENSP00000267163 | 1 |
| ENSP00000266970 | ENSP00000269305 | 1 |
| ENSP00000362649 | ENSP00000384273 | 1 |
| ENSP00000267868 | ENSP00000269305 | 1 |
| ENSP00000267868 | ENSP00000369497 | 1 |
| ENSP00000268182 | ENSP00000314458 | 1 |
| ENSP00000268182 | ENSP00000348461 | 1 |
| ENSP00000269300 | ENSP00000352121 | 11 |
| ENSP00000278616 | ENSP00000287647 | 1 |
| ENSP00000278616 | ENSP00000361423 | 1 |
| ENSP00000302564 | ENSP00000309103 | 1 |
| ENSP00000321410 | ENSP00000360266 | 1 |
| ENSP00000304283 | ENSP00000350941 | 8 |
| ENSP00000297494 | ENSP00000335153 | 3 |
| ENSP00000297494 | ENSP00000349467 | 2 |
| ENSP00000309103 | ENSP00000309503 | 1 |
| ENSP00000309103 | ENSP00000329623 | 1 |
| ENSP00000348986 | ENSP00000375892 | 6 |
| ENSP00000303830 | ENSP00000304895 | 1 |
| ENSP00000303830 | ENSP00000340944 | 1 |
| ENSP00000303830 | ENSP00000348986 | 1 |
| ENSP00000303830 | ENSP00000360683 | 1 |
| ENSP00000278568 | ENSP00000288986 | 1 |
| ENSP00000278568 | ENSP00000314458 | 1 |
| ENSP00000278568 | ENSP00000348461 | 1 |
| ENSP00000287647 | ENSP00000369497 | 1 |
| ENSP00000282561 | ENSP00000284384 | 6 |
| ENSP00000282561 | ENSP00000350941 | 3 |
| ENSP00000293288 | ENSP00000302564 | 1 |
| ENSP00000293288 | ENSP00000358022 | 1 |
| ENSP00000298316 | ENSP00000342793 | 5 |
| ENSP00000298316 | ENSP00000348461 | 6 |
| ENSP00000330237 | ENSP00000347858 | 1 |
