# Supplementary material for: Mining for Candidate Genes Related to Pancreatic Cancer Using Protein-Protein Interactions and a Shortest Path Approach
Source: Biomed Res Int. 2015 Nov 3;2015:623121. doi: 10.1155/2015/623121 (PMC4647023; doi:10.1155/2015/623121)
Supplement: Supplementary file 1 — Supplementary Material I: lists 65 PC-related genes and their ensembl IDs. Supplementary Material II: lists the detailed information of 2,080 shortest paths. Supplementary Material III: lists edges in a graph consisting of shortest paths connecting any two PC-related genes. Supplementary Material IV: lists 69 shortest path genes and their betweenness and permutation FDRs. [file 623121.f1.zip › Supplementary Material IV.docx]

**Supplementary Material IV.** 69 shortest path genes and their betweenness and permutation FDRs

| **Ensembl ID** | **Gene symbol** | **Betweenness** | **Permutation FDR** |
| --- | --- | --- | --- |
| ENSP00000384515 | PARVB | 64 | <0.001 |
| ENSP00000284384 | PRKCA | 175 | <0.001 |
| ENSP00000250894 | MAPK8IP3 | 64 | <0.001 |
| ENSP00000216797 | NFKBIA | 73 | 0.002 |
| ENSP00000254066 | RARA | 24 | 0.005 |
| ENSP00000347858 | XIAP | 64 | 0.006 |
| ENSP00000335153 | HSP90AA1 | 230 | 0.007 |
| ENSP00000269321 | ARHGDIA | 94 | 0.012 |
| ENSP00000338934 | EZR | 128 | 0.026 |
| ENSP00000299421 | ILK | 64 | 0.03 |
| ENSP00000348986 | INS-IGF2 | 64 | 0.031 |
| ENSP00000262613 | SLC9A3R1 | 128 | 0.031 |
| ENSP00000309845 | HRAS | 113 | 0.033 |
| ENSP00000268182 | IQGAP1 | 12 | 0.05 |
| ENSP00000304895 | IRS1 | 40 | 0.053 |
| ENSP00000244741 | CDKN1A | 22 | 0.063 |
| ENSP00000262435 | SMURF2 | 1 | 0.071 |
| ENSP00000219476 | TSC2 | 64 | 0.077 |
| ENSP00000359206 | BTRC | 3 | 0.094 |
| ENSP00000293288 | BAX | 28 | 0.103 |
| ENSP00000261799 | PDGFRB | 31 | 0.103 |
| ENSP00000223023 | WASL | 54 | 0.123 |
| ENSP00000268058 | PML | 20 | 0.124 |
| ENSP00000282561 | GJA1 | 23 | 0.138 |
| ENSP00000300161 | YWHAB | 6 | 0.143 |
| ENSP00000267101 | ERBB3 | 7 | 0.15 |
| ENSP00000222005 | CDC37 | 5 | 0.161 |
| ENSP00000278568 | PAK1 | 15 | 0.164 |
| ENSP00000302269 | VAV1 | 33 | 0.174 |
| ENSP00000288986 | NCK1 | 15 | 0.183 |
| ENSP00000360266 | JUN | 176 | 0.212 |
| ENSP00000329623 | BCL2 | 23 | 0.218 |
| ENSP00000384675 | SOS1 | 7 | 0.224 |
| ENSP00000287647 | FANCD2 | 1 | 0.237 |
| ENSP00000298316 | ARF6 | 31 | 0.269 |
| ENSP00000360683 | PTPN1 | 5 | 0.273 |
| ENSP00000303830 | INSR | 51 | 0.276 |
| ENSP00000003084 | CFTR | 128 | 0.294 |
| ENSP00000358022 | MCL1 | 28 | 0.336 |
| ENSP00000228872 | CDKN1B | 15 | 0.357 |
| ENSP00000309503 | YWHAZ | 17 | 0.368 |
| ENSP00000329357 | SP1 | 10 | 0.37 |
| ENSP00000340944 | PTPN11 | 3 | 0.392 |
| ENSP00000341189 | PTK2 | 2 | 0.393 |
| ENSP00000228307 | PXN | 9 | 0.409 |
| ENSP00000244007 | PLCG1 | 24 | 0.465 |
| ENSP00000349467 | CALM1 | 38 | 0.511 |
| ENSP00000362649 | HDAC1 | 13 | 0.545 |
| ENSP00000350283 | BRCA1 | 40 | 0.547 |
| ENSP00000300574 | CRK | 4 | 0.55 |
| ENSP00000297494 | NOS3 | 38 | 0.55 |
| ENSP00000320940 | NCOA1 | 4 | 0.557 |
| ENSP00000263253 | EP300 | 83 | 0.564 |
| ENSP00000339007 | GRB2 | 56 | 0.602 |
| ENSP00000354558 | MTOR | 5 | 0.622 |
| ENSP00000262367 | CREBBP | 55 | 0.633 |
| ENSP00000361423 | ABL1 | 6 | 0.657 |
| ENSP00000266970 | CDK2 | 22 | 0.696 |
| ENSP00000371067 | JAK2 | 4 | 0.703 |
| ENSP00000046794 | LCP2 | 1 | 0.723 |
| ENSP00000278616 | ATM | 2 | 0.749 |
| ENSP00000162330 | BCAR1 | 3 | 0.787 |
| ENSP00000338018 | HIF1A | 64 | 0.822 |
| ENSP00000350941 | SRC | 27 | 0.881 |
| ENSP00000417281 | MDM2 | 50 | 0.924 |
| ENSP00000264033 | CBL | 164 | 0.925 |
| ENSP00000344456 | CTNNB1 | 3 | 0.961 |
| ENSP00000344818 | UBC | 916 | 0.98 |
| ENSP00000206249 | ESR1 | 41 | 0.988 |
